# Exploring acupuncture as a treatment for insomnia in perimenopausal women with stable angina pectoris: A protocol for a randomized, double-blind, placebo-controlled clinical trial

**DOI:** 10.1371/journal.pone.0301827

**Published:** 2024-04-18

**Authors:** Rui Shi, Wenyi Meng, Zhaozheng Liu, Wen Xue, Xingyu Chen, Yue Deng

**Affiliations:** 1 Department of School of Traditional Chinese Medicine, Changchun University of Chinese Medicine, Changchun, China; 2 TCM Cardiovascular Clinical Medicine Research Center of Jilin Province, Changchun, China; 3 Department of Affiliated Hospital, Changchun University of Chinese Medicine, Changchun, China; The Second Affiliated Hospital of Shandong First Medical University, CHINA

## Abstract

**Background:**

Insomnia has emerged as a major public health issue jeopardizing human wellbeing. Furthermore, insomnia and angina arise concomitantly and exert reciprocal effects. Multiple studies suggest that perimenopausal females are more prone to experiencing both angina and insomnia, consequently substantially compromising their quality of life.Credible evidence suggests that acupuncture exerts a beneficial impact in alleviating insomnia. Nevertheless, the exhaustive investigation into the potential of acupuncture for mitigating insomnia co-occurring with stable angina in perimenopausal females remains a realm yet to be traversed in the realm of randomized controlled trials. Hence, the primary intent of this research protocol was to evaluate the effectiveness and safety profile of acupuncture when administered to perimenopausal subjects grappling with concomitant conditions of stable angina and insomnia.

**Methods:**

This study entails a single-center, randomized, double-blind, placebo-controlled clinical trial. A total of 110 patients exhibiting insomnia concomitant with stable angina in the perimenopausal period will be enlisted and randomized to either acupuncture or sham acupuncture. Participants in both arms will undergo 30-minute sessions thrice weekly over a 12-week intervention period, with a 12-week maximum follow-up. The primary outcome measure is the Pittsburgh Sleep Quality Index(PSQI). Secondary outcomes encompass the Health-Related Quality of Life Questionnaire (SF-36), Dosage of sleeping pills, SAP-associated evaluations, including C-reactive protein (CRP), lipoprotein-associated phospholipase A2 (Lp-PLA2), cardiac fatty acid-binding protein levels (C-FABP), and the Seattle Angina Questionnaire (SAQ). Additionally, the study includes assessments using the Hamilton Depression Inventory (HAMD) and the Generalized Anxiety Disorder Scale (GAD-7). Primary and secondary outcomes will be evaluated at baseline, 4 weeks, 8 weeks, 12 weeks (upon completion of the intervention), and at an additional 12-week follow-up. Any adverse events will be rigorously classified and characterized with respect to time of onset and abatement, therapeutic interventions implemented, impact on the primary morbidity, and regression.

**Discussion:**

The current study is poised to furnish pivotal clinical data on the utility of acupuncture for stable angina with concomitant insomnia in perimenopausal women, with the findings to be propagated through academic conferences and peer-reviewed publications.

**Clinical trial registration:**

Thai Clinical Trials Registry: TCTR20221121001. Registered 19 November 2022.

## Introduction

Stable angina pectoris (SAP), as a prevalent manifestation of coronary artery disease, exerts a pernicious impact on patients’ quality of life and portends a marked risk of progression to acute coronary syndromes [1–3]. In recent years, insomnia comorbid with SAP has garnered greater attention, with severe sleep disturbances emerging in numerous coronary artery disease patients [4, 5]. A preceding meta-analysis has demonstrated a robust correlation between insomnia and a heightened 45% susceptibility to cardiovascular disease morbidity and/or mortality in contrast to individuals exhibiting commendable sleep quality [6]. Given the precipitous decline in estrogen levels, perimenopausal women exhibit heightened vulnerability to coronary artery disease and insomnia [7].

The occurrence of angina pectoris may engender insomnia, which can adversely impact emotional regulation, cognition, memory, and systemic immunity, whilst also inflicting multi-organ damage including the cardiovascular system [8, 9]. In an 11-year longitudinal study, Sivertsen et al. identified insomnia as a salient risk factor for angina pectoris, cerebrovascular accident, and hypertension [10]. While sleep medicine research commenced in China in the 1980s, a formalized discipline remains nascent, and lay awareness of sleep hygiene remains negligible, with approximately 43% of Chinese citizens exhibiting varying degrees of sleep pathology, disproportionately afflicting women [11, 12]. The surging medical expenditures and diminished work productivity engendered by sleep disorders have emerged as pressing public health concerns [13].

Traditional physiotherapeutic modalities, behavioral therapeutic strategies, and psychotherapeutic interventions necessitate a manifold array of specialized personnel and material accoutrements, entailing an intricate interplay of multifarious determinants, encompassing even the specter of perilous remedial sequelae. Such comprehensive paradigms encounter formidable hurdles in their quest for pervasive adoption within clinical milieus. Pharmacological intervention, a modality ubiquitously resorted to, casts a prominent shadow within the therapeutic landscape. Among these, sedative agents emerge as the veritable cornerstone of clinical practice. Nonetheless, the protraction of drug administration over prolonged epochs exposes patients to the vicissitudes of drug reliance, counteractive resurgences, compulsive enthrallment, alongside a sundry constellation of untoward sequelae, underscored by the ominous propensity for withdrawal manifestations upon cessation [14]. In recent years, the clinical efficacy of acupuncture has garnered escalating recognition, with multiple studies elucidating its therapeutic utility for insomnia [15–17]. Acupuncture can enhance sleep quality by facilitating slow-wave sleep [18, 19], and it is also efficacious in the management of perimenopausal insomnia [20–23].

Recent investigations indicate a robust association between pneumonia induced by the 2019 novel coronavirus (2019-nCoV) and an elevated predisposition to angina and insomnia [24, 25]. While pertinent assessments of acupuncture’s efficacy in treating insomnia have been undertaken in the past, the onset of the 2019-nCoV pandemic has resulted in a significant paucity of current analyses of randomized controlled trials, especially concerning the specialized indication of concurrent angina and insomnia in perimenopausal women. In light of the paucity of data evaluating the efficacy and safety of acupuncture for insomnia specifically in perimenopausal patients with stable angina, we undertook this study.

## Materials and methods

### Trial strategy

**Fig 1:**
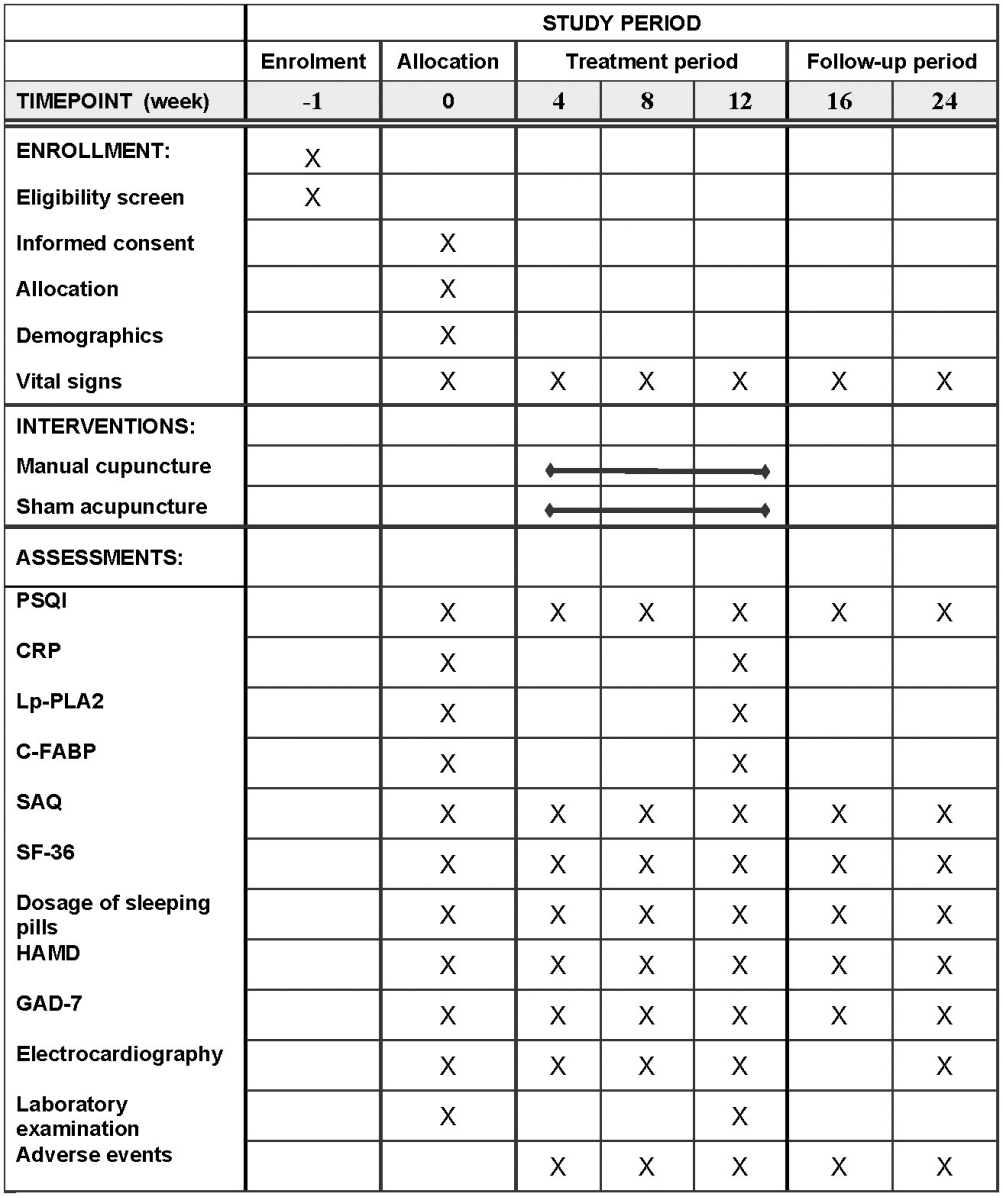
The SPIRIT schedule of enrollment, interventions, and assessment.

**Fig 2:**
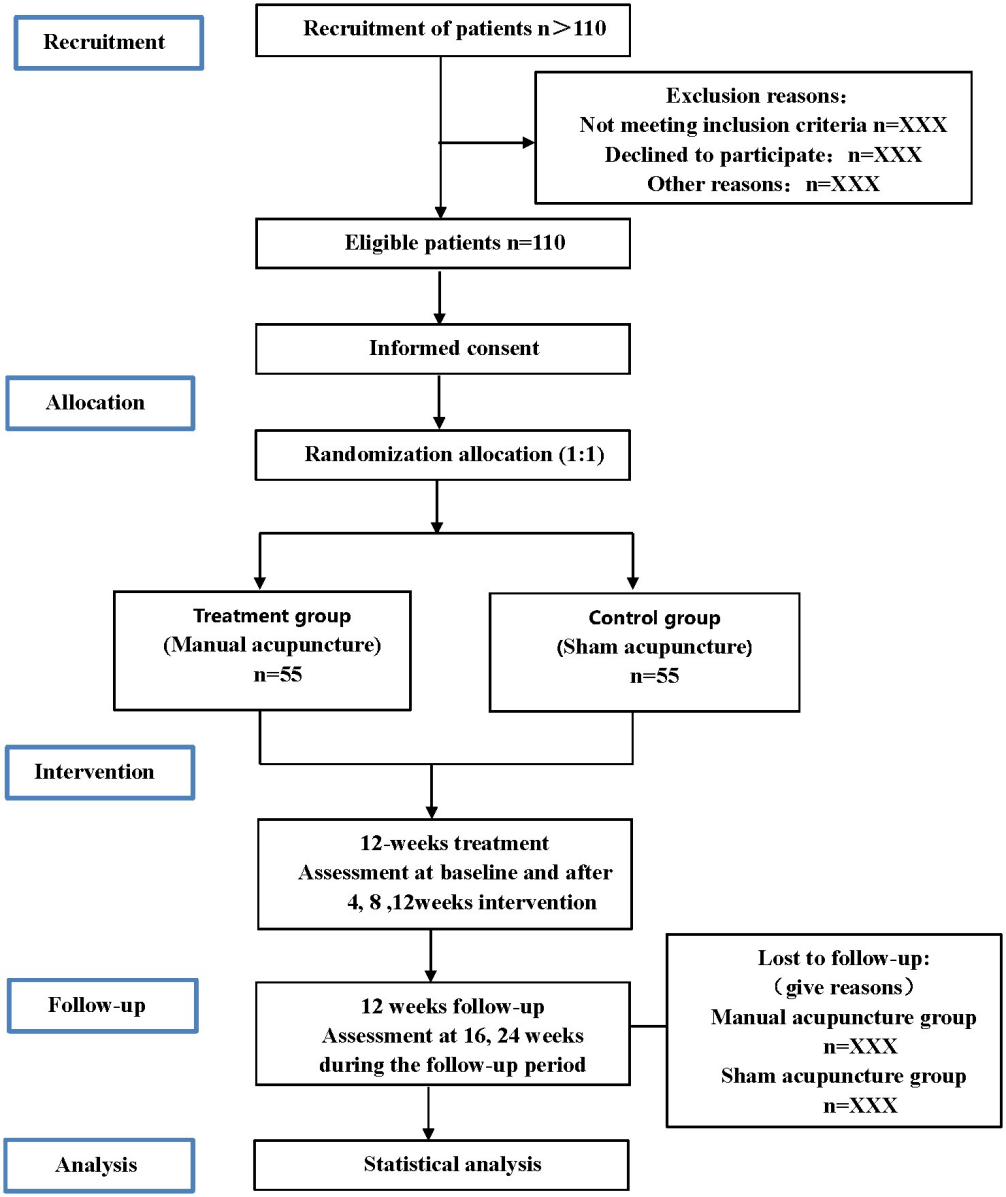
Flowchart of the trial.

A single-center randomized controlled trial has been designed to compare the effects of manual versus sham acupuncture in the management of insomnia comorbid with stable angina pectoris in perimenopausal women. The schedule for enrollment, interventions, and assessments is outlined in Fig 1, and the flowchart of the trial is shown in Fig 2. The SPIRIT (Standard Protocol Items: Recommendations for Interventional Trials) [26] checklist is delineated in the S1 Appendix.

### Patients

This trial is slated to commence in March 2024, concluding in December 2024, with a target enrollment of 110 patients exhibiting insomnia comorbid with perimenopausal stable angina. Recruitment advertisements shall be situated in clinical environments, public venues, and online platforms to ensure adequate sample accretion. Eligible subjects shall be apprised of the prospective benefits and potential risks of participation, with written Participant Information and Consent Form (S2 Appendix) obtained to guarantee voluntary enrollment. Participants shall be considered successfully recruited upon provision of signed informed consent. All patients will receive conventional medications (e.g. aspirin, rosuvastatin, trimetazidine), plus lifestyle measures including dietary modification, appropriate exercise, and risk factor management.

### Diagnostic criteria

1. The diagnosis of stable angina pectoris followed the 2019 ESC Guidelines for the diagnosis and treatment of chronic coronary syndromes [27]. Coronary artery disease was established via coronary angiography, CT angiography or positive treadmill exercise testing. Patients exhibited Canadian Cardiovascular Society grade II-III angina, with a history of ≥30 days of stable angina, 2–5 anginal attacks per week.
2. Insomnia was defined as Pittsburgh Sleep Quality Index >5 per the International

Classification of Sleep Disorders [28]. Questionnaires assessing insomnia duration and frequency were administered at admission by two blinded independent assessors, with consensus analysis to ensure consistent results. All enrolled patients exhibited self-reported insomnia of at least 30 days duration occurring ≥3 times per week.

### Inclusion criteria

1. Meet diagnostic criteria for both stable angina pectoris and insomnia as delineated above;
2. Female gender, 45–55 years of age, in the perimenopausal period;
3. No acupuncture treatment for minimum 6 months prior to enrollment;
4. Provide informed consent and agree to randomized group allocation.

### Exclusion criteria

1. Not in perimenopausal phase;
2. Severe renal, hepatic, hematologic or other systemic diseases;
3. Psychiatric disorders or impaired consciousness;
4. Uncontrolled severe hypertension, diabetes mellitus or malignant arrhythmia;
5. valvular disease, Recent acute myocardial infarction, unstable angina, myocarditis, hypertrophic cardiomyopathy, or post-pacemaker severe heart failure (NYHA grade ≥III);
6. Major surgery, trauma, bleeding event or severe infection within past 3 months;
7. Insomnia attributable to medications, alcohol or environmental factors;
8. Local skin infections near acupoint sites;
9. Malignancy or hyperthyroidism;
10. History of syncope or severe needle phobia;
11. Any other condition potentially interfering with study participation or completion as deemed by the investigator.

### Criteria for withdrawal and termination

Per ethical imperatives safeguarding patient autonomy, subjects retain the prerogative to withdraw from the study at any juncture for any reason. For subjects electing withdrawal, the rationale must be meticulously documented and incorporated as an evaluative metric for trial cessation. Clinical investigators should fastidiously document the basis for trial termination and its pertinence to the study, with analysis of the potential impact of the endpoint event on study conclusions.

All primary data on study withdrawals and terminations will remain sealed for examination upon trial completion. Moreover, grounds for termination include: any adverse events arising during the study; comorbidities, complications, or physiological alterations precluding ongoing participation; poor treatment compliance; contraindicated interventions; missed visits; and unblinding events.

### Randomization procedure and allocation concealment

In this trial, block randomization will be employed with a block size of 10. The research coordinator will generate randomization numbers independently utilizing SPSS 24.0 software without contacting study participants. To ensure allocation concealment, sequentially numbered opaque sealed envelopes will be employed, with an assigned designee independently administering the randomization sequence. Subsequent to informed consent, a total of 110 patients will be randomly allocated in a 1:1 ratio to the treatment arm (acupuncture group) or the control arm (sham acupuncture group).

### Blinding

**Fig 3:**
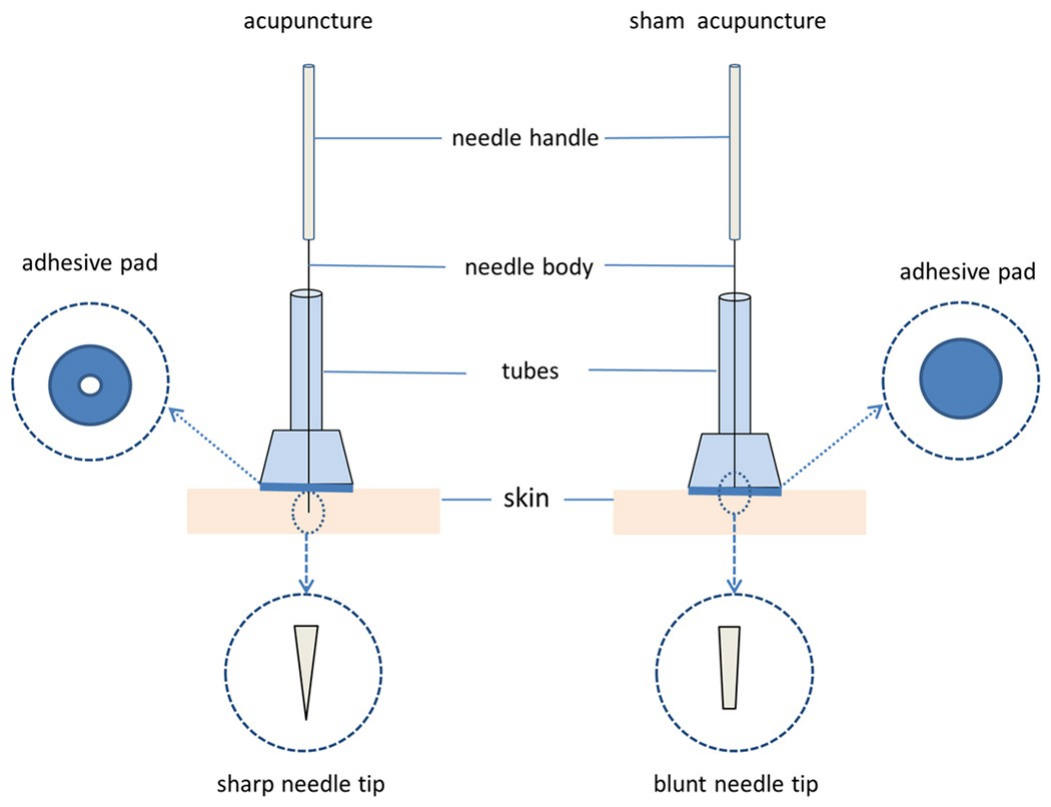
Schematic of the two groups of acupuncture and non-penetrating devices.

The allocation of groups will remain concealed from patients, evaluators, and data analysts. The acupuncture points and techniques will be comparable between the two groups. The control group will undergo sham acupuncture with a non-penetrating device mimicking the look of real needles to preserve blinding [29], see Fig 3. To evaluate the success of blinding, we will survey participants on their belief about receiving either genuine or sham acupuncture after the intervention. Due to the distinct nature of performing acupuncture, blinding the practitioners is not feasible.

### Interventions

Participants will undergo 36 acupuncture sessions over 12 weeks, at a frequency of 3 treatments per week. Acupuncture shall be administered solely by licensed acupuncturists. They are required to receive training in standardized techniques including needle manipulation and point location.

### Treatment group

Sterile acupuncture needles of 0.30 mm diameter and 40 mm length were utilized (Hualun Medical Appliance Co, Ltd, Suzhou). The acupuncture points were identified and localized conforming to the World Health Organization’s standards. The prescribed acupoints were PC6 (Neiguan), EX-HN3 (Yintang), KI6 (Zhaohai), RN17 (Danzhong), HT3 (Shaohai), and SP6 (Sanyinjiao). EX-HN3, and RN17 were inserted to a depth of 5-10mm, while KI6, PC6, HT3, and SP6 were inserted perpendicularly until reaching 20-25mm depth. The acupuncture procedure consisted of: disinfecting the skin and instruments; needle insertion at the acupoints, rotating bidirectionally between 90°-180° at 60–90 rotations/min until Deqi sensations of numbness, fullness, soreness, and aching were elicited, quantified using the Chinese Modified Massachusetts General Hospital Acupuncture Sensory Scale (C-MASS). Each 30-minute acupuncture session incorporated manual stimulation for 1 minute every 10 minutes.

### Control group

After performing the same sterilization procedures as the treatment group, the sham acupuncture group utilized blunt-ended needles (Hualun Medical Appliance Co, Ltd, Suzhou) of 0.30 mm diameter and 40 mm length to administer non-penetrative needling at non-acupoints (selected stimulation points close to the treatment group’s acupoints but outside the meridians and avoiding the distribution of the body’s nerves). Specific non-acupoints selection are shown in S1 Table. The treatment duration matched that of the treatment group, however the blunt needles (Fig 3) were only tapped lightly on the skin surface without tissue penetration or manipulation to elicit Deqi. Sham-needle stimulation was simulated for 1 minute at 10-minute intervals during the 30-minute treatment.

### Primary outcomes

The Pittsburgh Sleep Quality Index (PSQI), comprising 19 self-rated and 5 hetero-rated items, will be employed to evaluate the primary outcome of global sleep quality over the past month across 7 components: sleep duration, sleep latency, sleep efficiency, sleep medication use, sleep disturbances, subjective sleep quality, and daytime dysfunction. PSQI will be assessed at baseline, after each 4-week treatment phase (weeks 4, 8, 12), and during follow-up at weeks 16 and 24.

### Secondary outcomes

1. Assessment of stable angina pectoris (SAP): SAP was evaluated at baseline, week 4, week 8, and week 12 using the Seattle Angina Questionnaire (SAQ) to quantify angina symptoms before and after treatment. Blood biomarkers of SAP: Fasting venous blood samples were collected from the cubital fossa on the start and final of the treatment period in both groups. Levels of C-reactive protein (CRP), lipoprotein-associated phospholipase A2 (Lp-PLA2), and cardiac fatty acid-binding protein (C-FABP) were assayed by an independent laboratory blinded to group allocation.
2. Quality of life assessment using the 36-Item Short Form Health Survey (SF-36).
3. Dosage of sleeping pills.
4. Hamilton Depression Rating Scale (HAMD).
5. Generalized Anxiety Disorder 7-item (GAD-7) scale.

### Data gathering and administration

At screening, study personnel initiated collection of data on individuals meeting eligibility criteria, encompassing demographics (age, gender, occupation, ethnicity, marital status), anthropometrics (weight, height), education level, risk factors, medical history, medication history, and vital signs. During the intervention, study data were collected and documented per protocol via electronic transcription of paper case report forms, with data entry within 1 week of each study visit using EpiData software (version 3.0). Logical validation and double data entry with verification were implemented to ensure accuracy. An independent data manager external to the study oversaw data entry, verification, security, and resolution of abnormal values beyond clinically expected ranges. Finally, the database was locked for analysis after sign-off by the study sponsor, principal investigator, biostatistician, and data administrator, with no further modifications permitted.

### Patients’ safety and adverse events

Safety assessments were conducted at each study visit including: vital signs (temperature, blood pressure, respiration rate, heart rate); routine blood and urine tests; liver and renal function tests; electrocardiography. All adverse events were monitored, managed, and reported according to standard operating procedures detailing time of onset, duration, severity, interventions, and outcome. Adverse events will be collected according to the standardized methodology Common Terminology Criteria for Adverse Events (CTCAE). Trial suspension or withdrawal would be determined by the investigator contingent on adverse event severity. Any serious adverse event must be reported to the principal investigator and ethics committee within 24 hours.

### Sample size

The Pittsburgh Sleep Quality Index (PSQI) score following the full acupuncture intervention regimen was utilized to determine the requisite sample size. Statistical significance was defined as α = 0.05 with a power of 90%. Based on a prior study, perimenopausal women with insomnia had mean±SD PSQI scores of 11.43±2.11 with sham acupuncture and 9.20±3.24 with manual acupuncture [30]. Using the sample size formula:

$$n=2\times {\left[\;\frac{\left(Z_{\alpha }+Z_{\beta }\right)\times \sigma }{\delta }\;\right]}^{2}$$

Using the larger standard deviation of 3.24, with δ = 11.43–9.20 = 2.23, β = 0.1, 1-β = 0.9, and α = 0.05. The resultant sample size required per group was 44. Allowing for a 20% loss to follow-up, the total sample size needed for the study was 110 participants.

### Statistical analysis

Group allocation will be concealed from the statistician. All randomized participants will be included in the intention-to-treat (ITT) analysis, regardless of study completion status. To ensure accuracy, double data entry will be performed. Statistical analyses will be conducted using SPSS 24.0. Continuous variables will be summarized as mean ± standard deviation or median (interquartile range). Between-group comparisons will utilize two-sample t-tests or Wilcoxon signed-rank tests for skewed data. Within-group baseline to endpoint changes will be analyzed by repeated measure analysis of variance (ANOVA), with a primary focus on assessing interactions between groups and time. Categorical variables will be compared using Fisher’s exact or Chi-square tests. Statistical significance will be defined as p<0.05.

### Trial status

This trial is currently in the recruitment phase. Recruitment started in March 2024, and it is expected to finish in July 2024.

## Declarations

### Ethics approval and consent to participate

The study protocol was reviewed and approved by the Ethics Committee of Changchun University of Traditional Chinese Medicine Hospital (approval no. CCZYFYLL2022-029). The protocol was designed with careful consideration of patient welfare. All participants will provide written informed consent after being fully appraised of study purposes, procedures, and potential risks, in order to safeguard their rights and interests. Study findings will be disseminated through presentations at scientific conferences and publications in peer-reviewed journals. The study will be carried out in accordance with the Declaration of Helsinki and Good Clinical Practice guidelines.

## Discussion

In perimenopausal women, hormonal fluctuations and emotional changes can disrupt autonomic function and sleep-wake cycles [31]. A major health concern in this population is insomnia [32], which can exacerbate autonomic dysfunction, hypothalamic-pituitary-adrenal axis dysregulation, inflammation, blood pressure, heart rate, arrhythmias, and atherosclerotic plaque instability, markedly elevating cardiovascular risk [33–35]. Insomnia and stable angina pectoris (SAP) are frequently comorbid, and treating insomnia may mitigate SAP risk and severity as well as improve prognosis [5]. Current pharmacological management of perimenopausal insomnia relies heavily on sedative-hypnotics or hormonal supplements to correct endocrine abnormalities, however these medications confer considerable risks including memory deficits, dependence, and withdrawal reactions [36]. Contemporary research demonstrates that acupuncture can augment cerebral blood flow and activate key brain regions including the hypothalamus and temporal lobes to facilitate sleep [37]. Acupuncture may also regulate perimenopausal hormone levels to improve sleep quality [38, 39]. As a safe, natural intervention, numerous studies validate the benefits of acupuncture for insomnia.

However, the mechanisms underlying acupuncture treatment of insomnia and stable angina in perimenopausal women remain uncertain. This trial aims to investigate the potential of acupuncture as an effective therapy for perimenopausal women with insomnia and stable angina pectoris.

## Limitation

This study has several limitations. A major challenge in acupuncture trials is the inability to blind the acupuncturist, while elicitation of Deqi sensation is integral to efficacy, confounding participant blinding with current methods. Second, subtle variations in needling technique and localization are inherent between acupuncturists and individuals. Thus, extensive standardized pretrial training is imperative to minimize practitioner variability.

## Supporting information

S1 Appendix SPIRIT 2013 Checklist. (PDF)

S2 Appendix Participant information sheet/consent form. (PDF)

S1 File (PDF)

S2 File (PDF)

S1 Table Allocation of acupoints and non-acupoints. (PDF)
