# Supplementary material for: Exploring acupuncture as a treatment for insomnia in perimenopausal women with stable angina pectoris: A protocol for a randomized, double-blind, placebo-controlled clinical trial
Source: PLoS One. 2024 Apr 18;19(4):e0301827. doi: 10.1371/journal.pone.0301827 (PMC11025937; doi:10.1371/journal.pone.0301827)
Supplement: S2 Appendix — (PDF) [file pone.0301827.s002.pdf]

CCUCM
University

Participant Information Sheet/Consent Form

**Study Title:**	Acupuncture for perimenopausal stable angina pectoris with insomnia
**Program No.**	To Be Announced
**Sponsor:**	Changchun University of Chinese Medicine
**Clinical Research Unit:**	Changchun University of Chinese Medicine
**Principal Investigator:**	Rui Shi, Wenyi Meng, Zhaozheng Liu, Wen Xue, Xingyu Chen, Yue Deng

1 Introduction

We cordially extend an invitation for your participation in a single-center, randomized, double-blind, placebo-controlled trial assessing the efficacy and safety of acupuncture for the treatment of perimenopausal angina with insomnia. This informed consent document shall illuminate the objectives, protocols, benefits, risks, inconveniences, and discomforts associated with involvement in the study. We respectfully request your meticulous examination of the materials herein and thoughtful deliberation regarding enrollment. As the principal investigator elucidates the parameters of the trial, please freely interject with any queries so they may be addressed and clarified to your satisfaction. You may also consult with family, friends, and your physician when weighing your decision. We hope this informed consent facilitates an informed determination regarding your potential contribution to this clinical investigation.

If you elect to take part in this study, your signature denoting informed consent shall be required on the declaration at the conclusion of this document (with a duplicate copy furnished for your records). Participation in any study procedures may only commence once you have thoroughly reviewed and signed the informed consent form. Please notify the principal investigator if you are presently enrolled in another clinical trial.

2 Why this study was conducted?

The objective of this study is to closely examine the efficacy and safety of acupuncture for the treatment of perimenopausal angina with insomnia. Our research cohort has undertaken a series of preliminary investigations in recent years, and now

endeavors to further validate and expand our comprehension of this therapy via the current trial. Acupuncture, a long-standing medical tradition of Chinese origin, has historically demonstrated therapeutic benefit for manifold health afflictions. It is our aspiration that this study will furnish the medical community with additional substantiation of acupuncture's merit in contemporary medicine, whilst identifying an effective remedy for angina with insomnia among perimenopausal women.

3 Participant eligibility

If you would like to participate in this research project, you need to be female in gender, between 45-55 years of age, and in the perimenopausal period; have both stable angina and insomnia; have not received acupuncture treatment for at least 6 months prior to enrollment; and provide informed consent and agree to be randomized to a group.

Participation in the study was not possible if any of the following conditions existed. (i) not in perimenopause; (ii) severe renal, hepatic, hematologic, or other systemic diseases; (iii) mental disorders or disorders of consciousness; (iv) uncontrolled severe hypertension, diabetes mellitus, or malignant arrhythmia; (v) valvular disease, recent acute myocardial infarction, unstable angina, myocarditis, hypertrophic myocardial infarction, angina, myocardial infarction, myocarditis, hypertrophic cardiomyopathy, or severe post-pacemaker surgery heart failure (NYHA classification ≥III); vi) major surgery, trauma, bleeding events, or serious infections within the past 3 months; vii) insomnia due to drugs, alcohol, or environmental factors; viii) localized skin infections near the acupuncture points; ix) malignancy or hyperthyroidism; and x) history of syncope or severe phobia of needling.

4 Trial treatment and potential for random assignment to groups

This study employs a randomized design. Should you agree to participate, you will be randomly allocated to one of two study arms. One cohort shall receive verum acupuncture, while the other cohort shall receive sham acupuncture. This methodology is instituted to uphold objectivity and eliminate bias. Precisely half of participants will undergo needling at defined acupoints, while the remaining half will receive placebo needling. Neither you nor the investigators may dictate group assignment, which shall be randomized via computer to preclude any influence (akin to a "coin flip"—fully stochastic).

We appreciate your understanding that randomization is a crucial scientific principle for clinical trials. Please let us know if you have any other questions!

5 Steps you need to follow in a trial

The study encompasses a screening interval, a treatment interval, and a follow-up interval.
Eligibility screening shall transpire within 7 days antecedent to commencement of treatment. Subsequent to furnishing informed consent, the principal investigator shall gather particulars and execute a battery of physical examinations to authenticate your aptness for enrollment in the trial.

Screening: visit 1 (1 week before randomization to treatment) will require you to come to the research center and complete the following;

The screening appointment shall transpire within 1 week precedent to randomization. Prior to screening, the principal investigator shall furnish you with scripted particulars regarding the trial and study protocols as requested. You shall be comprehensively apprised, verbally and in writing, of your duties and privileges throughout enrollment, alongside potential benefits/risks of undergoing acupuncture. You may propound any queries, to be addressed individually by the principal investigator, and shall have ample time to contemplate participation.

If amenable to enrollment, you shall inscribe your signature and date upon the informed consent preceding any trial-related procedures, retaining a duplicate furnished by the research center displaying both your and the principal investigator's signatures, alongside your name, date, and contact evidence. The subsequent protocols must transpire at the screening appointment and/or be documented within the electronic case report form (eCRF):
- ✓ Signed Informed Consent Form
- ✓ Entry criteria
- ✓ Demographics
- ✓ Past medical history and comorbidities
- ✓ Concomitant diseases, concomitant treatments/medications
- ✓ Physical Examination
- ✓ Vital signs
- ✓ PSQI scale score (estimated time 20 minutes)
- ✓ Seattle Angina Scale score (estimated time 20 minutes)
- ✓ Sleeping medication use
- ✓ Electrocardiogram

Baseline: Visit 2 (Week 0, day of randomization) requires you to return to the study center on an empty stomach and complete the following:
- ✓ Entry Criteria
- ✓ Concomitant diseases, concomitant treatments/medications
- ✓ Physical Examination
- ✓ Vital signs
- ✓ PSQI scale score (estimated time 20 minutes)

- ✓ Seattle Angina Scale score (estimated time 15 minutes)
- ✓ Hamilton Depression Scale (HAMD) (estimated time 15 minutes)
- ✓ Generalized Anxiety Disorder Scale (GAD-7) (estimated time 15 minutes)
- ✓ Health-Related Quality of Life Questionnaire (SF-36) (estimated time 15 minutes)
- ✓ Sleeping medication use
- ✓ Electrocardiogram
- ✓ Randomization
- ✓ Needle site observation
- ✓ Laboratory tests (routine blood, blood biochemistry, CRP, C-reactive protein; Lp-PLA2, lipoprotein-associated phospholipase A2; C-FABP, cardiac fatty acid-binding protein level;)

Treatment Visits: visits 3 and 4 (week 4 ± 2 days and week 8 ± 2 days of treatment) are on-site visits conducted at the study center and require you to complete the following:

- ✓ Concomitant Diseases, Concomitant Treatments/Medications
- ✓ Physical examination
- ✓ Vital signs
- ✓ PSQI scale score (estimated time 20 minutes)
- ✓ Seattle Angina Scale score (estimated time 15 minutes)
- ✓ Hamilton Depression Scale (HAMD) (estimated time 15 minutes)
- ✓ Generalized Anxiety Disorder Scale (GAD-7) (estimated time 15 minutes)
- ✓ Health-Related Quality of Life Questionnaire (SF-36) (estimated time 15 minutes)
- ✓ Sleeping medication use
- ✓ Electrocardiogram
- ✓ Needle site observation
- ✓ Adverse event assessment

Treatment Visits: Visit 5 (Week 12 ± 2 days of treatment) requires you to return to the study center on an empty stomach and complete the following:

- ✓ concomitant diseases, concomitant treatments/medications
- ✓ Physical Examination
- ✓ Vital signs
- ✓ PSQI scale score (estimated time 20 minutes)
- ✓ Seattle Angina Scale score (estimated time 15 minutes)
- ✓ Hamilton Depression Scale (HAMD) (estimated time 15 minutes)
- ✓ Generalized Anxiety Disorder Scale (GAD-7) (estimated time 15 minutes)
- ✓ Health-Related Quality of Life Questionnaire (SF-36) (estimated time 15 minutes)
- ✓ Sleeping medication use
- ✓ Electrocardiogram
- ✓ Needle site observation

- ✓ Laboratory tests (routine blood, blood biochemistry, CRP, C-reactive protein; Lp-PLA2, lipoprotein-associated phospholipase A2; C-FABP, cardiac fatty acid-binding protein level;)
- ✓ Adverse event assessment

Safety Follow-Up: Visits 6 and 7 (Week 16 ± 2 days, Week 24 ± 2 days) are on-site visits conducted at the study center, and will also be required to complete the following if you leave the study early:

- ✓ Concomitant Diseases, Concomitant Treatments/Medications
- ✓ Physical examination
- ✓ Vital signs
- ✓ PSQI scale score (estimated time 20 minutes)
- ✓ Seattle Angina Scale score (estimated time 15 minutes)
- ✓ Hamilton Depression Scale (HAMD) (estimated time 15 minutes)
- ✓ Generalized Anxiety Disorder Scale (GAD-7) (estimated time 15 minutes)
- ✓ Health-Related Quality of Life Questionnaire (SF-36) (estimated time 15 minutes)
- ✓ Sleeping medication use
- ✓ Electrocardiogram
- ✓ Needle site observation
- ✓ Adverse event assessment

Unscheduled Visits (if applicable)

Additional study visits may be conducted at any time if deemed necessary by the investigator or by you and/or the legal representative. All information (including the reason for the visit, any information about the AE, etc.) should be collected in the original document and recorded in the appropriate section of the eCRF.

6 Your obligations

You must furnish a replica of your identification documentation, a replica of your bank card, particulars regarding your recent outpatient visits, and the cognomen, dosage, and administration mode of medicaments ingested orally for insomnia therapy hitherto.

You must adhere to the principal investigator's arrangements to undergo treatment and associated examinations, thereby enabling assessment and monitoring of your physical status and therapeutic efficacy, prompting timely program modifications and meticulous observation of your affliction.

You must apprise your study physician regarding all current and forthcoming medicaments and therapies, abstaining from regimen alterations or inaugurating novel

treatments sans consultation.

You must notify your study physician of any health issues, irrespective of perceived insignificance. You shall also disclose all historical and contemporary medicinal agents, encompassing herbal remedies.

You shall be compelled to fast nocturnally for over 10 hours antecedent to each blood biochemistry examination.

You shall be directed to revisit the research center at the specified time for an “early termination” assessment if discharged prematurely for any rationale.

You will be mandated to undergo routine check-ups for your safety.

You are stringently forbidden to participate in any other investigative treatments concurrently with this study.

7 Possible adverse effects or risks

The acupoints selected for the study have been utilized in manifold trials sans documentation of severe adverse events. Mild tingling may transpire during needling. The most prevalent sensations post-needling embody numbness, soreness, or dullness circumscribing the needle locus. If you experience the aforementioned or any other unease during the study, or suspect an adverse reaction, promptly notify your study physician, who shall render an assessment and furnish appropriate therapies for your circumstances.

The study physician shall monitor any adverse reactions arising during the study. Moreover, study procedures confer inherent risks and discomforts. As an element of informed consent, you shall be apprised of the hazards associated with all study protocols. We shall implement measures to minimize the effects and discomfort of said procedures.

Blood Drawing

Blood draws may elicit discomfort, bleeding, or bruising at the puncture locus. Diminutive scabs or tumefaction may materialize locally. Infrequently, syncope or localized infection may betide. Notify the study physician or research personnel if you experience unease during phlebotomy.

Electrocardiogram

The ECG electrode site may necessitate cleansing, occasionally mandating skin preparation. In rare instances, localized rash or irritation may arise. Otherwise, the examination is customarily risk-free.

8 Anticipated Benefits

If the study treatment proves efficacious, it harbors the potential to decelerate disease progression and ameliorate symptomatology. However, such benefits cannot be guaranteed.

Throughout enrollment, you shall garner scientific guidance regarding your affliction alongside meticulous medical monitoring from the study physician. Your contribution shall advantage patients with analogous diseases, for which we are tremendously appreciative!

9 Other Optional Medications or Therapies

Enrollment is fully voluntary and not requisite for receiving treatment. Refusal to participate shall not impact your access to conventional medical care or precipitate any discrimination. Your study physician shall discuss additional available treatments or pharmaceuticals alongside their respective risks and benefits.

10 Is study participation obligatory and complete enrollment requisite?

Your involvement is entirely voluntary. You may decline to participate sans any negative ramifications for current or prospective healthcare. Likewise, you may withdraw at any juncture without rationale, again sans impact upon conventional medical access. If electing to terminate enrollment, please promptly notify your study physician to enable counsel regarding your health status. For withdrawn participants, we implement telephonic safety follow-ups, which you may decline. We may recontact you if emergent health or rights-related intelligence surfaces post-withdrawal.

11 Compensation for research-related injuries

Please immediately report any unease, dilemmas, or study-related injuries to your

study physician during enrollment. The sponsor has secured insurance for this trial. The sponsor shall assume treatment expenditures and commensurate pecuniary indemnification pursuant to Chinese regulations for any direct therapeutic or procedural adverse reactions sustained herein. Injuries attributable to medical malpractice or protocol noncompliance shall not qualify for sponsorship.

12 Enrollment-related fees and remunerations

The sponsor shall assume costs for study-related examinations (encompassing routine bloodwork, urinalysis, diagnostics, electrocardiography, vital sign measurement, physical examination, quantitative tests, anthropometrics, etc.) and acupuncture treatments herein. Participation shall furnish comprehensive cognition of your physical wellbeing.

Expenses for concurrent comorbidities shall not qualify for complementary coverage. You shall not receive direct remuneration for enrollment. However, you may incur transportation outlays to the research center, for which reasonable reimbursement shall be furnished. Specifically, each on-site visit shall confer a $200 transportation subsidy. For incomplete participation, disbursement shall correspond to the quantity of finished visits.

13 Voluntary enrollment

Your participation is entirely voluntary, and you may decline or withdraw at any point without prejudice or retaliation. Your medical care and rights shall not be impacted.

14 Confidentiality Principles

The study shall implement appropriate safeguards to protect your privacy and personal data pursuant to Chinese regulations on personal information security. Any acquired intelligence shall remain confidential. Your records will be identified by your study subject number per local mandates. These records shall be securely maintained at the research center.

The sponsor and its agents, monitors, inspectors, government bodies, health authorities, ethics committees, and institutional review boards reserve the right to review your medical records at the research center or investigating physician’s office alongside examining clinical research procedures and data without compromising confidentiality.

By endorsing this informed consent form, you authorize the processing and

application of your personal information for this research study.

Data Collected:

Personal health data derived from original medical records alongside all participation-generated data shall be acquired during the study. Your personal health information shall encompass physical examination findings, various blood test results, and other medical diagnostic results.

Data Presentation:

Unless legally mandated otherwise, your identifiable and contact information shall remain confidential via usage of codes (e.g., numbers) and initials (if permitted locally and by the sponsor) in lieu of actual names.

Why this data is collected:

Your personal health information shall be utilized for clinical research. Deidentified data may appear in research reports or scientific presentations. Your personal health data shall remain confidential and not be disclosed unless legally required. Coded information may enable follow-up research post-study completion.

Who will have access to your data:

The sole parties privy to your identifiable personal health data include the study physician, assisting center staff, and study members sworn to confidentiality. Your file shall be secured within a locked cabinet, solely accessible by study personnel. Government bodies and the Ethics Committee may require data access per research unit mandates. No identifying information shall be published alongside study results.

Entitlements Pertaining to Your Data:

You are entitled to procure, amend, and duplicate medical and/or clinical research documentation in accordance with prevailing privacy legislations.
Such records can be requisitioned for examination from the presiding research physician or the institution overseeing the investigation.
Nevertheless, to preserve the integrity of the research findings, you must acquiesce to the stipulation that your research-affiliated records remain inaccessible and non-duplicable until the culmination of the study.
Nevertheless, to preserve the integrity of the research findings, you must acquiesce to the stipulation that your research-affiliated records remain inaccessible and non-duplicable until the culmination of the study.
Disposal of Samples.

Any remaining blood samples belonging to you (voluntarily provided) may be retained for research purposes for several years after the study is completed. Should the sponsor envisage any novel assessments on the specimen that do not pertain to this research, your explicit permission shall be sought: you will be prompted to affix your signature to an updated informed consent document to sanction further utilization of your sample. You retain the prerogative to decline.

15 Circumstances in which you may be terminated from the trial

The study physician may terminate your participation sans consent if sustained enrollment is deemed detrimental. If the study ceases, the study physician shall notify and aid you in obtaining alternate treatment externally.
When discharged for any rationale, you shall be directed to return for final evaluation by the study physician, potentially encompassing examination and diagnostics.

16 Study Enrollment Volume

Approximately 110 patients with perimenopausal angina and insomnia shall participate herein.

17 How taking part in this study will affect your daily life？

When determining whether participation in this study is appropriate, please carefully consider the potential impact the aforementioned tests and follow-up visits may have on your daily work, family life, et cetera. Contemplate the timing and transportation required for each follow-up visit. If you have any inquiries regarding the tests and procedures involved in the trial, you may solicit advice from the study physician.

Should you require any medication during the study, please consult with the study physician in advance. For your safety and to ensure validity of study results, you will be unable to participate in any other clinical trials investigating pharmaceuticals or medical devices concurrently.

18 Who do you contact if you have questions?

If you have questions about any part of this study, you may ask the study doctor any questions about this study at any time and they will try to help you resolve the problem.

The contact information for the study doctor is listed below:
Name: [Rui Shi]
Phone: [0431-86177913]
Name: [Yue Deng]
Phone: [0431-86177343].

If you feel that your rights and interests have been violated during the study and you do not wish to discuss the matter with the study doctor or the study team, you may contact the following designated unit: [Ethics Committee of the Affiliated Hospital of Changchun University of Traditional Chinese Medicine]
Tel: [0431-86177044].

We appreciate you taking the time to review this information and contemplating participation in this study.

Informed Consent Form - Signature Page

Participant Informed Consent Statement:

I have elucidated to the participant the context, objectives, methodologies, potential hazards, and prospective advantages of the investigation titled " Randomized, double-blind, placebo-controlled study of the efficacy and safety of acupuncture for perimenopausal stable angina pectoris with insomnia " I have accorded him/her ample opportunity to peruse the informed consent document, engage in discussions with others, and have satisfactorily addressed any queries he/she might have pertaining to the study. I have apprised the said participant of the appropriate contact avenues should any complications or concerns arise. Additionally, I have conveyed to this individual that he/she retains the prerogative to recuse themselves from this investigation at any juncture, devoid of necessitating a justification.

Name of Participant: _______________ Phone number: ____________________
Signature: ________________ Date: _______________
Guardian's name (if applicable): __________ Phone number: _________
Signature of Guardian : ________________ Date: _______________

Research Physician/Authorized Personnel Statement:

I have elucidated to the participant the context, objectives, methodologies, potential hazards, and prospective advantages of the investigation titled "Randomized, double-blind, placebo-controlled study of the efficacy and safety of acupuncture for perimenopausal stable angina pectoris with insomnia "

I have accorded him/her ample opportunity to peruse the informed consent document, engage in discussions with others, and have satisfactorily addressed any queries he/she might have pertaining to the study.I have apprised the said participant of the appropriate contact avenues should any complications or concerns arise.

Additionally, I have conveyed to this individual that he/she retains the prerogative to recuse themselves from this investigation at any juncture, devoid of necessitating a justification.

Name of investigating physician/authorized person: _________________________
Phone number: ______________________
Signature of Study Physician/Authorized Person: __________Date: ____________
