# Supplementary material for: Exploring acupuncture as a treatment for insomnia in perimenopausal women with stable angina pectoris: A protocol for a randomized, double-blind, placebo-controlled clinical trial
Source: PLoS One. 2024 Apr 18;19(4):e0301827. doi: 10.1371/journal.pone.0301827 (PMC11025937; doi:10.1371/journal.pone.0301827)

Acupuncture for perimenopausal stable angina with insomnia: a randomized, double-blind, placebo-controlled clinical trial protocol

Program No. : CCZYFYLL2022-029

Sponsor : The Affiliated Hospital of Changchun University of Traditional Chinese Medicine

Principal Investigator: Yue Deng, Rui Shi

Version number: V 2.0

Version date: August 26, 2022

Researcher's Statement and Signature Page

Investigator Statement:

I will conscientiously fulfill my duties as an investigator in accordance with GCP regulations.

I have received the investigator's brochure, I am aware of the investigational procedures for this investigational drug, and I have been informed that I will receive an updated investigator's brochure in a timely manner.

I have read this protocol and this study will be conducted in accordance with the moral, ethical and scientific principles set forth in the Declaration of Helsinki and the Chinese GCP. I agree to conduct this clinical study in accordance with the design and provisions of this protocol.

I will be responsible for making clinically relevant medical decisions to ensure that subjects receive timely and appropriate treatment in the event of adverse events during the study, I am aware of the requirements for the proper reporting of serious adverse events, and I will record and report these events as required.

I will ensure that the data are true, accurate, complete, timely, and legally contained in the case report form. I will accept audits and inspections by monitors or inspectors dispatched by the sponsor and by the drug regulatory authorities to ensure the quality of the clinical study.

I agree that the results of the study will be used for drug registration and published publicly.

I will provide a curriculum vitae to be presented to the Ethics Committee and possibly to the regulatory authorities for review prior to the start of the study.

Signature of the principal investigator of each clinical research center:

Full name of research center: The Affiliated Hospital of Changchun University of Traditional Chinese Medicine

City: Changchun, Jilin Province

Name of Principal Investigator: Shi Rui, Deng Yue, Meng Wenyi, Liu Zhaozheng, Xue Wen, Chen Xingyu August 26, 2022

Table of Contents

Summary of the program 6

Test Flowchart 9

1. Background of the study 10

2. Purpose of the study 11

3. Experimental design 11

3.1 Overall Design 11

3.2 Randomization Procedure and Allocation Hidden 11

3.3 Controls 11

3.4 Sample Size Calculation 12

3.5 Blinding 12

3.6 Emergency blinding 12

3.7 Design of the treatment program 12

4. Trial subjects 13

4.1 Diagnostic criteria 13

4.2 Inclusion criteria 13

4.3 Exclusion Criteria 13

4.4 Withdrawal and termination criteria 14

5. Interventions 15

5.1 Treatment group 15

5.2 Control group 15

5.3 Permitted treatments 15

6. Study Process and Visit Evaluation 16

6.1 Visit 1 - Screening 17

6.2 Baseline: Visit 2 17

6.3 Treatment visit: visit 3, 4 17

6.4 Treatment visit: visit 5 18

6.5 Safety follow-up visits: visits 6, 7 19

6.6 Unplanned visits 19
6.7 Early withdrawal 20
6.8 Precautions during the trial 20
7 Criteria for evaluation of efficacy and safety 20
7.1 Primary outcome 20
7.2 Secondary outcomes 20
7.3 Indicators of safety 21
7.4 Safety evaluation 21
7.4.1 Definitions and regulations related to adverse events 21
7.4.2 Record of adverse events 22
7.4.3 Criteria for Severity Determination 22
7.4.4 Adverse events and drug causal relationship judgment 22
7.4.5 Adverse event detection, tracking and follow-up 23
7.4.6 Reporting procedures and treatment 23
7.4.7 Follow-up of unrelieved adverse events 24
8. Risk Management and Risk Assessment 25
9. Quality control and quality assurance 25
10. Data Management and Verification 26
10.1 Data Management Plan 26
10.2 Data Verification, Cleaning, and Quality Control 26
10.3 Data Locking and Withdrawal 27
11. Statistical Analysis 27
12. Ethics and Subject Protection 28
12.1 Ethical Guidelines 28
12.2 Ethics Committee 28
12.3 Informed Consent Process 28
12.4 Medical Treatment and Protection of Subjects 30
12.5 Protection of Subjects' Privacy 30
12.6 Recruitment of Subjects 31

13. Quality Assurance and Quality Control 31
13.1 Quality Control of Subjective Symptom Evaluation 31
13.2 Quality Control of Laboratory Test Indicators 31
13.3 Quality Control of Clinical Surveillance/Audit 31
14. Data Retention 32
15.References 32
Appendix 1 Pittsburgh Sleep Quality Index 35
Appendix 2 SF-36 Quality of Life Scale Questionnaire 37
Appendix 3 Hamilton Depression Scale (HAMD) 41
Appendix 4 Seattle Angina Scale 45
Appendix 5 Generalized Anxiety Scale (GAD-7) 48
Appendix 6 Allocation of Acupoints and Non acupoints 50
Appendix 7 Schematic of the two groups of acupuncture and non-penetrating devices. 51

Summary of the program

Trial Title	Acupuncture for perimenopausal stable angina with insomnia: a randomized, double-blind, placebo-controlled trial
applicant organization	Applicant Hospital of Changchun University of Traditional Chinese Medicine
Trial Objective	To assess the efficacy and safety of acupuncture in treating perimenopausal subjects with stable angina and insomnia.
Target number of cases	55 cases in each of the treatment and control groups, totaling 110 cases
Test Grouping	Treatment group: manual acupuncture<br>Control group: sham acupuncture group
Experimental Designs	Single-center, randomized, double-blind, placebo-controlled clinical trial design.
Sample size\Statistical design	The Pittsburgh Sleep Quality Index (PSQI) score following the full acupuncture intervention regimen was utilized to determine the requisite sample size. Statistical significance was defined as α=0.05 with a power of 90%. Based on a prior study, perimenopausal women with insomnia had mean±SD PSQI scores of 11.43±2.11 with sham acupuncture and 9.20±3.24 with manual acupuncture. Using the sample size formula: $n = 2 \times \left[\frac{(Z_\alpha + Z_\beta) \times \sigma}{\delta}\right]^2$ Using the larger standard deviation of 3.24, with δ = 11.43 - 9.20 = 2.23, β = 0.1, 1-β = 0.9, and α = 0.05. The resultant sample size required per group was 44. Allowing for a 20% loss to follow-up, the total sample size needed for the study was 110 participants.

object of study	Inclusion Criteria<br>1. Meet diagnostic criteria for both stable angina pectoris and insomnia as delineated above;<br>2. Female gender, 45-55 years of age, in the perimenopausal period;<br>3. No acupuncture treatment for minimum 6 months prior to enrollment;<br>4. Provide informed consent and agree to randomized group allocation.<br>Exclusion Criteria<br>1. Not in perimenopausal phase;<br>2. Severe renal, hepatic, hematologic or other systemic diseases;<br>3. Psychiatric disorders or impaired consciousness;<br>4. Uncontrolled severe hypertension, diabetes mellitus or malignant arrhythmia;<br>5. valvular disease, Recent acute myocardial infarction, unstable angina, myocarditis, hypertrophic cardiomyopathy, or post-pacemaker severe heart failure (NYHA grade ≥III);<br>6. Major surgery, trauma, bleeding event or severe infection within past 3 months;<br>7. Insomnia attributable to medications, alcohol or environmental factors;<br>8. Local skin infections near acupoint sites;<br>9. Malignancy or hyperthyroidism;<br>10. History of syncope or severe needle phobia;<br>11. Any other condition potentially interfering with study participation or completion as deemed by the investigator.
intervention	Participants will receive 36 acupuncture treatments over a 12-week period at a frequency of three times per week.
research period	Primary and secondary outcomes will be assessed at baseline, 4 weeks, 8 weeks, 12 weeks (after completion of the intervention), and at an additional 12-week follow-up.
Suspension/withdrawal criteria	When a serious safety problem occurs in the trial, to protect the rights and interests of the subjects, or for financial reasons, management reasons, etc., the investigator, the ethics committee, the sponsor, and the health administration may carry out the termination of the trial. Subjects who are unwilling or unlikely to continue the clinical trial propose to the investigator to withdraw from the trial. Any adverse event during the course of the study; comorbidities, complications, or physiologic changes that prevent continued participation; poor treatment adherence; contraindications to intervention; loss of diagnosis;

	unmasking events.
physical and chemical examination	Includes c-reactive protein (CRP), lipoprotein-associated phospholipase A2 (Lp-PLA2), cardiac fatty acid-binding protein level (C-FABP), blood counts (WBC, RBC, HGB, PLT), urinary counts, liver function (ALT, AST), renal function (Scr, BUN), and twelve-lead electrocardiogram.
clinical examination	1.body temperature;2. heart rate at quiet; 3. respiration; 4. blood pressure.
Evaluation of effectiveness	Primary outcomes：<br>Pittsburgh Sleep Quality Index (PSQI)<br>Secondary Outcomes：<br>1.Assessment of stable angina pectoris (SAP):<br>2.Quality of life assessment using the 36-Item Short Form Health Survey (SF-36)<br>3. Dosage of sleeping pills.<br>4. Hamilton Depression Rating Scale (HAMD).<br>5. Generalized Anxiety Disorder 7-item (GAD-7) scale.
security assessment	1.Vital signs (temperature, blood pressure, respiratory rate, heart rate);<br>2.Routine blood and urine tests;<br>3.Liver and kidney function tests;<br>4.Electrocardiogram.
statistical analysis	Statistical analyses will be conducted using SPSS 24.0. Continuous variables will be summarized as mean ± standard deviation or median (interquartile range). Between-group comparisons will utilize two-sample t-tests or non-parametric tests for skewed data. Within-group baseline to endpoint changes will be analyzed by paired t-tests. Categorical variables will be compared using Fisher's exact or Chi-square tests. Statistical significance will be defined as $p<0.05$.

Test Flowchart

	STUDY PERIOD						
	Enrolment	Allocation	Treatment period			Follow-up period	
**TIMEPOINT (week)**	-1	0	4	8	12	16	24
**ENROLLMENT:**	X						
**Eligibility screen**	X						
**Informed consent**		X					
**Allocation**		X					
**Demographics**		X					
**Vital signs**		X	X	X	X	X	X
**INTERVENTIONS:**							
**Manual cupuncture**			◆———	———	———◆		
**Sham acupuncture**			◆———	———	———◆		
**ASSESSMENTS:**							
**PSQI**		X	X	X	X	X	X
**CRP**		X			X		
**Lp-PLA2**		X			X		
**C-FABP**		X			X		
**SAQ**		X	X	X	X	X	X
**SF-36**		X	X	X	X	X	X
**Dosage of sleeping pills**		X	X	X	X	X	X
**HAMD**		X	X	X	X	X	X
**GAD-7**		X	X	X	X	X	X
**Electrocardiography**		X	X	X	X		X
**Laboratory examination**		X			X		
**Adverse events**			X	X	X	X	X

1.Background of the study

Stable angina pectoris (SAP), as a prevalent manifestation of coronary artery disease, exerts a pernicious impact on patients' quality of life and portends a marked risk of progression to acute coronary syndromes [1-3]. In recent years, insomnia comorbid with SAP has garnered greater attention, with severe sleep disturbances emerging in numerous coronary artery disease patients [4,5]. Given the precipitous decline in estrogen levels, perimenopausal women exhibit heightened vulnerability to coronary artery disease and insomnia [6,7].

The occurrence of angina pectoris may engender insomnia, which can adversely impact emotional regulation, cognition, memory, and systemic immunity, whilst also inflicting multi-organ damage including the cardiovascular system [8,9].

In an 11-year longitudinal study, Sivertsen et al. identified insomnia as a salient risk factor for angina pectoris, cerebrovascular accident, and hypertension [10]. While sleep medicine research commenced in China in the 1980s, a formalized discipline remains nascent, and lay awareness of sleep hygiene remains negligible, with approximately 43% of Chinese citizens exhibiting varying degrees of sleep pathology, disproportionately afflicting women [11, 12]. The surging medical expenditures and diminished work productivity engendered by sleep disorders have emerged as pressing public health concerns [13].

Traditional physiotherapeutic modalities, behavioral therapeutic strategies, and psychotherapeutic interventions necessitate a manifold array of specialized personnel and material accoutrements, entailing an intricate interplay of multifarious determinants, encompassing even the specter of perilous remedial sequelae. Such comprehensive paradigms encounter formidable hurdles in their quest for pervasive adoption within clinical milieus. Pharmacological intervention, a modality ubiquitously resorted to, casts a prominent shadow within the therapeutic landscape. Among these, sedative agents emerge as the veritable cornerstone of clinical practice. Nonetheless, the protraction of drug administration over prolonged epochs exposes patients to the vicissitudes of drug reliance, counteractive resurgences, compulsive enthrallment, alongside a sundry constellation of untoward sequelae, underscored by the ominous propensity for withdrawal manifestations upon cessation [14].

In recent years, the clinical efficacy of acupuncture has garnered escalating recognition, with multiple studies elucidating its therapeutic utility for insomnia [15-17].Acupuncture can enhance sleep quality by facilitating slow-wave sleep [18,19], and it is also efficacious in the management of perimenopausal insomnia [20-23].

Recent investigations indicate a robust association between pneumonia induced by the 2019 novel coronavirus (2019-nCoV) and an elevated predisposition to angina and insomnia [24,25].While pertinent assessments of acupuncture's efficacy in treating insomnia have been undertaken in the past, the onset of the 2019-nCoV pandemic has resulted in a significant paucity of current analyses of randomized controlled trials, especially concerning the specialized indication of concurrent angina and insomnia in perimenopausal women. In light of the paucity of data evaluating the efficacy and safety of acupuncture for insomnia specifically in perimenopausal patients with stable angina, we undertook this study.

2. Purpose of the study

The primary purpose of this research protocol is to assess the effectiveness and safety of acupuncture in perimenopausal subjects with both stable angina and insomnia.

3. Experimental design

3.1 Overall design

This trial is a single-center, randomized, double-blind, placebo-controlled clinical trial.

3.2 Randomization procedure and allocation concealment

Randomization numbers will be generated independently by the study coordinator without contacting study participants using SPSS 24.0 software. To ensure that allocation is concealed, sequentially numbered opaque sealed envelopes will be used and the randomization sequence will be managed independently by a designated person.

The format of the randomization number in the randomization table will be R + three Arabic numerals, written sequentially from R001. Successfully screened subjects will be assigned a randomization number in ascending order of the screening number, and the trial treatment corresponding to the number will be used during the course of the trial. A randomized subject who withdraws from the clinical trial during the course of the trial for any reason, regardless of whether hand-acupuncture treatment or placebo was used, will retain his or her randomization number, and the subject will not be allowed to re-enter the trial and will not be substituted.

3.3 Control

A placebo (sham acupuncture group) control will be used.

3.4 Sample size calculation

The Pittsburgh Sleep Quality Index (PSQI) score following the full acupuncture intervention regimen was utilized to determine the requisite sample size. Statistical significance was defined as α=0.05 with a power of 90%. Based on a prior study, perimenopausal women with insomnia had mean±SD PSQI scores of 11.43±2.11 with sham acupuncture and 9.20±3.24 with manual acupuncture. Using the sample size formula:

$$n = 2 \times \left[\frac{(Z_{\alpha} + Z_{\beta}) \times \sigma}{\delta} \right]^2$$

Using the larger standard deviation of 3.24, with δ = 11.43 - 9.20 = 2.23, β = 0.1, 1-β = 0.9, and α = 0.05. The resultant sample size required per group was 44. Allowing for a 20% loss to follow-up, the total sample size needed for the study was 110 participants.

3.5 Blinding

Group assignments will be concealed from patients, assessors and data analysts. Acupuncture points and techniques will be comparable between the two groups. The control group will be blinded by performing sham acupuncture using a non-piercing device that mimics the appearance of real needles. To assess the success of the blinding, we will investigate the participants' perceptions of whether they received real or sham acupuncture after the intervention. Due to the unique nature of acupuncture, it is not feasible to blind acupuncturists.

3.6 Emergency blinding

Blinding may be broken in emergency situations when the investigator believes that knowledge of the treatment received by the participant is beneficial for the management of adverse events. In the case of emergency blinding, the emergency blinding operation will be performed by a person authorized for the study, and the reason, time, and place of the blinding will be documented in detail and signed in the subject's study medical record, eCRF, and any corresponding AE report (and should be accompanied by the signature of at least one other witness in addition to the investigator in charge), and the investigator must notify the sponsoring organization within 24 hours of the emergency blinding, with prompt notification to the The Principal Investigator and Clinical Supervisor must be notified promptly. After the emergency blinding, the investigational drug should be discontinued, and the subject should be discontinued from the trial and treated as a dropout case.

3.7 Design of the treatment program

① The purpose of designing the introductory period is to eliminate the delayed effects of having taken a similar treatment, stabilize the baseline level, remove the placebo effect as well as to adequately determine the inclusion and exclusion criteria and to develop subject compliance. A 7-day introductory period was set up for this trial.

② Design of the Treatment Period

The treatment period of this study Total 12 weeks

③Design of the follow-up period

The follow-up period of this study totaled 12 weeks.

4. Test subjects

4.1 Diagnostic criteria

① The diagnosis of stable angina follows the ESC Guidelines for the Diagnosis and Treatment of Chronic Coronary Syndromes 2019. Coronary artery disease was determined by coronary angiography, CT angiography or a positive treadmill exercise test. Patients presented with Canadian Cardiovascular Society class II-III angina, a history of stable angina for ≥30 days, and 2-5 angina episodes per week.

② Insomnia was defined according to the International Classification of Sleep Disorders as a Pittsburgh Sleep Quality Index greater than 5. Questionnaires on insomnia duration and frequency were administered by two independent blinded assessors at the time of admission, and consensus analysis was performed to ensure consistency of results. All enrolled patients had a self-reported insomnia duration of at least 30 days and ≥3 episodes of insomnia per week.

4.2 Inclusion criteria

① Meet the above diagnostic criteria for stable angina and insomnia;

② Female, 45-55 years old, in the perimenopausal period;

③ No acupuncture treatment for at least 6 months prior to enrollment;

④ Provide informed consent and agree to be randomized.

4.3 Exclusion Criteria

① Not in perimenopause;

② Severe renal, hepatic, hematologic or other systemic diseases;

③ Mental disorder or consciousness disorder;

④ Uncontrolled severe hypertension, diabetes mellitus or malignant arrhythmia;

⑤ Valvular disease, recent acute myocardial infarction, unstable angina pectoris, myocarditis, hypertrophic cardiomyopathy, or severe heart failure after pacemaker surgery (NYHA classification ≥ III);

⑥Major surgery, trauma, bleeding event, or serious infection within the past 3 months;

⑦Insomnia due to drugs, alcohol or environmental factors;

⑧ Localized skin infection in the vicinity of the acupuncture point;

⑨ malignant tumors or hyperthyroidism

⑩ history of syncope or severe needle phobia;

⑪ any other condition that, in the opinion of the investigator, may interfere with participation or completion of the study.

4.4 Criteria for withdrawal and termination of the trial

Subjects have the right to withdraw from the study at any time for any reason, in accordance with the ethical requirement to safeguard patient autonomy. For subjects who choose to withdraw, their reasons for withdrawal must be recorded in detail and used as an assessment indicator for stopping the trial. Clinical investigators should carefully document the basis for termination of the trial and its relevance to the study, and analyze the potential impact of endpoint events on the study conclusions.

All primary data on study withdrawals and terminations will remain sealed for inspection after completion of the trial. In addition, grounds for termination of a trial include: any adverse event during the study; comorbidities, complications, or physiologic changes that preclude continued participation; poor treatment adherence; contraindicated interventions; missed diagnoses; and release from restraint events.

5. Interventions

Participants will receive 36 acupuncture treatments over a 12-week period at a frequency of 3 times per week. Acupuncture can only be performed by licensed acupuncturists. They must be trained in standardized techniques, including needle manipulation and point localization.

5.1 Treatment group

Sterile acupuncture needles with a diameter of 0.30 mm and a length of 40 mm were used (Suzhou Warren Medical Equipment Co., Ltd.). The identification and localization of acupuncture points were in accordance with the standards of the World Health Organization. The prescribed acupoints were PC6 (Neiguan), EX-HN3 (Yintang), KI6 (Zhaohai), RN17 (Tanzhong), HT3 (Shaohai), and SP6 (Sanyinjiao).The insertion depths of EX-HN3 and RN17 were 5-10 mm, whereas the insertion depths of KI6, PC6, HT3, and SP6 were 20-25 mm. The needling procedure consisted of sterilizing the skin and instruments; inserting needles at the acupoints and rotating them bi-directionally between 90°-180° at a speed of 60-90 revolutions/minute until deqi sensations of numbness, distension, soreness, and pain appeared, which were quantified using the Chinese Modified Massachusetts General Hospital Acupuncture Sensation Scale (C-MASS). Each acupuncture session lasted 30 minutes, with manual stimulation for 1 minute every 10 minutes.

5.2 Control Group

In the sham acupuncture group, after the same sterilization procedure as that of the treatment group, non-penetrating needling was performed on non-acupoints (the selected stimulation points were close to the acupoints of the treatment group but outside the meridians, avoiding the distribution of the body's nerves) using blunt-tipped needles with a diameter of 0.30 mm and a length of 40 mm (Warren Medical Devices Co., Ltd., Suzhou, China). The specific non-acupuncture point selection is shown in Appendix 6. The treatment time was the same as that of the treatment group, except that the blunt needles (shown in Appendix 7)were only lightly tapped on the surface of the skin and did not penetrate the tissue or manipulate it to stimulate outflow of qi. Dummy needle stimulation was simulated for 1 minute every 10 minutes during the 30-minute treatment.

5.3 Permitted treatment

①Drugs that have been used by the subject prior to enrollment in the trial for the treatment of co-morbidities may continue to be used, and the original drug type and dosage may remain unchanged as

much as possible for the duration of the trial.

②Subjects were allowed to take uniformly distributed nitroglycerin by mouth during angina attacks, and detailed records were kept.

③If an adverse event occurs during the trial, the subject may be treated with the appropriate medication according to the relevant guidelines, but the investigator should assess whether the medication used has an impact on the efficacy or safety evaluation of the trial medication and, if appropriate, determine whether the subject needs to be withdrawn from the trial after receiving the appropriate treatment.

④Medicines or other treatments taken during the trial need to be used under the direction of the investigator. Any medication used during the trial must be recorded and described in detail in the study medical record, including the name of the medication, method of use, dosage of the medication, and the reason for its use, so that it can be analyzed and reported at the time of the summary.

6 Research Process and Visit Evaluation

6.1 Visit 1-Screening (-7 to 0 days)

- ✓ Signed informed consent
- ✓ entry standard
- ✓ population studies
- ✓ (a person's) age
- ✓ distinguishing between the sexes
- ✓ ethnic group
- ✓ (a person's) height
- ✓ weight
- ✓ Past medical history and comorbidities
- ✓ Concomitant Diseases, Concomitant Treatments/Medications
- ✓ clinical examination

- ✓ vital signs
- ✓ PSQI scale scores
- ✓ Seattle Angina Scale Score
- ✓ Use of sleeping medication
- ✓ electrocardiography

6.2 Baseline: visit 2 (week 0, day of randomization)

- ✓ entry standard
- ✓ Concomitant Diseases, Concomitant Treatments/Medications
- ✓ clinical examination
- ✓ vital signs
- ✓ PSQI scale scores
- ✓ Seattle Angina Scale Score
- ✓ Hamilton Depression Scale (HAMD)
- ✓ Generalized Anxiety Disorder Scale (GAD-7)
- ✓ Health-related Quality of Life Questionnaire (SF-36)
- ✓ Use of sleeping medication
- ✓ electrocardiography
- ✓ randomization
- ✓ Needle site observation
- ✓ Laboratory tests (routine blood, blood biochemistry, CRP, C-reactive protein; Lp-PLA2, lipoprotein-associated phospholipase A2; C-FABP, cardiac fatty acid-binding protein level;)

6.3 Treatment visits: visits 3 and 4 (week 4 ± 2 days and week 8 ± 2 days of treatment)

- ✓ Concomitant Diseases, Concomitant Treatments/Medications
- ✓ clinical examination
- ✓ vital signs
- ✓ PSQI scale scores
- ✓ Seattle Angina Scale Score
- ✓ Hamilton Depression Scale (HAMD)
- ✓ Generalized Anxiety Disorder Scale (GAD-7)
- ✓ Health-related Quality of Life Questionnaire (SF-36)
- ✓ Use of sleeping medication
- ✓ electrocardiography
- ✓ Needle site observation
- ✓ Adverse event assessment

6.4 Treatment visits: visit 5 (week 12 ± 2 days of treatment)

- ✓ Concomitant Diseases, Concomitant Treatments/Medications
- ✓ clinical examination
- ✓ vital signs
- ✓ PSQI scale scores (estimated time 20 minutes)
- ✓ Seattle Angina Scale score (estimated time 15 minutes)
- ✓ Hamilton Depression Scale (HAMD) (estimated time 15 minutes)
- ✓ Generalized Anxiety Disorder Scale (GAD-7) (estimated time 15 minutes)
- ✓ Health-related quality of life questionnaire (SF-36) (estimated time 15 minutes)

- ✓ Use of sleeping medication
- ✓ electrocardiography
- ✓ Needle site observation
- ✓ Laboratory tests (routine blood, blood biochemistry, CRP, C-reactive protein; Lp-PLA2, lipoprotein-associated phospholipase A2; C-FABP, cardiac fatty acid-binding protein level;)
- ✓ Adverse event assessment

6.5 Safety follow-up: visits 6 and 7 (week 16 ± 2 days, week 24 ± 2 days)

- ✓ Concomitant Diseases, Concomitant Treatments/Medications
- ✓ clinical examination
- ✓ vital signs
- ✓ PSQI scale scores
- ✓ Seattle Angina Scale Score
- ✓ Hamilton Depression Scale (HAMD)
- ✓ Generalized Anxiety Disorder Scale (GAD-7)
- ✓ Health-related Quality of Life Questionnaire (SF-36)
- ✓ Use of sleeping medication
- ✓ electrocardiography
- ✓ Needle site observation
- ✓ Adverse event assessment

6.6 Unplanned visits

For reasons of subject safety, the investigator may request additional visits or examinations of the subject. The results of any unplanned visits or examinations are to be documented in the original medical record and case report form.

6.7 Early withdrawal

When a subject decides to withdraw from the clinical trial for various reasons, in order to protect his/her safety, the investigator needs to communicate with the subject and try to persuade him/her to complete the relevant examinations and assessments required for the current visit. If the subject insists on refusing the current visit, then the information of the last visit before the subject's withdrawal will be used as the data for the early discontinuation of the visit, which will be used for the evaluation of the effectiveness and safety. For subjects who withdraw early, the investigator should record the reason for withdrawal, the date of withdrawal, and other relevant information in the study medical record.

6.8 Precautions during the test

① The investigator instructed the subjects to pay attention to the following during the study;

② Avoid stimulating foods and alcohol during the test;

③ Maintain a stable diet, routine and lifestyle;

④ Avoid emotional fluctuations, usually keep a relaxed mood, avoid mental stimulation, relieve all kinds of mental stress.

⑤ Moderate exercise is allowed during the test period, try to avoid strenuous exercise and prolonged exertion;

7. Efficacy and safety evaluation criteria

7.1Primary outcomes

The Pittsburgh Sleep Quality Index (PSQI), comprising 19 self-rated and 5 hetero-rated items, will be employed to evaluate the primary outcome of global sleep quality over the past month across 7 components: sleep duration, sleep latency, sleep efficiency, sleep medication use, sleep disturbances, subjective sleep quality, and daytime dysfunction. PSQI will be assessed at baseline, after each 4-week treatment phase (weeks 4, 8, 12), and during follow-up at weeks 16 and 24.

7.2 Secondary Outcomes

① Assessment of stable angina pectoris (SAP): SAP was evaluated at baseline, week 4, week 8, and week 12 using the Seattle Angina Questionnaire (SAQ) to quantify angina symptoms before and after

treatment. Blood biomarkers of SAP: Fasting venous blood samples were collected from the cubital fossa on the start and final of the treatment period in both groups. Levels of C-reactive protein (CRP), lipoprotein-associated phospholipase A2 (Lp-PLA2), and cardiac fatty acid-binding protein (C-FABP) were assayed by an independent laboratory blinded to group allocation.

② Quality of life assessment using the 36-Item Short Form Health Survey (SF-36).

③ Dosage of sleeping pills.

④ Hamilton Depression Rating Scale (HAMD).

⑤ Generalized Anxiety Disorder 7-item (GAD-7) scale.

7.3 Security indicators

① Vital signs (temperature, blood pressure, respiratory rate, heart rate).

② Routine blood and urine tests.

③ Liver and kidney function tests.

④ Electrocardiogram.

⑤ Adverse events.

7.4 Security evaluation

7.4.1 Definitions and provisions related to adverse events

① Definitions and regulations related to adverse events: Adverse Event (AE) refers to all adverse medical events that occur after a subject receives an experimental medication, which may be manifested as signs and symptoms, disease, or abnormal laboratory tests, but are not necessarily causally related to the experimental medication. An Adverse Event can be any unhelpful or undesired sign (including abnormal laboratory test values), symptom, or temporary illness that occurs concomitantly with the use of the medication, whether or not the illness is related to the medication.

② Serious Adverse Event (Serious Adverse Event, SAE) refers to an adverse medical event such as death, life-threatening, permanent or serious disability or loss of function, hospitalization or prolonged hospitalization of the subject, as well as congenital anomalies or birth defects, etc., which occur after the

subject receives the experimental drug.

③Suspicious and Unexpected Serious Adverse Reactions (SUSAR) are serious adverse reactions that are suspected and unanticipated because the nature and severity of the clinical manifestations are beyond the information already available in the investigator's manual of the test drug, the instruction manual of the marketed drug, or the summary of product characteristics. Adverse Reactions.

7.4.2 Adverse event records

Adverse event record form was faithfully completed during the trial to record the occurrence event, severity, duration, effective measures taken and regression of the adverse event.

7.4.3 Criteria for determining severity

All adverse events that occurred during the course of the trial were determined in terms of severity based on the following criteria.

Grade I: Mild; asymptomatic or mildly symptomatic; clinical or diagnostic findings only; no treatment required;

Grade II: Moderate; minimal, localized or non-invasive indication for treatment; age-related instrumental limitation of activities of daily living;

Grade III: Severe or medically significant but not immediately life-threatening; indication for hospitalization or prolonged hospitalization; disabling; self-initiated limitation of activities of daily living;

Grade IV: life-threatening, requiring urgent treatment;

Grade V: death.

7.4.4 Adverse event and drug causality determination

The analysis of adverse events in relation to treatment requires a combination of the following factors:

① The sequence of the time of treatment initiation and the time of occurrence of adverse events, and whether there is a clear causal relationship.

②Whether the presentation of the adverse event is consistent with the known adverse reaction profile of

that treatment.

③Whether the adverse event may have been caused by a combination of medications, the patient's clinical condition, or other treatment modalities.

④Whether the adverse events diminished or disappeared after stopping treatment.

⑤Whether or not the same adverse effects occur again when treatment is resumed.

7.4.5 Adverse Event Detection, Tracking, and Follow-Up

Adverse events will be obtained by verbal questioning and documented in the study medical record. To ensure consistency of assessment, the investigator or designee should use the same question wording for each interrogation to avoid causing inter-subject variability.

The investigator will also need to ask the subject about any previously unresolved adverse events. The investigator will need to assess the severity and intensity of the adverse event, its relevance to the study medication, and the measures taken to address it. The investigator must ask the subject these questions at each return visit or corresponding termination visit.

Laboratories participating in the clinical trial should conduct testing in accordance with standard operating procedures and quality control procedures. Participating laboratories should provide their "Laboratory Test Normal Value Ranges", which should be updated with any changes.

All adverse events should be followed up until appropriately resolved or stabilized. Adverse events based on laboratory test values should be followed up until the laboratory results return to normal or remain stable, and the process of management, follow-up data, and regression should be fully documented in the study chart, with the relevant examination report card attached.

7.4.6 Reporting procedures and handling

①Adverse event reporting procedures and handling

The occurrence of any adverse events, such as subjective discomfort of the subject and abnormal laboratory tests, should be taken seriously, carefully analyzed, and immediate measures should be taken to protect the safety of the subject. And record the details of the adverse event in the study medical record, and record its persistence, regression disappearance, etc. as appropriate.

② Reporting procedures and handling of serious adverse events

In the event of a serious adverse event (SAE), the investigator must immediately take appropriate and active rescue and protection measures for the subject, make every effort to ensure the safety of the subject, and decide whether to terminate the trial according to the condition. Investigators should collect SAE-related information, such as medical records and examination results, in order to fill out SAE reports accurately and timely, and report them to the sponsor, the clinical trial organization office, and the Ethics Committee in the form of Serious Adverse Event Report Form within 24 hours.

All SAEs should be followed up by the investigator. The follow-up should be recorded by the investigator in the Serious Adverse Event Report Form/Serious Adverse Event Follow-up Report and reported to the sponsor, clinical trial organization office and ethics committee within 24 hours. The frequency of follow-up was decided by the investigator according to the specific situation of SAE to ensure timely access to the latest information until the final result occurred. Once the symptoms of SAE disappeared or stabilized, or the subject was lost or died, the investigator would submit a written summary report to the sponsor and report to the ethics committee and the institution.

If the SAE is a fatal event, the investigator should provide other required information, such as autopsy report and final medical report, to the sponsor, the ethics committee and the drug clinical trial organization. The written report should indicate the subject's identification code in the clinical trial; the information reported should be complete and accurate to provide a basis for the sponsor's assessment.

The information in the study chart and electronic case report form (eCRF) should be consistent with the records in the SAE report form and the source material, and the SAE report form should be signed and dated by the investigator and kept in a secure place at the clinical trial site.

If a serious adverse event or reaction occurs during a clinical trial, it is necessary to know the drug group of the subject for appropriate management and timely treatment. In such an emergency situation, the principal investigator has the right to decide on emergency blinding, and record in detail the reason, time and place of blinding, and sign and keep a file. The sponsor and the clinical investigator should be notified promptly of the blinding. Once blinded, the subject will be terminated from the trial and treated as a dropout case, and the results should be notified to the supervisor with detailed reasons, date and signature in the original study chart.

7.4.7 Follow-up of unresolved adverse events

All adverse events should be recorded and followed up in a timely manner until they are properly resolved or the condition is stabilized. Abnormal indicators that appear on physical and chemical tests after treatment should be promptly reviewed until normal or stable.

8. Risk management and risk assessment

①When screening subjects, the inclusion and exclusion criteria must be strictly followed to ensure that the requirements are met.

② The informed consent form should clearly inform subjects of the possible risks of participating in the study and make them aware of the relevant information.

③Before the study, check whether the emergency medicines are complete, whether the expiration date is still within the validity period, and check regularly during the study to ensure the availability of emergency medicines.

④A comprehensive emergency plan should be developed prior to the start of the study, and contact procedures should be established with the intensive care unit of the hospital for the transfer and care of subjects, so that a timely response can be made when necessary.

⑤ A communication and exchange mechanism between the investigator and the laboratory should be established to ensure that clinical laboratory critical values can be communicated and handled in a timely manner to avoid delays.

9. Quality control and quality assurance

①Quality control measures

Investigators should adopt standard operating procedures to ensure the implementation of quality control and quality assurance system of clinical trials. All observations and findings in the clinical trial should be verified to ensure the reliability of the data and to ensure that the conclusions in the clinical trial are derived from the original data. Quality control must be applied at every stage of data processing to ensure that all data are reliable and correctly processed.

②Investigator training

Before the start of the clinical trial, the supervisor, together with the person in charge of each trial center, should train the investigators on the trial protocol, in order to make the investigators understand and be

familiar with the nature of the treatment, its effects, efficacy and safety, and also to grasp all the new information related to the drug found during the clinical trial.

③ Improvement of subject compliance

Investigators should carefully implement informed consent so that subjects fully understand the trial requirements and cooperate with the trial. Investigators should also require patients to bring all the medications they are using to the follow-up visit to check the patient's comorbid medications and record them on the case report form, and follow-up should be enhanced for patients with poorer efficacy and those who are unable to take their medications on time.

④ Supervision of clinical trials

Supervisors are appointed by the principal investigator to conduct regular supervisory visits to the trial hospital site to ensure that all elements of the study protocol are strictly adhered to, and that the original data are checked to ensure consistency with those on the CRF.

10. Data management and verification

10.1 Data management plan

The data management manager develops a data management plan based on GCP-related principles and clinical trial-related content (e.g., protocols, CRFs, project realities, etc.). The data management plan will document, describe and define the various tasks of data management, thus guiding the entire data management process. The data management plan should include: database establishment, data entry, data verification, questionnaire management, medical coding, database locking, data preservation, etc. It also defines some time points for data management and clarifies the responsibilities of relevant personnel. During the intervention period, study data were collected and recorded according to the protocol through electronic transcription of paper case report forms, and data were entered within 1 week of each study visit using EpiData software, which enabled dual data entry with logical validation and with verification to ensure accuracy. An independent data manager external to the study oversaw data entry, validation, security, and resolution of outliers outside the range of clinical expectations. Finally, after sign-off by the study sponsor, principal investigator, biostatistician, and data manager, the database was locked for analysis and no further modifications were allowed.

10.2 Data verification, cleaning and quality control

① Data verification

The data verification plan was developed by the data management statistics department, medical technology department and the project manager on the premise of fully grasping the design of the program based on the program's data management content of all the verification points to be determined in a specific discussion. If changes are needed during the implementation process, the data manager will revise and update the version, and again signed by the above departments to confirm.

② Data Cleaning

Questions during the data verification process are generated in the form of questions automatically generated by the online system and manually issued questions, which are answered online by the researcher until the data is cleaned up. All final queries are confirmed and signed by the researcher. An electronic version of the query form was kept by the data management department.

③ Data quality control

The use of real-time edit verification, any non-compliance with QC or QA requirements, the need to take action requirements and tracking procedures are documented and corrected. During the implementation of the QC process, a list of data quality that does not meet the specifications should be made. This part is a dynamic record that needs to be summarized and communicated in a meeting by the data manager, supervisor or auditor based on the actual list of percentage of errors found.

10.3 Data lock and exit

Completion of the data lock list, according to the procedures of database locking, signed by the data manager, statistical analysts, representatives of clinical supervisors, researchers and other written approval of the database lock document, by the data manager will be exported to the database in the specified format, and handed over to the statistician for statistical analysis. After data locking, if there is solid evidence that unlocking is necessary, the investigator and related personnel need to sign the unlocking document.

11. Statistical analysis

Group allocation will be concealed from the statistician. All randomized participants will be included in the intention-to-treat (ITT) analysis, regardless of study completion status. To ensure accuracy, double data entry will be performed. Statistical analyses will be conducted using SPSS 24.0. Continuous

variables will be summarized as mean ± standard deviation or median (interquartile range). Between-group comparisons will utilize two-sample t-tests or non-parametric tests for skewed data. Within-group baseline to endpoint changes will be analyzed by paired t-tests. Categorical variables will be compared using Fisher's exact or Chi-square tests. Statistical significance will be defined as $p<0.05$.

12.Ethics and subject protection

12.1 Ethical Guidelines

This study will be conducted in accordance with the ethical principles of the Declaration of Helsinki and in compliance with the Quality Management Practice for Clinical Trials (GCP), applicable regulatory requirements policy.

12.2 Ethics Committee

The clinical trial protocol needs to be developed jointly by the principal investigator and the sponsor after full consultation. Prior to the commencement of the clinical trial, the investigator must obtain written approval from the Ethics Committee, and may not begin screening subjects without such approval. If there is a need to revise the protocol during the course of implementation, the protocol must be submitted to the Ethics Committee again for approval, and the revision can be implemented only after approval is obtained.

In the course of the trial, if the investigator obtains new information that may affect the continued participation of the subjects, the investigator should inform the subjects or their legal representatives in writing in the form of an informed consent form. After obtaining approval from the Ethics Committee for all new information, subjects should sign the informed consent form again to indicate their knowledge and consent. Newly enrolled subjects were required to sign the updated informed consent form and other relevant documents.

12.3 Informed Consent Process

The principal investigator of the research center (or his/her designated representative) will:

① During the informed consent process, the investigator must follow the Code of Ethics for the Quality Management of Clinical Trials and the Declaration of Helsinki, and must not use any improper means such as coercion or inducement to influence the subject's autonomous decision.

② All written or verbal information related to the trial shall not contain any language that deprives subjects and their legal representatives of their legitimate rights and interests, nor shall it exempt the investigator, the medical institution, the sponsor, or the agent from their responsibilities.

③The investigator or designated personnel shall fully inform the subjects of all relevant matters concerning the trial, including written information and the approval opinion of the Ethics Committee. For subjects who are incapable of expressing informed consent, their legal representatives shall make the decision on their behalf.

④Oral and written information such as the informed consent form provided to the subjects shall be in easy-to-understand language and expression so as to be easily understood by the subjects or their legal representatives or witnesses.

⑤ Before signing the informed consent, the subject or legal representative shall be given sufficient time and opportunity to understand the details of the test, and all relevant questions shall be answered.

⑥The subject or legal representative and the investigator giving informed consent should sign and date the informed consent form separately.

⑦If the subject or legal representative lacks the ability to read, an impartial witness is required to assist and witness the informed consent process. The investigator should explain in detail the contents of the informed consent form and other written materials. After the subject or legal representative has given verbal consent and signed, the witness must also sign and date the informed consent form to prove that the subject or legal representative has been accurately explained and understands the relevant content, and participates voluntarily.

⑧ The subject or legal representative should be provided with a copy of the signed informed consent form and other written information; an updated version of the informed consent form and a revised version should also be provided during the trial.

⑨ When the legal representative for the informed consent, should try to let the subject understand the relevant information, and as far as possible by the subject to sign the informed consent form.

⑩In emergency situations, if prior informed consent cannot be obtained from the subject, the consent of the legal representative must be obtained. If neither the subject nor his/her legal representative is present, the method of enrollment should be clearly stated in the trial protocol and documents and approved by

the Ethics Committee, and informed consent for continued participation should be obtained as soon as possible.

12.4 Medical Treatment and Protection of Subjects

Physicians participating in a clinical trial are responsible for all trial-related medical decisions. During the trial and follow-up period, if a trial-related adverse event or clinically significant laboratory abnormality occurs in a subject, the investigator and the host institution must ensure that the subject receives appropriate medical treatment and is informed of the situation; the investigator should also be alert to the presence of other underlying diseases.

Subject to consent, the investigator may inform the subject's treating physician of the subject's participation in the trial in order to obtain more comprehensive medical information.

Subjects have the right to withdraw from the clinical trial at any time without reason. While respecting the subject's right, the investigator should endeavor to understand the reason for withdrawal in order to improve the subsequent trial design and operational procedures.

Protecting the rights and interests of subjects is the most important aspect of a clinical trial, and the investigator and the trial organization have the responsibility to provide adequate medical protection for subjects and to respect their right to make their own decisions, so as to ensure that the trial is conducted in a safe and ethical environment.

12.5 Protection of Subject Privacy

Access to subjects' personal medical records will be strictly limited to the investigators and monitors directly involved in the clinical trial, who are required to sign an "Investigator's Declaration" or "Confidentiality Commitment", promising to abide by the terms of confidentiality. The drug regulatory authorities will have access to clinical trial records in the course of law enforcement and supervision.

During data processing, anonymization will be used to remove all information that could lead to the identification of individual subjects, in order to protect the privacy of the subjects. All clinical trial data will be kept by the hospital's designated management department.

In addition, all relevant personnel involved in the clinical trial are obliged to abide by professional ethics, and to maintain strict confidentiality of the subjects' personal information contacted during the trial to ensure that the subjects' privacy and data security are adequately protected, and to safeguard the

legitimate rights and interests of the subjects.

12.6 Recruitment of subjects

Recruitment methods include direct recruitment from the clinical medical process, open recruitment, through the database for screening recruitment. Recruitment advertisements should be located in clinical environments, public places and online platforms to ensure sufficient sample increase.

13. Quality Assurance and Quality Control

Good quality control is essential to ensure the validity and safety of clinical trial data. Prior to the conduct of a clinical trial, it is important to anticipate and take steps to address potential issues that may affect the quality of the trial. It is the responsibility of the sponsor to develop, implement and update relevant standard operating procedures (SOPs) and establish a comprehensive quality assurance and quality control system to ensure that all aspects of the clinical trial process, data generation, recording and reporting are in strict compliance with the trial protocols, Good Clinical Practice (GCP) and relevant laws and regulations, in order to ensure the quality of the trial.

13.1 Quality control of subjective symptom evaluation

In order to ensure the consistency of symptom evaluation among different investigators, a uniform evaluation standard should be formulated before the implementation of the trial, and uniform training should be provided to all investigators, which should be verified by consistency test. The training should be provided to the principal investigators, researchers, medication administrators, laboratory personnel, nursing staff and other participants in the centers, so that they can fully understand the protocol and the requirements of the relevant guidelines.

13.2 Quality Control of Laboratory Tests

Laboratories of medical institutions participating in clinical trials should establish quality management standards and operation norms to ensure the accuracy and reliability of test data and results.

13.3 Clinical monitoring/audit quality control

The sponsor shall appoint a qualified auditor who has received appropriate training and possesses the necessary scientific and clinical knowledge for clinical trial monitoring. The sponsor should develop a monitoring plan and standard operating procedures (SOPs), which should be followed by the monitors

during the monitoring work.

14. Data retention

The investigator and his/her medical institution should keep the trial documents in accordance with the "Essential Documents for Clinical Trials" and the relevant requirements of the drug regulatory authorities. The essential documents are categorized into three phases according to the different phases of the clinical trial: the preparation phase of the clinical trial, the conduct phase of the clinical trial, and the phase of the clinical trial after completion of the clinical trial. The necessary documents include the true and accurate confirmation of all subjects' conditions (able to effectively check different record information, such as the original records of the hospital), all original informed consent forms signed by the subjects, and detailed records of all medical record registration forms. All cases, whether observation is completed or dislodged, should complete the study medical record as required. The study medical records shall be archived by each participating unit as original data; the informed consent and study medical records shall be kept until or 5 years after the termination of the clinical trial (whichever is the longest).

References

1. Y L, L Z, S L, X W, J Z, X T, et al. Efficacy and safety of oral Guanxinshutong capsules in patients with stable angina pectoris in China: a prospective, multicenter, double-blind, placebo-controlled, randomized clinical trial. BMC complementary and alternative medicine. 2019;19. doi:10.1186/s12906-019- 2778-z
2. P H, Z L, L C, J Z, S Z, Y T, et al. The comparative effects of oral Chinese patent medicines combined with western medicine in stable angina: A systematic review and network meta-analysis of 179 trials. Frontiers in pharmacology. 2022;13. doi:10.3389/fphar.2022.918689
3. J W, W G, G C, M C, Z W, W Z, et al. Biomarker-based risk model to predict cardiovascular events in patients with acute coronary syndromes - Results from BIPass registry. The Lancet regional health Western Pacific. 2022;25. doi:10.1016/j.lanwpc.2022.100479
4. H H, Az J, Mh H, E V, Sm M, A M. The effects of Melissa officinalis supplementation on depression, anxiety, stress, and sleep disorder in patients with chronic stable angina. Clinical nutrition ESPEN. 2018;26. doi:10.1016/j.clnesp.2018.04.015
5. Y W, Y Z, Y D, Y Y, J W, N Z, et al. A multicenter, randomized, double-blind, placebo-controlled trial evaluating the efficacy and safety of Taoren Honghua Jian granule in patients with stable coronary artery disease. Medicine. 2019;98. doi:10.1097/MD.0000000000017753
6. M S-L, Eb L, B L, Ma C, K S, Ml S, et al. Sleep duration, insomnia, and coronary heart disease among postmenopausal women in the Women's Health Initiative. Journal of women's health

(2002). 2013;22. doi:10.1089/jwh.2012.3918
7. M S, M Z, L H, D F, P S. Assessment of the climacteric syndrome: a narrative review. Archives of gynecology and obstetrics. 2021;304. doi:10.1007/s00404-021-06139-y
8. A M, L A, Av P. Is post-ischemic stroke insomnia related to a negative functional and cognitive outcome? Sleep medicine. 2022;94. doi:10.1016/j.sleep.2022.03.022
9. P M, A S, D S. Restricted and disrupted sleep: effects on autonomic function, neuroendocrine stress systems and stress responsivity. Sleep medicine reviews. 2008;12. doi:10.1016/j.smrv.2007.07.007
10. B S, T L, P S, S P, M H, S K, et al. Insomnia as a risk factor for ill health: results from the large population-based prospective HUNT Study in Norway. Journal of sleep research. 2014;23. doi:10.1111/jsr.12102
11. Rq L, Z Q, E T, Jj C, A Z, Yt H, et al. Poor sleep quality associated with high risk of hypertension and elevated blood pressure in China: results from a large population-based study. Hypertension research : official journal of the Japanese Society of Hypertension. 2016;39. doi:10.1038/hr.2015.98
12. Vr T, S S, A M, A T. Menopause and Sleep Disorders. Journal of mid-life health. 2022;13. doi:10.4103/jmh.jmh_18_22
13. Cm M, Dc J. Epidemiology of Insomnia: Prevalence, Course, Risk Factors, and Public Health Burden. Sleep medicine clinics. 2022;17. doi:10.1016/j.jsmc.2022.03.003
14. Lr R, M L-P, Dr P, Ne E, Dm L, Ml K. Detoxification from high-dose zolpidem using diazepam. The Annals of pharmacotherapy. 2004;38. doi:10.1345/aph.1D339
15. C L, Y Z, S Q, X W, Y J, W W. Randomized controlled trial of acupuncture for anxiety and depression in patients with chronic insomnia. Annals of translational medicine. 2021;9. doi:10.21037/atm-21-3845
16. Zhou L, Hu X, Yu Z, Yang L, Wan R, Liu H, et al. Efficacy and Safety of Acupuncture in the Treatment of Poststroke Insomnia: A Systematic Review and Meta-Analysis of Twenty-Six Randomized Controlled Trials. Seong Lin T, editor. Evidence-Based Complementary and Alternative Medicine. 2022;2022: 1–16. doi:10.1155/2022/5188311
17. Y L, H Z, Q W, C T, H L, L H, et al. Comparative effectiveness of multiple acupuncture therapies for primary insomnia: a systematic review and network meta-analysis of randomized trial. Sleep medicine. 2022;93. doi:10.1016/j.sleep.2022.03.012
18. Effects of electroacupuncture at “Anmian (Extra)” acupoints on sleep activities in rats: the implication of the caud al nucleus tractus solitarius - PubMed. [cited 13 Nov 2023]. Available: https://pubmed.99885.net/15316132/
19. Sy S, Jy M, Sy K, Os K, Sk B, Kh C, et al. Acupuncture stimulation at HT7 as a non-

pharmacological therapy for sleep disorder caused by caffeine administration in rats. Acupuncture in medicine : journal of the British Medical Acupuncture Society. 2021;39. doi:10.1177/09645284211011489

20. Efficacy and safety of acupuncture treatment on primary insomnia: a randomized controlled trial - PubMed. [cited 13 Nov 2023]. Available: https://pubmed.99885.net/28899535/

21. Mao LL, Jin YP. [Professor JIN Ya-pei's experience in the treatment of perimenopausal insomnia with navel acupuncture combined with body acupuncture]. Zhongguo Zhen Jiu. 2020;40(3):295-8

22. C F, N Z, Z L, Lh Y, C X, Wj Y, et al. Acupuncture Improves Peri-menopausal Insomnia: A Randomized Controlled Trial. Sleep. 2017;40. doi:10.1093/sleep/zsx153

23. Li O, Wang F. [Acupuncture at back-shu points of five zang, Geshu (BL 17) and Shenmen (HT 7) for the treatment of menopausal insomnia]. Zhongguo Zhen Jiu. 2018;38(5):4693-72

24. T N, M H, Sn G. Assessment of the Frequency and Variety of Persistent Symptoms Among Patients With COVID-19: A Systematic Review. JAMA network open. 2021;4. doi:10.1001/jamanetworkopen.2021.11417

25. A T, Sm GH, H T, A A, M I, A S, et al. COVID-19 and cardiac injury: clinical manifestations, biomarkers, mechanisms, diagnosis, treatment, and follow up. Expert review of anti-infective therapy. 2021;19. doi:10.1080/14787210.2020.1822737

Appendix 1 Pittsburgh Sleep Quality Index

Guideline: Some of the questions below are about your sleep in the last 1 month, please choose back to fill in the answer that best matches your actual situation in the last 1 month. Please answer the following questions!

1. In the last month, you usually went to bed at night at -- o'clock.

2. In the past month, it usually took me -- minutes to fall asleep after going to bed. 3.

3. in the last month, I usually get up at - o'clock in the morning. 4. in the last month, I usually get up at - o'clock in the morning.

4. in the last month, you usually slept - hours per night (not equal to bedtime). Please choose the one answer to the following questions that best suits you.

5. In the past month, you have had trouble sleeping because of the following: a. Difficulty falling asleep (cannot fall asleep within 30 minutes) (1) None (2) <1 time/week (3) 1-2 times/week (4) ≥ 3 times/week b. Waking up easily or early at night (1) None (2) <1 time/week (3) 1-2 times/week (4) ≥ 3 times/week c. Going to the toilet at night (1) None (2) <1 time/week (3) 1-2 times/week (4) ≥ 3 times/week d. Going to the toilet at night (1) None (2) <1 time/week (3) 1-3 times/week (4) ≥ 3 times/week e. Going to the bathroom at night (1) None (2) <1 time/week (3) <1-3 times/week (4) <1-3 times/week (4) ≥ 3 times/week d. Breathing problems (1) none (2) <1 time/week (3) 1-2 times/week (4) ≥ 3 times/week e. Coughing or snoring (1) none (2) <1 time/week (3) 1-2 times/week (4) ≥ 3 times/week f. Feeling cold (1) none (2) <1 time/week (3) 1-2 times/week (4) ≥3 times/week g. Feeling hot (1) none (2) <1 time/week (3) ≥1 time/week (3) ≥1 time/week (4) ≥3 times/week g. Feeling hot (1) none (2) <1 time/week (5) <1 time/week (5) <1 time/week (6) <1 time/week (6) <1 time/week (6) <1 time/week (7) <1 time/week (9) <1 time/week (8) <1 time/week (10) <1 time/week (11) <1 time/week (12) <1 time/week (13) <3 times/week times/week (3) 1-2 times/week (4) ≥ 3 times/week h. Nightmares (1) none (2) < 1 time/week (3) 1-2 times/week (4) ≥ 3 times/week i. Pain and discomfort (1) none (2) < 1 time/week (3) 1-2 times/week (4) ≥ 3 times/week j. Other things that interfere with sleep (1) none (2) < 1 time/week (3) 1-2 times/week (4) ≥ 3 times/week If yes, please describe:

6. In the past month, in general, do you think your sleep quality is (1) very good (2) good (3) poor (4) very poor?

7. In the past month, have you used medication for hypnosis (1) None (2) <1 time/week (3) 1-2 times/week (4) ≥ 3 times/week?

8 In the past month, do you often feel sleepy (1) No (2) <1 time/week (3) 1-2 times/week (4) ≥ 3 times/week

9. In the past month, do you have low energy to do things (1) No (2) Occasionally (3) Sometimes (4) Often

Usage and Statistical Method

PSQI was used to rate the subjects' sleep quality in the last 1 month. The PSQI consists of 19 self-assessment and 5 other-assessment items, of which the 19th self-assessment and 5 other-assessment items are not involved in the scoring, and only the 18 self-assessment items that are involved in the scoring are introduced here (see the attached questionnaire for details). The 18 items are composed of 7 components, and each component is scored according to a scale of o-3, and the cumulative scores of the components are the total PSQI score, with a range of 0-2l, and the higher the score, the worse the sleep quality is. It took 5 to 10 minutes to complete the questionnaire. The meaning of each component

and the scoring method are as follows:

A. Sleep quality: According to the response to entry 6, "good" scores 1, "poor" scores 2, and "very poor" scores 3.

B. Time to sleep 1. The score for entry 2 is "≤ 15 points" (0 points), "16-30 points" (1 point), "31-60 points" (2 points), "≥ 60 points" (1 point), "≥ 60 points" (1 point), "≥ 60 points" (1 point), "≥ 60 points" (1 point), "≥ 60 points" (1 point). "≥ 60 points" is 3 points. 2. 2. Entry 5a is scored as "none" (0 points), "<1 week/times" (1 point), "1~2 weeks/times" (2 points), "≥3 weeks/times" (3 points). "3. Cumulative entries 2 and 3 3. For cumulative entries 2 and 5a, if the cumulative score is "0", 0 points, "1~2", 1 point, "3~4", 2 points, "5~6", 3 points. 3 points for "5~6".

C Sleeping time Score based on response to entry 4: ">7 hours" 0 points, "6-7" 1 point, "5-6" 2 points, "<5 hours" 3 points. 5 hours" is scored as 3 points.

D Sleep efficiency 1. time in bed = entry 3 (time in bed) - entry 1 (time in bed) 2. sleep efficiency = entry 4 (time in bed) / time in bed × 100% 3. Component D scoring position, sleep efficiency > 85% scores 0 points, 75-84% scores 1 point, 65-74% scores 2 points, and < 65% scores 3 points.

E According to the scores of items 5b to 5j, "none" is scored as 0, "<1 week/times" as 1, "1-2 weeks/times" as 2, "≥3 weeks/times" as 2, and "≥3 weeks/times" as 2. "≥3 weeks/times" is 3 points. For cumulative entries 5b to 5j, if the cumulative score is "0", component E will be scored as 0, "1~9" will be scored as 1, "10~18" will be scored as 2, "19~27" will be scored as 2, and "19~27" will be scored as 1, "1~2 weeks/times" will be scored as 2. "19~27" will be scored as 3 points.

F Hypnotic drugs According to the response of entry 7, "none" is scored as 0 points, "<1 week/times" is scored as 1 point, "1-2 weeks/times" is scored as 2 points, "≥3 weeks/times" is scored as 2 points, "≥3 weeks/times" is scored as 2 points, "≥3 weeks/times" is scored as 3 points. "≥3 weeks/times" is 3 points.

G Daytime dysfunction: 1. Score based on response to entry 8, with "none" scoring 0, "<1 week/times" scoring 1, "1-2 weeks/times" scoring 2, "≥3 weeks/times" scoring 2, "≥3 weeks/times" scoring 3, and "≥3 weeks/times" scoring 3. "≥3 weeks/times" is scored as 3 points. 2. 2. Points are awarded for responses to entry 9, with 0 points for "no", 1 point for "occasionally", 2 points for "sometimes", and 3 points for "often". "3. Cumulative entries 8 and 8 3. Score for cumulative entries 8 and 9. If the cumulative score is "0", component G will be scored 0, "1~2" will be scored 1, "3~4" will be scored 2, "5~6" will be scored 2, and "5~6" will be scored 3. "5~6" will be scored 3 points.

Total PSQI score = Ingredient A + Ingredient B + Ingredient C + Ingredient D + Ingredient E + Ingredient F + Ingredient G

Appendix 2

SF-36 Quality of Life Form Questionnaire

No.: Name: Gender: Age Management category:

1、Generally speaking, your health condition is:

①very good ②very good ③good ④fair ⑤poor

(Weights or scores are in the order of 5, 4, 3, 2, 1)

2、Compared with 1 year ago, you think your health condition is:

① much better than 1 year ago ② better than 1 year ago ③ about the same as 1 year ago ④ worse than 1 year ago ⑤ much worse than 1 year ago (weights or scores in the order of 5, 4, 3, 2, 1)

Health and daily activities

3. All of these questions below are related to daily activities. Please think about whether your health limits these activities. If it does limit them, to what extent?

(1) Heavy physical activities. For example, running and lifting weights, participating in strenuous exercise, etc:

(① very restrictive ② somewhat restrictive ③ no restriction at all)

(Weights or scores in order of 1, 2, 3; same below)

(2) Moderate activities. Such as moving a table, sweeping the floor, playing tai chi, and doing simple gymnastics:

(1) very restrictive ② somewhat restrictive ③ no restriction at all

(3) Hand-carrying daily necessities. Such as grocery shopping, shopping, etc:

① very restrictive ② somewhat restrictive ③ no restriction at all

(4) Going up several flights of stairs: ①Large restrictions ②Some restrictions ③No restrictions at all

(5) Going up one flight of stairs: ①Large restrictions ②Some restrictions ③No restrictions at all

(6) Bending, stooping, squatting: ①Large restrictions ②Some restrictions ③No restrictions at all

(7) Walking a distance of 1500 meters or more: ①Large limitations ②Some limitations ③No limitations

(8) Walking a distance of 1000 meters: ① a great deal of restrictions ② some restrictions ③ no restrictions

(9) Walking a distance of 100 meters: ①Large limitations ②Some limitations ③No limitations at all

(10) Bathing and dressing by yourself: ①Large limitations ②Some limitations ③No limitations at all

4、During the past 4 weeks, have you had any of the following problems with your work and daily activities due to your physical health?

(1) Reduced time for work or other activities: ① Yes ② No

(Weights or scores in order of 1, 2; same below)

(2) Only part of what you wanted to do was accomplished: ①Yes ②No

(3) The type of work or activity you want to do is limited: ①Yes ②No

(4) It has become more difficult to complete work or other activities (e.g., extra effort is required): ① Yes ②No

5、During the past 4 weeks, have you had any of the following problems with your work or daily activities due to emotional reasons (e.g., depression or worry)?

(1) Reduced time for work or activities: ①Yes ②No

(Weights or scores in order of 1, 2; same below)

(2) Only part of what you wanted to do was accomplished: ①Yes ②No

(3) Not as careful as usual: ①Yes ②No

6、During the past 4 weeks, to what extent has your poor health or mood affected your normal social interactions with family, friends, neighbors, or groups?

①No effect at all ②A little effect ③Moderate effect ④Large effect ⑤Very large effect

(Weights or scores in order of 5, 4, 3, 2, 1)

7. Have you had any body pain in the past 4 weeks?

① No pain at all ② A little pain ③ Moderate pain ④ Severe pain ⑤ Very severe pain

(Weight or score in order of 6, 5.4, 4.2, 3.1, 2.2, 1)

8、Did your body pain affect your work and housework in the past 4 weeks?

① No effect at all ② A little effect ③ Moderate effect ④ Great effect ⑤ Very great effect

(If 7 no 8 no, the weight or score is 6, 4.75, 3.5, 2.25, 1.0 in order; if 7 yes 8 no, 5, 4, 3, 2, 1)

How you feel

9. These following questions are about how you have felt about yourself in the past 1 month, and for each of the things the questions say, what has been your situation?

(1) You feel that life is full:

(1) All of the time (2) Most of the time (3) More of the time (4) Some of the time (5) A small part of the time (6) Don't have that feeling

(Weights or scores in order of 6, 5, 4, 3, 2, 1)

(2) You are a sensitive person:

① all the time ② most of the time ③ more of the time ④ part of the time ⑤ a small part of the time ⑥ no such feeling

(Weights or scores in order of 1, 2, 3, 4, 5, 6)

(3) You are in a very bad mood and nothing can cheer you up:

① all the time ② most of the time ③ more of the time ④ part of the time ⑤ a small part of the time ⑥ no such feeling (weight or score in order of 1, 2, 3, 4, 5, 6)

(4) You are mentally calm:

① all of the time ② most of the time ③ more of the time ④ part of the time ⑤ a small part of the time ⑥ no such feeling (Weight or score in order of 6, 5, 4, 3, 2, 1)

(5) You do things energetically:

① all of the time ② most of the time ③ more of the time ④ part of the time ⑤ a small part of the time ⑥ no such feeling (weight or score in the order of 6, 5, 4, 3, 2, 1)

(6) Your mood is low:

① all of the time ② most of the time ③ more of the time ④ part of the time ⑤ a small part of the time ⑥ no such feeling (Weight or score in order of 1, 2, 3, 4, 5, 6)

(7) You feel exhausted:

① all of the time ② most of the time ③ more of the time ④ some of the time ⑤ a small part of the time ⑥ no such feeling (Weight or score in order of 1, 2, 3, 4, 5, 6)

(8) You are a happy person:

① all of the time ② most of the time ③ more of the time ④ part of the time ⑤ a small part of the time ⑥ no such feeling (weight or score in order of 6, 5, 4, 3, 2, 1)

(9) You feel bored:

① all of the time ② most of the time ③ more of the time ④ part of the time ⑤ a small part of the time ⑥ no such feeling (Weight or score in order of 1, 2, 3, 4, 5, 6)

10、Unhealthy affects your social activities (e.g. visiting friends and relatives):

① all of the time ② most of the time ③ more of the time ④ part of the time ⑤ a small part of the time ⑥ no such feeling (weight or score in the order of 1, 2, 3, 4, 5)

Overall health

11. For each of the following questions, which answer best fits your situation?

(1) I seem to get sick more easily than others:

(1) Absolutely true (2) Mostly true (3) Can't be sure (4) Mostly wrong (5) Absolutely wrong `

(Weights or scores in order of 1, 2, 3, 4, 5)

(2) I am as healthy as the people around me:

① absolutely right ② mostly right ③ not sure ④ mostly wrong ⑤ absolutely wrong ` (Weight or score in order of 1, 3, 4)

(Weights or scores in order of 5, 4, 3, 2, 1)

(3) I think my health is getting worse:

① absolutely true ② mostly true ③ not sure ④ mostly false ⑤ absolutely false

(Weights or scores in order of 1, 2, 3, 4, 5)

(4) My health is very good:

① absolutely correct ② mostly correct ③ not sure ④ mostly wrong ⑤ absolutely wrong

(Weight or score in order of 5, 4, 3, 2, 1)

Appendix 3

Hamilton Depression Scale (HAMD)

Hamilton Depression Scale (HAMD) was compiled by Hamilton and is the most commonly used scale for assessing depression in clinical settings. The scale is a 24-item version with a simple methodology and clear criteria that are easy to grasp. It is suitable for adults with depressive symptoms. The total score can better reflect the severity of the disease and can also be a good measure of the effectiveness of treatment, which is the classic and recognized depression assessment scale.

Scale content:

1、 Depressed mood Select the following 1~4 points.

(1) Complained only when asked;.

(2) Expressed spontaneously during the interview.

(3) reveals this emotion without words in expression, posture, voice, or desire to cry; and

(4) The patient's spontaneous speech and non-verbal expression (expression, movement) almost completely shows this emotion.

2、Guilt feeling

(1) Blaming oneself and feeling that one has implicated others.

(2) Thinking that they have committed a crime, or repeatedly thinking about past faults and mistakes; and

(3) Thinking that the present illness, is a punishment for one's mistakes, or having delusions of guilt; and

(4) Guilty delusions accompanied by accusatory or threatening hallucinations.

3、Suicide

(1) Feeling that there is no point in living; and

(2) Wishing that one is already dead, or often thinking about things related to death.

(3) Negative ideas (suicidal thoughts).

(4) Suicidal behavior.

4、Difficulty in falling asleep Initial insomnia.

(1) complaints of difficulty in falling asleep, half an hour after going to bed and still can not fall asleep. (To pay attention to the patient's usual time to fall asleep);.

(2) Complaints of difficulty falling asleep every night.

5、Depth of sleep Mid-range insomnia.

(1) Shallow sleep with many nightmares;.

(2) had woken up in the middle of the night (before 12 o'clock in the evening) (excluding going to the toilet).

6、 Early awakening Final sleep.

(1) There is early awakening, waking up 1h earlier than usual, but can fall back to sleep (usual habits should be excluded);; (2) There is early awakening, waking up 1h earlier than usual, but can fall back to sleep (usual habits should be excluded).

(2) Woke up early and could not fall back to sleep.

7、 Work and interest

(1) Tells only when asked.

(2) Spontaneously expresses, directly or indirectly, a loss of interest in activities, work, or study, such as feeling lethargic, hesitant, unable to persevere, or forcing oneself to work or do activities.

(3) Reduced time or decreased effectiveness of activities, inpatient patients participate in ward labor or recreation less than 3h per day; and

(4) stop working because of the current disease, the hospitalized person does not participate in any activities or without the help of others will not be able to complete the daily things in the hospital room (note that can not be hospitalized on the 4 points).

8、blocked Refers to slow thought and speech, difficulty concentrating, and diminished initiative.

(1) Mild obstruction is found in the psychiatric examination; (2) Mild obstruction is found in the psychiatric examination; (3) Mild obstruction is found in the psychiatric examination.

(2) Significant blockage found on psychiatric examination.

(3) Difficulty in conducting a mental examination; and

(4) Complete inability to answer questions (rigidity).

9、Agitation

(1) Some distraction during examination.

(2) Obviously distracted or much small movements; (3) Unable to answer questions; (4) Completely unable to answer questions (agitation); (5) Unable to answer questions (agitation).

(3) Cannot sit still during the examination had risen;.

(4) Rubbing hands, biting fingers, pulling hair, biting lips.

10、mental anxiety

(1) Complaining when asked.

(2) Spontaneous expression.

(3) Expression and speech revealing obvious worries.

(4) obvious panic.

11、Somatic Anxiety Refers to the physical symptoms of anxiety, including: dry mouth, bloating, diarrhea, hiccups, colic, palpitations, headache, hyperventilation and sighing, as well as frequent urination and sweating.

(1) Mild;.

(2) Moderate, with definite symptoms of the above; and

(3) Severe, where the above symptoms are so severe that they interfere with life or require management.

(4) Severe disruption of life and activities.

12、Gastrointestinal symptoms

(1) Loss of appetite, but eating on their own without encouragement from others; and

(2) Need to be urged or requested to eat and need to apply laxatives or digestive aids.

13、Systemic symptoms

(1) Heaviness in the limbs, back or neck, backache, headache, muscle pain, generalized weakness or fatigue.

(2) Symptoms are obvious.

14、Sexual Symptoms Refers to loss of libido, menstrual disorders, etc..

(1) Mild; (2) Severe.

(2) Severe.

(3) Uncertain, or the item is not suitable for the assessed person (not counted in the total score).

15、Suspiciousness

(1) Excessive concern for the body.

(2) Repeated consideration of health problems.

(3) with paranoid delusions.

(4) hypochondriacal delusions accompanied by hallucinations.

16、Weight loss

(1) According to the history of assessment: ① patients complained of possible weight loss; ② sure weight loss.

(2) according to the weight record assessment: ① weight loss of more than 0.5kg in 1 week; ② weight loss of more than 1kg in 1 week.

17、Self-awareness

(1) Knowing that one is sick, manifested as depression; (2) Knowing that one is sick, manifested as depression; (3) Knowing that one is sick, manifested as depression.

(2) know that they are sick, but blame it on poor food, environmental problems, overwork, viral infections or the need for rest; (2) know that they are sick, but blame it on poor food, environmental problems, overwork, viral infections or the need for rest.

(3) Completely denies having the disease.

Rating Methods:

Most of the HAMD items were rated on a 5-point scale from 0 to 4. The criteria for each level were (0) none; (1) mild; (2) moderate; (3) severe; and (4) very severe. A few items were graded on a 3-point scale from 0 to 2 on the following scale: (0) none; (1) mild to moderate; and (2) severe.

Outcome adjudication:

(1) A total score of more than 24 was classified as severe depression, more than 17 as mild or moderate depression, and less than 7 as no depressive symptoms.

(2) Factor score: HAMD can be categorized into 7 types of factor structure: (i) anxiety somatization: consisting of 5 items such as psychogenic anxiety, somatic anxiety, gastrointestinal symptoms, hypochondriasis, and self-awareness (i.e., items 10, 11, 12, 15, and 17); (ii) weight: i.e., one item of weight loss (item 16); (iii) cognitive disorders: consisting of self-guilt, suicidality, and agitation (items 2, 3, and 9); and (iv) blocking. 4 items such as depressed mood, work and interest, blocking and sexual symptoms (items 1, 7, 8 and 14); ⑤ Sleep disorder: 3 items such as difficulty in falling asleep, lack of deep sleep and early awakening (items 4-6).

Through factor analysis, not only can it specifically reflect the characteristics of the patient's condition, but also the clinical outcomes of the target symptom clusters.

Precautions:

(1) The time frame of the assessment is within the past 1 week.

(2) The examination was conducted by two physicians using conversation and observation, and at the end of the examination, the two raters scored independently.

(3) If used for efficacy assessment, it should be rated 1 time before starting treatment and 1 time after treatment in order to compare efficacy.

(4) In the HAMD, items 8, 9, and 11 are based on observation of the patient; the remaining items are based on the patient's own verbalization; item 1 requires a combination of both. In addition, item 7 needs to be collected from the patient's family or ward staff; item 16 is best assessed on the basis of weight records, or on the basis of the patient's complaints and the information provided by his or her family or ward staff.

Appendix 4　Seattle Angina Scale

1. The degree of restriction of the following activities due to chest pain, tightness in the chest, or angina in the past 4 weeks	
1.1 Dressing yourself	☐1 Severely limited ☐2 Moderately restricted ☐3 Mildly restricted ☐4 slightly restricted ☐5 Unrestricted ☐6 Restricted for other reasons
1.2 Indoor walks	☐1 Severely limited ☐2 Moderately restricted ☐3 Mildly restricted ☐4 slightly restricted ☐5 Unrestricted ☐6 Restricted for other reasons
1.3 Showering	☐1 Severely limited ☐2 Moderately restricted ☐3 Mildly restricted ☐4 slightly restricted ☐5 Unrestricted ☐6 Restricted for other reasons
1.4 Climbing slopes or stairs (three floors, non-stop)	☐1 Severely limited ☐2 Moderately restricted ☐3 Mildly restricted ☐4 slightly restricted ☐5 Unrestricted ☐6 Restricted for other reasons
1.5 Outdoor sports or carrying of miscellaneous objects	☐1 Severely limited ☐2 Moderately restricted ☐3 Mildly restricted ☐4 slightly restricted ☐5 Unrestricted ☐6 Restricted for other reasons
1.6 Brisk walking (1 km)	☐1 Severely limited ☐2 Moderately restricted ☐3 Mildly restricted ☐4 slightly restricted ☐5 Unrestricted ☐0 Restricted for other reasons

1.7 Jogging (1 km)	☐1 Severely limited ☐2 Moderately restricted ☐3 Mildly restricted ☐6 Restricted for other reasons ☐4 slightly restricted ☐5 Unrestricted
1.8 Lifting or moving heavy objects	☐1 Severely limited ☐2 Moderately restricted ☐3 Mildly restricted ☐6 Restricted for other reasons ☐4 slightly restricted ☐5 Unrestricted
1.9 Vigorous exercise (e.g., swimming, (Tennis)	☐1 Severely limited ☐2 Moderately restricted ☐3 Mildly restricted ☐6 Restricted for other reasons ☐4 slightly restricted ☐5 Unrestricted
2. how often you feel chest pain, tightness in the chest, or angina when doing maximal exercise compared to 4 weeks ago<br>☐1 Significant increase☐2 Slight increase☐3 Same☐4 Slight decrease☐5 Significant decrease	
3. Average number of episodes of chest pain, chest tightness and angina pectoris in the past 4 weeks<br>☐1⩾4 times/day☐21-3 times/day☐3⩾3 times/week<br>☐41-2 episodes/week☐5 <1 episode/week☐6 No episodes	
4. Chest pain, chest tightness and angina were the average number of times nitroglycerin was taken in the past 4 weeks.<br>☐1⩾4 times/day☐21-3 times/day☐3⩾3 times/week<br>☐41-2 times/week☐5 <1 time/week☐6 Not used	
5. Trouble complying with medication prescribed for chest pain, tightness in the chest and angina pectoris<br>☐1 Severe☐2 Moderate☐3 Slight☐4 .. Minimal☐5 None☐6 No medication given by doctor	
6. Satisfaction with various measures for the treatment of chest pain, tightness in the chest and angina pectoris	

☐1 Dissatisfied☐2 Mostly dissatisfied☐3 Partly satisfied☐4 Mostly satisfied☐5 Very satisfied
7. Satisfaction with doctor's explanation of chest pain, tightness in the chest and angina pectoris<br><br>☐1 Dissatisfied☐2 Mostly dissatisfied☐3 Partly satisfied☐4 Mostly satisfied☐5 Very satisfied
8. Overall, satisfaction with current treatment for chest pain, tightness in the chest and angina pectoris<br><br>☐1 Dissatisfied☐2 Mostly dissatisfied☐3 Partly satisfied☐4 Mostly satisfied☐5 Very satisfied
9. The extent to which chest pain, tightness in the chest, and angina have interfered with enjoyment of life in the past 4 weeks<br><br>☐1 Severe☐2 Moderate☐3 Slight☐4 .... Minimal☐5 None
10. How would you feel if you still had chest pain, tightness in your chest and angina in your future life?<br><br>☐1 Dissatisfied☐2 Mostly dissatisfied☐3 Partly satisfied☐4 Mostly satisfied☐5 Very satisfied
11. Degree of fear of heart attack and sudden death<br><br>☐1 Always worried☐2 Often worried☐3 Sometimes worried☐4 Rarely worried☐5 Not worried

Appendix 5 Generalized Anxiety Disorder Scale (GAD-7)

I. Introduction

1. Measurement: Self-assessment

2、Scale function: Generalized Anxiety Scale GAD-7 is a widely used clinical scale for assessing anxiety. Regular (1 time/1-2 weeks) self-assessment can be used to observe the trend of anxiety change and treatment effect.

3.Applicable people:All kinds of people.

4. Duration of the assessment: 1-3 minutes

II. Specific tests

Scale Introduction:

Assessment guide: Based on the situation in the past two weeks, please answer whether the following described situation exists and its frequency, please read the questions clearly and choose the option that matches you.

theme				
Feeling nervous, anxious or eager	Not at all.	several days	More than a week	almost every day
Inability to stop or control worry	Not at all.	several days	More than a week	almost every day
Worrying too much about all sorts of things	Not at all.	several days	More than a week	almost every day
It's hard to relax.	Not at all.	several days	More than a week	almost every day
Unable to sit still due to restlessness	Not at all.	several days	More than a week	almost every day
Becoming easily annoyed or impatient	Not at all.	several days	More than a week	almost every day
Fearful as if something terrible is going to happen.	Not at all.	several days	More than a week	almost every day

III. Guidelines for use

1. Scoring method: The Generalized Anxiety Scale GAD-7 has 7 entries. Each entry is divided into 4 levels, which are 3=almost every day; 2=more than a week; 1=several days; 0=not at all, and the total score is the sum of the scores of the 7 entries, and the total score ranges from 0 to 21 points.

Anxiety Level Analysis Scale

totals	analyze
0-4 points	normal level
5-9 points	mild anxiety
10-13 points	moderate anxiety
14-18 points	Moderate to severe anxiety
19-21 points	high anxiety

Appendix 6 Allocation of Acupoints and Non-acupoints.

Acupoint	Location	Non-acupoint	Location
PC6 (Neiguan)	In the anterior region of the forearm, between the tendon of the palmaris longus and the tendon of the radial flexor carpi radialis, 2 cun above the transverse stripe on the distal part of the palmar side of the wrist (about 50 mm).	Non-acupoint 1	In the anterior region of the forearm, at the ulnar end of the tendon of the palmaris longus, 2 cun above the transverse stripe on the distal palmar side of the wrist (about 50 mm). Avoid the hand syncopal meridian, hand taiyin lung meridian, and median nerve.
EX-HN3 (Yintang)	On the forehead of the human body, at the intersection of the line connecting the two eyebrows and the front center line.	Non-acupoint 2	On the forehead of the body, at the same level as the EX-HN3, 0.5 cun (about 15 mm) to the right of the anterior midline, avoiding the Ren and Foot Solar Bladder meridians, as well as avoiding the frontal artery.
KI6 (Zhaohai)	In the human ankle, 1 inch (about 25mm) below the tip of the inner ankle, in the marginal depression of the lower edge of the inner ankle.	Non-acupoint 3	In the human ankle, 2 cun (about 50mm) below the tip of the inner ankle, avoiding the foot syncopal liver meridian and foot shaoyin kidney meridian, as well as avoiding the tibial nerve.
RN17 (Danzhong)	It is located in the anterior midline, on the body of the sternum, at the level of the 4th intercostal space, at the midpoint of the line between the two nipples.	Non-acupoint 4	It is located in the anterior chest of the body, at the same level as RN17, 0.5 cun (about 15 mm) to the right of the anterior median line, avoiding the Ren Chakra, as well as the intercostal nerve.
HT3 (Shaohai)	It is located on the anterior medial side of the forearm, at the midpoint of the line between the transverse elbow stripe and the medial epicondyle of the humerus.	Non-acupoint 5	It is located on the anterior medial side of the forearm, at the midpoint of the line between the transverse elbow stripe and the medial epicondyle of the humerus, 2 cun (about 50 millimeters) above the transverse elbow stripe. Avoiding the Shaoyin Pericardium Meridian and the Taiyin Lung Meridian of the hand, as well as the median nerve and ulnar nerve.
SP6 (Sanyinjiao)	On the medial side of the lower leg, 3 cun (about 75 mm) above the tip of the inner ankle, behind the medial border of the tibia.	Non-acupoint 6	Medial side of the calf, 3 cun (about 75 mm) above the tip of the inner ankle and 1 inch (about 25 mm) behind the medial border of the tibia. Avoid the foot-taiyin spleen meridian and foot-shaoyin kidney meridian. Avoid the area where the tibial nerve and posterior iliac vein run.

Appendix 7 Schematic of the two groups of acupuncture and non-penetrating devices.

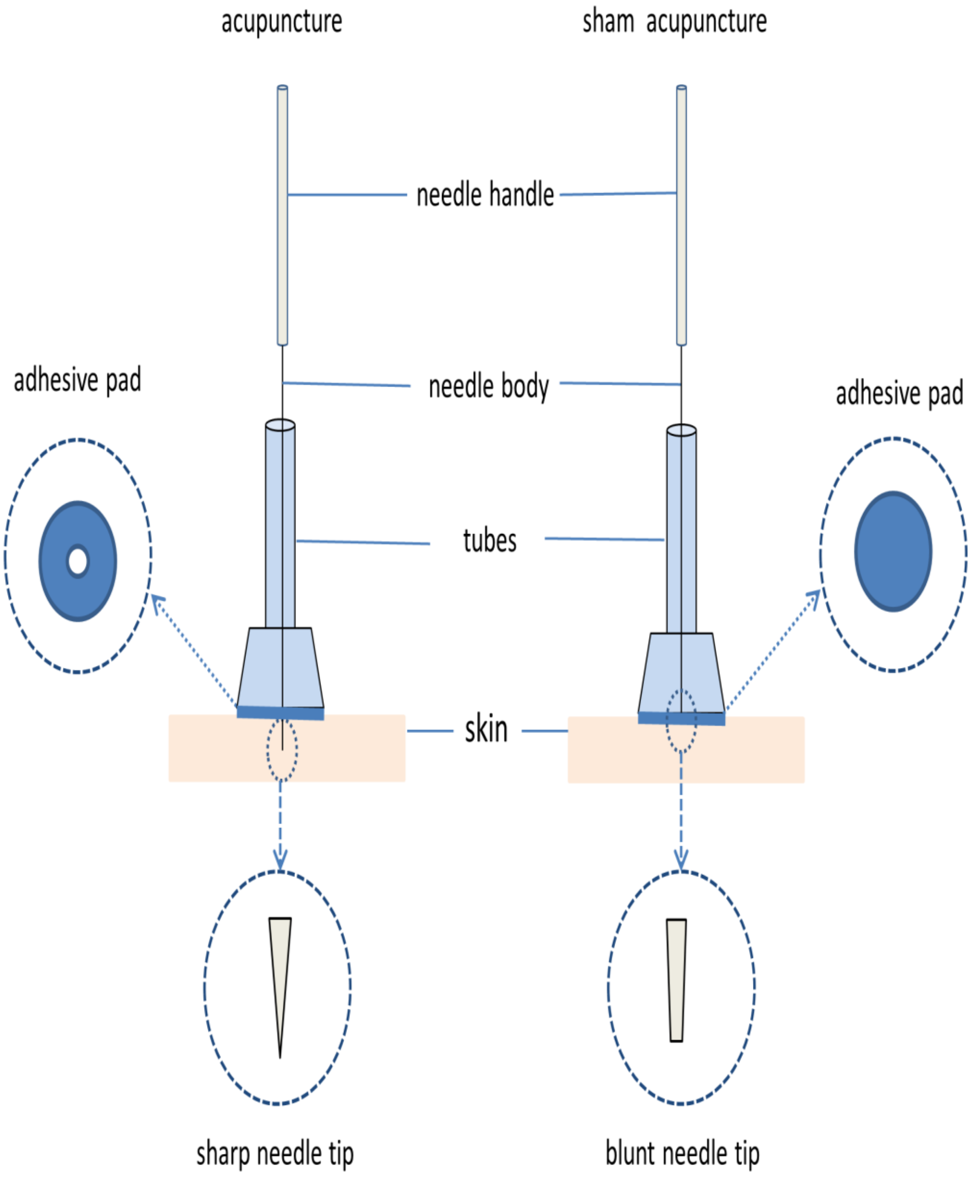

Supplement: S1 File — (PDF) [file pone.0301827.s003.pdf]
