# Supplementary material for: Exploring acupuncture as a treatment for insomnia in perimenopausal women with stable angina pectoris: A protocol for a randomized, double-blind, placebo-controlled clinical trial
Source: PLoS One. 2024 Apr 18;19(4):e0301827. doi: 10.1371/journal.pone.0301827 (PMC11025937; doi:10.1371/journal.pone.0301827)

针灸治疗围绝经期稳定型心绞痛伴失眠：随机、双盲、安慰剂对照临床

试验方案

方案编号	CCZYFYLL2022-029
申办方	长春中医药大学附属医院
主要研究者	邓悦、石锐
版本号	V 2.0
版本日期	2022 年 08 月 26 日

研究者声明及签字页

研究者声明：

我将根据 GCP 规定，认真履行研究者职责。

我已收到研究者手册，我已知晓该研究药物的研究过程，我已被告知将及时收到更新的研究者手册。

我已阅读过此方案，本项研究将根据《赫尔辛基宣言》和中国 GCP 规定的道德、伦理和科学原则进行。我同意按照本方案设计及规定开展此项临床研究。

我将负责做出与临床相关的医疗决定，保证受试者在研究期间出现不良事件时得到及时、适当的治疗，我知道正确报告严重不良事件的要求，我将根据要求记录和报告这些事件。

我保证将数据真实、准确、完整、及时、合法地载入病例报告表。我将接受申办者派遣的监查员或稽查员及药物监督管理部门的稽查和视察，确保临床研究的质量。

我同意研究结果用于药物注册，公开发表。

我将在研究开始前提供一份履历，呈送伦理委员会，并可能呈送管理部门审查。

各临床研究中心主要研究者签名：

研究中心全称：　长春中医药大学附属医院

城市：　吉林省长春市

主要研究者姓名：　石锐，邓悦，　孟文宜，刘兆政，薛文，陈星宇

2022 年　08　月　26　日

目 录

方案摘要......6
试验流程图......9
1.研究背景......10
2.研究目的......11
3.试验设计......11
3.1总体设计......11
3.2随机化程序与分配隐......11
3.3 对照......11
3.4 样本量计算......11
3.5 盲法......11
3.6紧急揭盲......11
3.7疗程的设计......12
4.试验对象......12
4.1 诊断标准......12
4.2 纳入标准......12
4.3 排除标准......12
4.4 退出和终止试验标准......13
5.干预措施......13
5.1 治疗组 13
5.2 对照组......13
5.3允许的治疗......13
6.研究流程和访视评估......14
6.1访视1-筛选......14
6.2基线：访视2......14
6.3 治疗访视：访视3、4......15
6.4治疗访视：访视5......15

6.5 安全随访：访视6、7..16
6.6计划外访视..16
6.7提前退出..16
6.8试验期间的注意事项..17
7. 疗效与安全性评价标准..17
7.1 主要结局..17
7.2次要结局..17
7.3 安全性指标..17
7.4 安全性评价..17
7.4.1不良事件的相关定义及规定..17
7.4.2不良事件记录..18
7.4.3严重程度判断标准..18
7.4.4不良事件与药物因果关系判断..18
7.4.5不良事件的发现、跟踪和随访..18
7.4.6报告程序和处理..19
7.4.7随访未缓解的不良事件..20
8.风险管理和风险评估..20
9.质量控制与质量保证..20
10.数据管理和核查..20
10.1数据管理计划..20
10.2数据核查、清理及质控..21
10.3数据锁定及退出..21
11.统计分析..21
12.伦理学及受试者保护..22
12.1伦理准则..22
12.2伦理委员会..22
12.3知情同意的过程..22

12.4受试者的医疗和保护..23
12.5受试者隐私的保护..23
12.6招募受试者..23
13.质量保证和质量控制..23
13.1 主观症状评价质量控制..24
13.2 实验室检查指标质量控制..24
13.3 临床监查/稽查质量控制..24
14.资料保存..24
15.参考文献..24
附件1 匹兹堡睡眠质量指数..27
附件2 SF-36生活质量表调查表..28
附表3 汉密尔顿抑郁量表(HAMD)..32
附件4 西雅图心绞痛量表..36
附件 5 广泛性焦虑量表(GAD-7)..38
附录 6 穴位和非穴位的分配..40
附录 7 针灸和非穿刺器械两组示意图..41

方案摘要

试验题目	针灸治疗围绝经期稳定型心绞痛伴失眠：随机、双盲、安慰剂对照试验
申办单位	长春中医药大学附属医院
试验目的	评估针灸治疗伴有稳定性心绞痛和失眠的围绝经期受试者的有效性和安全性。
目标病例数	治疗组和对照组各55例，合计110例
试验分组	治疗组：手工针刺<br>对照组：假针灸组
试验设计	单中心、随机、双盲、安慰剂对照临床试验设计。
样本量\统计设计	根据既往的临床研究结果，针灸干预方案结束后的匹兹堡睡眠质量指数（PSQI）得分被用来确定所需的样本量。统计显著性定义为$\alpha=0.05$，功率为 90%。根据之前的一项研究，围绝经期女性失眠患者在接受假针灸治疗后的 PSQI 平均分（$\bar{X}\pm SD$）为 11.43±2.11，在接受人工针灸治疗后的 PSQI 平均分（$\bar{X}\pm SD$）为 9.20±3.24。使用样本量公式$n=2\times[((Z_\alpha+Z_\beta)\times\sigma)/\delta]\hat{}2$ 使用较大的标准差 3.24，$\delta=11.43-9.20=2.23$，$\beta=0.1$，$1-\beta=0.9$，$\alpha=0.05$。因此，每组所需的样本量为 44 个。考虑到 20% 的随访损失，该研究需要的样本总数为 110 人。
研究对象	纳入标准<br>①符合上述稳定型心绞痛和失眠的诊断标准；<br>②女性，45-55 岁，处于围绝经期；<br>③入组前至少 6 个月未接受针灸治疗；<br>④提供知情同意书并同意随机分组。<br>排除标准<br>①未处于围绝经期；<br>②严重的肾、肝、血液或其他系统疾病；<br>③精神失常或意识障碍；<br>④未控制的严重高血压、糖尿病或恶性心律失常；<br>⑤瓣膜疾病、近期急性心肌梗死、不稳定型心绞痛、心肌炎、肥厚型心肌病

	或起搏器术后严重心力衰竭（NYHA 分级≥III）；<br>⑥过去 3 个月内进行过大手术、外伤、出血事件或严重感染；<br>⑦因药物、酒精或环境因素导致的失眠；<br>⑧穴位附近有局部皮肤感染；<br>⑨恶性肿瘤或甲状腺功能亢进<br>⑩晕厥史或严重的针刺恐惧症；<br>⑪研究者认为可能影响参与或完成研究的任何其他情况。
干预措施	参与者将在12周内接受36次针灸治疗，频率为每周3次。
研究周期	主要和次要结果将在基线、4周、8周、12周(干预完成后)以及额外的12周随访时进行评估。
中止/退出标准	当试验中发生严重的安全性问题，为保护受试者权益，或因经费原因、管理原因等，研究者、伦理委员会、申办者、卫生行政管理部门可进行终止试验。受试者不愿意或不可能继续进行临床试验，向研究者提出退出试验。研究过程中出现的任何不良事件;合并症、并发症或妨碍继续参与的生理改变;治疗依从性差;干预禁忌症;失诊;揭盲事件。
理化检查	包括c反应蛋白(CRP)、脂蛋白相关磷脂酶A2 (Lp-PLA2)、心脏脂肪酸结合蛋白水平(C-FABP)、血常规（WBC、RBC、HGB、PLT）、尿常规、肝功能(ALT、AST)、肾功能(Scr、BUN）、十二导联心电图。
体格检查	①体温；②安静时心率；③呼吸；④血压。
有效性评价	主要评价指标：<br>匹兹堡睡眠质量指数(PSQI)<br>次要评价指标：<br>①稳定性心绞痛(SAP)的评估:<br>②使用36项简短健康调查(SF-36)进行生活质量评估。<br>③安眠药的剂量。<br>④汉密尔顿抑郁评定量表(HAMD)。<br>⑤广泛性焦虑障碍7项(GAD-7)量表。

安全性评价	①生命体征(体温、血压、呼吸频率、心率);<br>②血尿常规检查;<br>③肝肾功能检查;<br>④心电图。
统计分析	统计分析将使用 SPSS 24.0 进行。连续变量将以平均值 ± 标准差或中位数（四分位数间距）表示。组间比较将采用双样本 t 检验，或对偏斜数据采用非参数检验。组内基线到终点的变化将通过配对 t 检验进行分析。分类变量将使用费雪精确检验或卡方检验进行比较。统计显著性定义为 $p<0.05$。

试验流程图

	研究期						
	招募	分配	治疗期			随访期	
**时间节点（周）**	**-1**	**0**	**4**	**8**	**12**	**16**	**24**
注册：	X						
资格筛选	X						
知情同意书		X					
分配		X					
人口统计学		X					
生命体征		X	X	X	X	X	X
干预：							
手针			◆——	——	——◆		
假针灸			◆——	——	——◆		
评估：							
PSQI		X	X	X	X	X	X
CRP		X			X		
脂蛋白-PLA2		X			X		
C-FABP		X			X		
SAQ		X	X	X	X	X	X
SF-36		X	X	X	X	X	X
安眠药剂量		X	X	X	X	X	X
HAMD		X	X	X	X	X	X
GAD-7		X	X	X	X	X	X
心电图		X	X	X	X		X
实验室检查		X			X		
不良反应			X	X	X	X	X

1.研究背景

稳定型心绞痛（SAP）是冠状动脉疾病的一种常见表现，对患者的生活质量造成严重影响，并预示着发展为急性冠状动脉综合征的显著风险[1-3]。近年来，随着众多冠心病患者出现严重的睡眠障碍，SAP 合并失眠症引起了更多关注[4,5]。由于雌激素水平急剧下降，围绝经期妇女更容易患冠心病和失眠症[6,7]。

心绞痛的发生可能导致失眠，而失眠会对情绪调节、认知、记忆和全身免疫力产生不利影响，同时还会造成包括心血管系统在内的多器官损伤 [8,9]。

Sivertsen 等人在一项长达 11 年的纵向研究中发现，失眠是心绞痛、脑血管意外和高血压的显著危险因素[10]。虽然中国的睡眠医学研究始于 20 世纪 80 年代，但正规化的学科仍处于起步阶段，非专业人士对睡眠卫生的认识仍微乎其微，约 43% 的中国人存在不同程度的睡眠障碍，其中女性患者比例更高[11, 12]。睡眠障碍导致的医疗支出激增和工作效率下降已成为迫在眉睫的公共卫生问题[13]。

传统的物理治疗模式、行为治疗策略和心理治疗干预需要多方面的专业人员和物质装备，涉及多种决定因素的复杂相互作用，甚至包括危险的补救后遗症。这种综合范例在寻求被临床普遍采用的过程中遇到了巨大的障碍。药物干预作为一种普遍采用的方式，在治疗领域投下了显著的阴影。其中，镇静剂是临床实践中名副其实的基石。然而，长期用药会使患者面临药物依赖、反作用复发、强迫性入迷等各种问题，同时还会产生各种不良后遗症，停药后出现戒断症状更是令人担忧[14]。近年来，针灸的临床疗效得到越来越多的认可，多项研究阐明了针灸对失眠症的治疗作用[15-17]。针灸可通过促进慢波睡眠提高睡眠质量[18,19]，对治疗围绝经期失眠症也有疗效[20-23]。

最近的调查表明，2019 年新型冠状病毒（2019-nCoV）诱发的肺炎与心绞痛和失眠的易感性升高之间存在密切联系[24,25]。虽然过去曾对针灸治疗失眠的疗效进行过相关评估，但 2019-nCoV 大流行的爆发导致目前的随机对照试验分析明显不足，尤其是关于围绝经期妇女同时心绞痛和失眠的专门指征。鉴于评估针灸治疗稳定型心绞痛围绝经期患者失眠的疗效和安全性的数据极少，我们开展了这项研究。

2. 研究目的

本研究方案的主要目的是评估针灸对同时患有稳定型心绞痛和失眠症的围绝经期受试者的有效性和安全性。

3.试验设计

3.1 总体设计

本试验为单中心、随机、双盲、安慰剂对照临床试验。

3.2 随机化程序与分配隐藏

研究协调员将在不联系本研究参与者的情况下，利用 SPSS 24.0 软件独立生成随机化编号。为了确保分配的隐蔽性，将使用按顺序编号的不透明密封信封，并由指定人员独立管理随机化顺序。

随机表中随机号格式为 R+三位阿拉伯数字，从 R001 依次编写。筛选成功的受试者按照筛选号从小到大获得随机号，在试验过程中使用对应编号的试验治疗。试验过程中，因任何原因、不论是否使用了手针治疗或安慰剂，退出临床试验的随机化受试者，将保留其随机号，该受试者将不被允许再次进入本试验，且不再替补。

3.3 对照

采用安慰剂(假针灸组)对照。

3.4 样本量计算

针灸干预方案结束后的匹兹堡睡眠质量指数（PSQI）得分被用来确定所需的样本量。统计显著性定义为 α=0.05，功率为 90%。根据之前的一项研究，围绝经期女性失眠患者在接受假针灸治疗后的 PSQI 平均分（$\bar{x}$±SD）为 11.43±2.11，在接受人工针灸治疗后的 PSQI 平均分（$\bar{x}$±SD）为 9.20±3.24[30]。使用样本量公式 n=2×[((Z_α+Z_β)×σ)/δ]^2 使用较大的标准差 3.24，δ = 11.43 - 9.20 = 2.23，β = 0.1，1-β = 0.9，α = 0.05。因此，每组所需的样本量为 44 个。考虑到 20% 的随访损失，该研究需要的样本总数为 110 人。

3.5 盲法

将对患者、评估人员和数据分析人员隐瞒组别分配情况。两组的针灸穴位和技术具有可比性。对照组将使用模仿真针外观的非穿刺装置进行假针灸，以保持盲法。为了评估盲法是否成功，我们将调查参与者在干预后对接受真针灸还是假针灸的看法。由于针灸的特殊性，对针灸师进行盲法操作是不可行的。

3.6 紧急揭盲

紧急情况下，研究者认为知晓受试者所接受的治疗有利于不良事件的处理时，可破盲。紧急破盲时，由该研究获得授权的人员来进行紧急破盲操作，并在受试者的研究病历、eCRF 和任何

相应的 AE 报告中详细记录破盲的原因、时间和地点并签字（除负责研究者外，同时应至少有另外一位见证人的签字），紧急破盲后的 24 小时之内，研究者必须通知申办单位，同时及时通知主要研究者、临床监查员。受试者紧急破盲后应停用研究药物，该受试者中止试验，作脱落病例处理。

3.7 疗程的设计

①设计导入期目的为消除已服用类似治疗的延迟作用、稳定基线水平、去除安慰剂效应以及充分确定纳入标准和排除标准，培养受试者依从性。本试验设置 7 天的导入期。

②治疗期的设计

本研究治疗期 共 12 周

③随访期的设计

本研究随访期共 12 周。

4. 试验对象

4.1 诊断标准

①稳定型心绞痛的诊断遵循《2019 年 ESC 慢性冠状动脉综合征诊断和治疗指南》。冠状动脉疾病通过冠状动脉造影术、CT 血管造影术或跑步机运动测试阳性确定。患者表现为加拿大心血管协会 II-III 级心绞痛，稳定型心绞痛病史≥30 天，每周心绞痛发作 2-5 次。

②根据《国际睡眠障碍分类》，失眠的定义为匹兹堡睡眠质量指数大于 5。入院时由两名独立的盲人评估员进行失眠持续时间和频率的问卷调查，并进行共识分析，以确保结果的一致性。所有入选患者自述失眠持续时间至少为 30 天，每周失眠次数≥3 次。

4.2 纳入标准

①符合上述稳定型心绞痛和失眠的诊断标准；

②女性，45-55 岁，处于围绝经期；

③入组前至少 6 个月未接受针灸治疗；

④提供知情同意书并同意随机分组。

4.3 排除标准

①未处于围绝经期；

②严重的肾、肝、血液或其他系统疾病；

③精神失常或意识障碍；

④未控制的严重高血压、糖尿病或恶性心律失常；

⑤瓣膜疾病、近期急性心肌梗死、不稳定型心绞痛、心肌炎、肥厚型心肌病或起搏器术后严重心力衰竭（NYHA 分级≥III）；

⑥过去 3 个月内进行过大手术、外伤、出血事件或严重感染；

⑦因药物、酒精或环境因素导致的失眠；

⑧穴位附近有局部皮肤感染；

⑨恶性肿瘤或甲状腺功能亢进

⑩晕厥史或严重的针刺恐惧症；

⑪研究者认为可能影响参与或完成研究的任何其他情况。

4.4 退出和终止试验标准

根据保障患者自主权的伦理要求，受试者有权在任何时候以任何理由退出研究。对于选择退出的受试者，必须详细记录其退出的理由，并将其作为停止试验的评估指标。临床研究者应认真记录终止试验的依据及其与研究的相关性，并分析终点事件对研究结论的潜在影响。

所有关于研究退出和终止的主要数据将保持密封，以便在试验完成后进行检查。此外，终止试验的理由还包括：研究期间出现的任何不良事件；合并症、并发症或生理改变导致无法继续参与；治疗依从性差；禁忌干预；漏诊；以及解除约束事件。

5.干预措施

参与者将在 12 周内接受 36 次针灸治疗，频率为每周 3 次。针灸只能由持有执照的针灸师进行。他们必须接受标准化技术培训，包括针刺操作和穴位定位。

5.1 治疗组

使用直径 0.30 毫米、长 40 毫米的无菌针灸针（苏州华伦医疗器械有限公司）。穴位的确定和定位符合世界卫生组织的标准。处方穴位为 PC6（内关）、EX-HN3（印堂）、KI6（照海）、RN17（膻中）、HT3（少海）和 SP6（三阴交）。EX-HN3 和 RN17 的插入深度为 5-10 毫米，而 KI6、PC6、HT3 和 SP6 的插入深度为 20-25 毫米。针刺过程包括：消毒皮肤和器械；在穴位上进针，以 60-90 转/分钟的速度在 90°-180° 之间双向旋转，直至出现麻、胀、酸、痛等德奇感觉，并使用中国改良的麻省总医院针刺感觉量表（C-MASS）进行量化。每个针灸疗程为 30 分钟，每 10 分钟手动刺激 1 分钟。

5.2 对照组

假针灸组在进行与治疗组相同的消毒程序后，使用直径为 0.30 毫米、长度为 40 毫米的钝头针（华伦医疗器械有限公司，苏州）在非穴位上进行非穿刺性针刺（所选刺激点靠近治疗组的穴位，但在经络之外，避开人体神经分布）。具体的非穴位选择见附件 6。治疗组的治疗时间与治疗组相同，但钝针（附件 7）仅轻叩皮肤表面，未穿透组织或操作以激发出气。在 30 分钟的治疗过程中，每隔 10 分钟模拟 1 分钟的假针刺激。

5.3 允许的治疗

①受试者在参加试验前已经使用，用于治疗合并疾病的药品，可以继续使用，在试验期间尽量保持原有的药品种类、用法用量不变。

②受试者心绞痛发作时，可含服统一发放的硝酸甘油，并详细记录。

③试验期间如受试者发生不良事件，可依据相关指南使用相应药品进行治疗，但研究者需评估所用药品是否对于试验用药物疗效或安全性评价造成影响，并酌情判断受试者是否在采用相应治疗后需要退出本试验。

④试验期间服用的药品或其他治疗，需要在研究者指导下使用。试验期间所使用的任何药品，必须在研究病历中详细记录并加以说明，包括药品名称、使用方法、药品剂量、使用原因等，以便总结时加以分析和报告。

6. 研究流程和访视评估

6.1 访视 1-筛选（-7~0 天）

- ✓ 签署知情同意书
- ✓ 入排标准
- ✓ 人口统计学
- ✓ 年龄
- ✓ 性别
- ✓ 民族
- ✓ 身高
- ✓ 体重
- ✓ 既往病史和合并症
- ✓ 伴随疾病、伴随治疗/用药
- ✓ 体格检查
- ✓ 生命体征
- ✓ PSQI量表评分
- ✓ 西雅图心绞痛量表评分
- ✓ 安眠药物使用情况
- ✓ 心电图检查

6.2 基线：访视 2（第 0 周，随机当日）

- ✓ 入排标准
- ✓ 伴随疾病、伴随治疗/用药
- ✓ 体格检查

- ✓ 生命体征
- ✓ PSQI量表评分
- ✓ 西雅图心绞痛量表评分
- ✓ 汉密尔顿抑郁量表（HAMD）
- ✓ 广泛性焦虑症量表（GAD-7）
- ✓ 健康相关生活质量问卷（SF-36）
- ✓ 安眠药物使用情况
- ✓ 心电图检查
- ✓ 随机化
- ✓ 针刺部位观察
- ✓ 实验室检查（血常规、血生化、CRP，C 反应蛋白；Lp-PLA2，脂蛋白相关磷脂酶 A2；C-FABP，心脏脂肪酸结合蛋白水平；）

6.3 治疗访视：访视 3、4（治疗第 4 周±2 天、第 8 周±2 天）

- ✓ 伴随疾病、伴随治疗/用药
- ✓ 体格检查
- ✓ 生命体征
- ✓ PSQI量表评分
- ✓ 西雅图心绞痛量表评分
- ✓ 汉密尔顿抑郁量表（HAMD）
- ✓ 广泛性焦虑症量表（GAD-7）
- ✓ 健康相关生活质量问卷（SF-36）
- ✓ 安眠药物使用情况
- ✓ 心电图检查
- ✓ 针刺部位观察
- ✓ 不良事件评估

6.4 治疗访视：访视 5（治疗第 12 周±2 天）

- ✓ 伴随疾病、伴随治疗/用药
- ✓ 体格检查
- ✓ 生命体征
- ✓ PSQI量表评分（预计用时20分钟）
- ✓ 西雅图心绞痛量表评分（预计用时15分钟）

- ✓ 汉密尔顿抑郁量表（HAMD）（预计用时15分钟）
- ✓ 广泛性焦虑症量表（GAD-7）（预计用时15分钟）
- ✓ 健康相关生活质量问卷（SF-36）（预计用时15分钟）
- ✓ 安眠药物使用情况
- ✓ 心电图检查
- ✓ 针刺部位观察
- ✓ 实验室检查（血常规、血生化、CRP，C 反应蛋白；Lp-PLA2，脂蛋白相关磷脂酶 A2；C-FABP，心脏脂肪酸结合蛋白水平；）
- ✓ 不良事件评估

6.5 安全随访：访视 6、7（第 16 周±2 天、第 24 周±2 天）

- ✓ 伴随疾病、伴随治疗/用药
- ✓ 体格检查
- ✓ 生命体征
- ✓ PSQI量表评分
- ✓ 西雅图心绞痛量表评分
- ✓ 汉密尔顿抑郁量表（HAMD）
- ✓ 广泛性焦虑症量表（GAD-7）
- ✓ 健康相关生活质量问卷（SF-36）
- ✓ 安眠药物使用情况
- ✓ 心电图检查
- ✓ 针刺部位观察
- ✓ 不良事件评估

6.6 计划外访视

出于对受试者安全的考虑，研究者可以要求受试者进行额外的访视或检查。计划外的访视或检查结果均需记录在原始病历和病例报告表中。

6.7 提前退出

当受试者因各种原因决定退出临床试验时,为了保障其安全,研究者需与受试者进行沟通,尽量说服其完成当期访视所需的相关检查和评估内容。

如果受试者坚持拒绝进行当期访视,那么以受试者退出前最后一次的相关信息作为提前中止访视的数据,用于有效性和安全性评价。对于提前退出的受试者,研究者应当详细记录其退出的原因、退出日期等相关信息,完整地记录在研究病历中。

6.8 试验期间的注意事项

①研究者指导受试者在研究期间注意下列事项；

②试验期间避免刺激性食物、避免饮酒；

③保持稳定的饮食、作息及生活习惯；

④避免情绪波动，平时要保持心情舒畅，避免精神刺激，解除各种精神压力。

⑤试验期间可适量运动，尽量避免剧烈运动和长时间劳累；

7.疗效与安全性评价标准

7.1 主要结局

匹兹堡睡眠质量指数(PSQI)由 19 个自评项目和 5 个异评项目组成，将用于评估过去一个月全球睡眠质量的主要结果，包括 7 个组成部分:睡眠持续时间、睡眠潜伏期、睡眠效率、睡眠药物使用、睡眠障碍、主观睡眠质量和睡眠质量日间功能障碍。PSQI 将在基线、每个 4 周治疗期(第 4、8、12 周)后以及第 16 和 24 周的随访期间进行评估。

7.2 次要结局

①稳定性心绞痛(SAP)的评估:在基线、第 4 周、第 8 周和第 12 周使用西雅图心绞痛问卷(SAQ)评估 SAP，量化治疗前后的心绞痛症状。SAP 血液生物标志物:两组患者在治疗开始和结束时均从肘窝采集空腹静脉血样本。c 反应蛋白(CRP)、脂蛋白相关磷脂酶 A2 (Lp-PLA2)和心脏脂肪酸结合蛋白(C-FABP)的水平由独立实验室进行盲法分组检测。

②使用 36 项简短健康调查(SF-36)进行生活质量评估。

③安眠药的剂量。

④汉密尔顿抑郁评定量表(HAMD)。

⑤广泛性焦虑障碍 7 项(GAD-7)量表。

7.3 安全性指标

①生命体征(体温、血压、呼吸频率、心率);

②血尿常规检查;

③肝肾功能检查;

④心电图。

⑤不良事件。

7.4 安全性评价

7.4.1 不良事件的相关定义及规定

①不良事件的相关定义及规定：不良事件（Adverse Event，AE）是指受试者接受试验用药品后出现的所有不良医学事件，可以表现为症状体征、疾病或者实验室检查异常，但不一定与试验用

药品有因果关系。不良事件可以是任何无益或非期望的体征（包括异常实验室检测值）、症状或在药物使用期间伴随发生的暂时疾病，无论这种疾病是否与药物有关。

②严重不良事件 （Serious Adverse Event，SAE）指受试者接受试验用药品后出现死亡、危及生命、永久或者严重的残疾或者功能丧失、受试者需要住院治疗或者延长住院时间，以及先天性异常或者出生缺陷等不良医学事件。

③可疑且非预期严重不良反应（Suspicious and Unexpected Serious Adverse Reactions，SUSAR)，指临床表现的性质和严重程度超出了试验药物研究者手册、已上市药品的说明书或者产品特性摘要等已有资料信息的可疑并且非预期的严重不良反应。

7.4.2 不良事件记录

试验期间如实填写不良事件记录表，记录不良事件的发生事件、严重程度、持续时间、采取的有效措施和转归等内容。

7.4.3 严重程度判断标准

试验过程中发生的所有不良事件均依据以下标准进行严重程度判定。

I 级：轻度；无症状或轻度症状；仅临床或诊断发现；无需治疗；

II 级：中度；最小的、局部的或非侵入性治疗指征；年龄相关工具性日常生活活动受限；

III 级：重度或重要医学意义，但不会立即危及生命；住院治疗或延长住院时间指征； 致残；自理性日常生活活动受限；

IV 级：危及生命，需紧急治疗；

V 级：死亡。

7.4.4 不良事件与药物因果关系判断

不良事件与治疗相关性的分析需综合考虑以下因素：

①治疗开始时间与不良事件发生时间的先后顺序,是否存在明确的因果关系。

②不良事件的表现是否符合该种治疗已知的不良反应特征。

③不良事件是否可能由合并用药、患者的临床状况或其他治疗方式所导致。

④在停止治疗后,不良事件是否减轻或消失。

⑤重新进行治疗时,是否会再次出现同样的不良反应。

7.4.5 不良事件的发现、跟踪和随访

不良事件将通过口头询问的方式获取并记录在研究病历中。为确保评估的一致性,研究者或指定人员在每次询问时,都应使用相同的问题措辞,避免引起受试者间的差异。

研究者还需要询问受试者之前尚未解决的任何不良事件情况。研究者需要评估不良事件的严重程度、强度、与研究用药的相关性,以及所采取的处理措施。在每次回访或相应的终止访视时,

研究者都必须向受试者询问上述问题。

参与临床试验的各实验室应按照标准操作程序和质量控制程序进行检测。参与单位应提供其"实验室检查正常值范围",如有变更,需及时更新并补充说明。

所有不良事件均应跟踪,直至得到恰当解决或病情稳定为止。以实验室检测值为依据的不良事件,应追踪实验室结果至恢复正常或保持稳定,并在研究病历中完整记录处理过程、随访数据及转归情况,附上相关检查报告单。

7.4.6 报告程序和处理

①不良事件的报告程序和处理

发生任何不良事件，如受试者的主观不适及实验室检测异常，均需认真对待，仔细分析，立即采取措施保护受试者的安全。并将不良事件的详细情况记录于研究病历中，并根据情况记录其持续、转归消失等情况。

②严重不良事件的报告程序和处理

发生严重不良事件(SAE)时,研究人员必须立即为受试者采取适当、积极的抢救和保护措施,尽最大努力确保受试者安全,并根据病情决定是否终止试验。研究者应收集 SAE 相关资料,如医疗记录和检查结果,以准确、及时填写 SAE 报告,并在 24 小时内以《严重不良事件报告表》的形式向申办方、临床试验机构办公室、伦理委员会报告。

所有 SAE 均需由研究者进行跟踪。跟踪情况应由研究者在《严重不良事件报告表》/《严重不良事件随访报告》中详细记录,并在 24 小时内报告给申办方、临床试验机构办公室、伦理委员会。跟踪频率由研究者根据 SAE 具体情况决定,以确保及时获取最新信息,直至出现最终结果。一旦 SAE 症状消失或达到稳定状态,或受试者失访、死亡,研究者将总结书面报告提交申办方,同时报告伦理委员会及机构。

如 SAE 为死亡事件,研究者应向申办者、伦理委员会及药物临床试验机构提供其他所需资料,如尸检报告和最终医学报告。书面报告时,应注明受试者在临床试验中的识别代码;报告资料应确保内容完整、准确,为申办者评估提供依据。

研究病历及电子病例报告表(eCRF)中的相关信息,需与 SAE 报告表及原始资料中的记录保持一致。SAE 报告表需由研究者签字及注明日期,并妥善保存在临床试验机构。

在临床试验过程中,如果受试者发生严重不良事件或不良反应,为了适当的处理和及时救治,需要了解受试者所服用的药物组别。在这种紧急情况下,主要研究者有权决定紧急揭盲,并详细记录揭盲的原因、时间、地点,并签字留档。揭盲后应及时通知申办方和临床监查员。一旦揭盲,该受试者将被终止试验,并作脱落病例处理,同时需将处理结果通知监查员,并在原始研究病历中详细说明理由、日期并签字确认。

7.4.7 随访未缓解的不良事件

所有不良事件都应当及时记录、追踪，直到得到妥善解决或病情稳定。对治疗后理化检查出现的异常指标应及时复查至正常或稳定。

8. 风险管理和风险评估

①在筛选受试者时,必须严格遵循纳入和排除标准,确保符合要求。

②知情同意书应当清楚告知受试者参与本研究可能存在的风险,让其了解相关情况。

③在研究开展之前,需检查急救药品是否齐全、有效期是否仍在有效期内,并在研究过程中定期核查,确保急救药品的可用性。

④研究开始之前,应制定完善的应急预案,并与医院重症监护病房建立受试者转移和护理的联系程序,以便在必要时及时作出响应。

⑤应建立研究者与实验室之间的沟通和交流机制,确保对于临床实验室危急值能够及时沟通和处理,避免延误。

9. 质量控制与质量保证

①质量控制措施

研究者应采用标准操作规程，以保证临床试验的质量控制和质量保证系统的实施。临床试验中所有观察结果和发现都应加以核实，以保证数据的可靠性，确保临床试验中各项结论来源于原始数据。在数据处理的每一阶段必须采用质量控制，以保证所有数据可靠，处理正确。

②研究者的培训

临床试验开始前，监查员协同各试验中心负责人应对研究者进行试验方案的培训，以让研究者了解并熟悉治疗的性质、作用、疗效及安全性，同时也应掌握临床试验进行期间发现的所有与该药物有关的新信息。

③提高受试者的依从性

研究者应认真执行知情同意，使受试者充分理解试验要求，配合试验。研究者还应要求患者在随访时必须将正在使用的所有药物带来，以检查患者的合并用药，并在病例报告表上记录，对于疗效较差的和不能按时用药的患者，应当加强随访。

④临床试验的监查

由主要研究者任命监查员，定期进行试验医院现场监查访问，以保证研究方案的所有内容都得到严格遵守，并对原始资料进行检查以确保与 CRF 上的内容一致。

10. 数据管理和核查

10.1 数据管理计划

数据管理经理根据 GCP 相关原则和临床试验相关内容（如方案、CRF、项目实际情况等）制

定数据管理计划。数据管理计划将记录、描述和定义数据管理的各项工作，以此指导整个数据管理过程。数据管理计划应包含：数据库建立、数据录入、数据核查、质疑表管理、医学编码、数据库锁定、数据保存等内容，并规定数据管理的一些时间点和明确相关人员的职责。在干预期间，研究数据通过纸质病例报告表的电子转录收集并按方案记录，并在每次研究访问后 1 周内使用 EpiData 软件输入数据，实现逻辑验证和带验证的双数据录入，以确保准确性。研究外部的独立数据管理人员监督数据输入、验证、安全性和超出临床预期范围的异常值的解决。最后，在研究发起人、首席研究员、生物统计学家和数据管理员签字后，数据库被锁定以供分析，不允许进一步修改。

10.2 数据核查、清理及质控

①数据核查

数据核查计划制定是由数据管理统计部门、医学技术部门以及项目经理在充分掌握方案设计的前提下依据方案的数据管理内容全部核查点进行具体讨论确定。在执行过程中如需修改，则由数据管理员修订后进行版本更新，并再次由上述各部门共同签字确认。

②数据清理

数据核查过程中的疑问，是以在线系统自动生成的疑问及人工发出的疑问两种形式产生，由研究者进行在线答疑，直至数据清理干净。最终疑问均由研究者确认签字。数据管理部门保存疑问表电子版。

③数据质控

实时编辑核查的运用，出现任何不符合质控或质检要求时，需要采取的行动要求和跟踪程序进行记录并修正。在实施质控过程中，应对不符合规范的数据质量进行列表。这一部分是动态纪录，需要数据管理员，监查员或稽查员根据实际发现的错误百分率列表予以总结并会议传达。

10.3 数据锁定及退出

完成数据锁库清单，依据数据库锁定的程序，由数据管理人员、统计分析人员、临床监查员代表、研究者等签署书面批准数据库锁定文件，由数据管理员将其导出指定格式的数据库，交与统计人员进行统计分析。数据锁定后如有确切证据证明有必要解锁，研究者及相关人员需签署解锁文件。

11. 统计分析

统计分析将使用 SPSS 24.0 进行。连续变量将以平均值 ± 标准差或中位数（四分位数间距）表示。组间比较将采用双样本 t 检验，或对偏斜数据采用非参数检验。组内基线到终点的变化将通过配对 t 检验进行分析。分类变量将使用费雪精确检验或卡方检验进行比较。统计显著性定义为 $p<0.05$。

12.伦理学及受试者保护

12.1 伦理准则

本次研究将根据《赫尔辛基宣言》道德原则进行，并遵循《临床试验质量管理规范》（GCP）、适用法规要求政策。

12.2 伦理委员会

临床试验方案需由主要研究者与申办方经过充分商议后共同制定。在临床试验开始之前,研究者必须获得伦理委员会的书面批准,未获批准前不得开始筛选受试者。如果在实施过程中需要对方案进行修订,也须再次提交伦理委员会审批,获得批准后方可实施修订内容。

在试验过程中,如果研究者获得了可能影响受试者继续参加的新信息,应以知情同意书等书面形式及时告知受试者或其法定代理人。所有需告知受试者的新信息,在获得伦理委员会批准后,受试者应当再次签署知情同意书,以示知晓并同意。新入选的受试者则需签署更新后的知情同意书和其他相关文件资料。

12.3 知情同意的过程

研究中心的主要研究者（或其指定代表）将：

①在知情同意过程中,研究者必须遵循临床试验质量管理规范和赫尔辛基宣言的伦理准则,不得以任何不正当手段如胁迫、利诱等影响受试者的自主决定。

②所有与试验相关的书面或口头信息,均不得含有剥夺受试者及其法定代理人合法权益的语言,也不得免除研究者、医疗机构、申办方及代理机构的应尽责任。

③研究者或指定人员应全面告知受试者有关试验的所有相关事宜,包括书面资料和伦理委员会批准意见。无表达知情同意能力的受试者,应由其法定代理人代为作出决定。

④提供给受试者的知情同意书等口头和书面资料,应采用通俗易懂的语言和表达方式,便于受试者或其法定代理人、见证人理解。

⑤签署知情同意前,应给予受试者或法定代理人充足时间和机会了解试验详情,并解答所有相关问题。

⑥受试者或法定代理人及执行知情同意的研究者,应在知情同意书上分别签名并注明日期。

⑦如受试者或法定代理人均缺乏阅读能力,须有公正见证人协助和见证知情同意过程。研究者应详细解释知情同意书及其他文字资料内容。受试者或法定代理人口头同意并签署后,见证人也须在知情同意书上签字并注明日期,以证明受试者或法定代理人已获准确解释并理解相关内容,自愿参加。

⑧受试者或法定代理人应获得一份已签署的知情同意书副本及其他书面资料;试验期间也应获得更新版知情同意书及修订文本。

⑨当法定代理人代为知情同意时,应尽量让受试者了解相关信息,并尽可能由受试者亲自签署知情同意书。

⑩在紧急情况下,如未能事先获得受试者知情同意时,必须取得法定代理人同意。如受试者及其法定代理人均不在场,入选方式应在试验方案及文件中明确说明并获伦理委员会批准,同时应尽快获得继续参加的知情同意。

12.4 受试者的医疗和保护

参与临床试验的医生应对所有与试验相关的医学决策负责。在试验及随访期间,如受试者出现与试验相关的不良事件或有临床意义的实验室异常情况,研究者及所在医疗机构必须确保受试者得到适当的医疗处理,并如实告知受试者相关情况;同时研究者还应警惕受试者是否存在其他潜在疾病。

在获得受试者同意的前提下,研究者可将其参加试验的情况告知其主治医生,以获取更全面的医疗信息。

受试者有权利随时无理由退出临床试验。在尊重受试者个人权利的同时,研究者应努力了解其退出的原因,以完善后续试验设计和操作流程。

保护受试者权益是临床试验的重中之重,研究者及试验机构有责任为受试者提供足够的医疗保障,并尊重其自主决定权,确保试验在安全、合乎伦理的环境下顺利进行。

12.5 受试者隐私的保护

受试者个人医疗记录的访问将受到严格限制,只有直接参与本临床试验的研究人员和监查员有权接触,他们需签署"研究者声明"或"保密承诺",承诺遵守保密条款。药品监管部门在执法监管过程中,有权查阅临床试验相关记录。

在数据处理环节,将采用匿名化方式,删除可能导致受试者个体身份识别的所有信息,以保护受试者的隐私权。受试者的所有临床试验资料将由医院指定的相关管理部门专门保管。

此外,参与临床试验的所有相关人员,都有义务恪守职业操守,对于在试验过程中接触到的受试者个人隐私信息严格保密,确保受试者的个人隐私和数据安全得到充分保护,维护受试者的合法权益。

12.6 招募受试者

招募方式包括从临床医疗过程中直接招募、公开招募、通过数据库进行筛选招募等。招募广告应位于临床环境、公共场所和网络平台，以确保足够的样本增加。

13.质量保证和质量控制

实施良好的质量控制对于确保临床试验数据的有效性和安全性至关重要。在临床试验开展之前,必须预先考虑并采取相应措施来应对可能影响试验质量的潜在问题。申办方有责任制定、实

施并及时更新相关的标准操作规程(SOP),构建完善的质量保证和质量控制体系,以确保临床试验的实施过程、数据的产生、记录和报告等各个环节均严格遵循试验方案、良好临床实践(GCP)规范以及相关法律法规的要求,从而保证试验质量。

13.1 主观症状评价质量控制

为确保不同研究者对症状量表评价的一致性,需在试验实施前制定统一的评价标准,并对所有研究人员进行统一的培训,并通过一致性测试检验。培训对象包括各中心的主要研究者、研究员、用药管理人员、实验室人员、护理人员等参与人员,使他们充分理解方案及相关指南要求。

13.2 实验室检查指标质量控制

参与临床试验的医疗机构实验室,应当建立质量管理标准和操作规范,以保证检测数据和结果的准确可靠性。

13.3 临床监查/稽查质量控制

申办方应指派受过相应培训、具备临床试验监查所需科学和临床知识、持有资质证明的监查员。申办方需制定监查计划和标准操作规程(SOP),监查员在监查工作中须遵照执行。

14. 资料保存

研究者及其供职的医疗机构应按“临床试验必备文件”和药品监督管理部门的相关要求，妥善保存试验文档，必备文件根据临床试验不同阶段归类为三个阶段：临床试验准备阶段、临床试验进行阶段、临床试验完成后阶段。必备文件包括对所有受试者情况真实和准确的确认（能有效地核对不同的记录资料，如医院的原始记录）、所有原始的有受试者签名的知情同意书、所有病历登记表的详细记录等。全部病例无论是观察完成还是脱落，均应按要求完成研究病历的填写。研究病历作为原始资料由各参加单位自行存档；保存知情同意书、研究病历至或者临床试验终止后 5 年（以最长者为准）。

15. 参考文献

1. Y L, L Z, S L, X W, J Z, X T, et al. Efficacy and safety of oral Guanxinshutong capsules in patients with stable angina pectoris in China: a prospective, multicenter, double-blind, placebo-controlled, randomized clinical trial. BMC complementary and alternative medicine. 2019;19. doi:10.1186/s12906-019- 2778-z
2. P H, Z L, L C, J Z, S Z, Y T, et al. The comparative effects of oral Chinese patent medicines combined with western medicine in stable angina: A systematic review and network meta-analysis of 179 trials. Frontiers in pharmacology. 2022;13. doi:10.3389/fphar.2022.918689
3. J W, W G, G C, M C, Z W, W Z, et al. Biomarker-based risk model to predict cardiovascular events in patients with acute coronary syndromes - Results from BIPass registry. The Lancet regional health Western Pacific. 2022;25. doi:10.1016/j.lanwpc.2022.100479

4. H H, Az J, Mh H, E V, Sm M, A M. The effects of Melissa officinalis supplementation on depression, anxiety, stress, and sleep disorder in patients with chronic stable angina. Clinical nutrition ESPEN. 2018;26. doi:10.1016/j.clnesp.2018.04.015
5. Y W, Y Z, Y D, Y Y, J W, N Z, et al. A multicenter, randomized, double-blind, placebo-controlled trial evaluating the efficacy and safety of Taoren Honghua Jian granule in patients with stable coronary artery disease. Medicine. 2019;98. doi:10.1097/MD.0000000000017753
6. M S-L, Eb L, B L, Ma C, K S, Ml S, et al. Sleep duration, insomnia, and coronary heart disease among postmenopausal women in the Women's Health Initiative. Journal of women's health (2002). 2013;22. doi:10.1089/jwh.2012.3918
7. M S, M Z, L H, D F, P S. Assessment of the climacteric syndrome: a narrative review. Archives of gynecology and obstetrics. 2021;304. doi:10.1007/s00404-021-06139-y
8. A M, L A, Av P. Is post-ischemic stroke insomnia related to a negative functional and cognitive outcome? Sleep medicine. 2022;94. doi:10.1016/j.sleep.2022.03.022
9. P M, A S, D S. Restricted and disrupted sleep: effects on autonomic function, neuroendocrine stress systems and stress responsivity. Sleep medicine reviews. 2008;12. doi:10.1016/j.smrv.2007.07.007
10. B S, T L, P S, S P, M H, S K, et al. Insomnia as a risk factor for ill health: results from the large population-based prospective HUNT Study in Norway. Journal of sleep research. 2014;23. doi:10.1111/jsr.12102
11. Rq L, Z Q, E T, Jj C, A Z, Yt H, et al. Poor sleep quality associated with high risk of hypertension and elevated blood pressure in China: results from a large population-based study. Hypertension research : official journal of the Japanese Society of Hypertension. 2016;39. doi:10.1038/hr.2015.98
12. Vr T, S S, A M, A T. Menopause and Sleep Disorders. Journal of mid-life health. 2022;13. doi:10.4103/jmh.jmh_18_22
13. Cm M, Dc J. Epidemiology of Insomnia: Prevalence, Course, Risk Factors, and Public Health Burden. Sleep medicine clinics. 2022;17. doi:10.1016/j.jsmc.2022.03.003
14. Lr R, M L-P, Dr P, Ne E, Dm L, Ml K. Detoxification from high-dose zolpidem using diazepam. The Annals of pharmacotherapy. 2004;38. doi:10.1345/aph.1D339
15. C L, Y Z, S Q, X W, Y J, W W. Randomized controlled trial of acupuncture for anxiety and depression in patients with chronic insomnia. Annals of translational medicine. 2021;9. doi:10.21037/atm-21-3845
16. Zhou L, Hu X, Yu Z, Yang L, Wan R, Liu H, et al. Efficacy and Safety of Acupuncture in the Treatment of Poststroke Insomnia: A Systematic Review and Meta-Analysis of Twenty-Six Randomized Controlled Trials. Seong Lin T, editor. Evidence-Based Complementary and

Alternative Medicine. 2022;2022: 1–16. doi:10.1155/2022/5188311

17. Y L, H Z, Q W, C T, H L, L H, et al. Comparative effectiveness of multiple acupuncture therapies for primary insomnia: a systematic review and network meta-analysis of randomized trial. Sleep medicine. 2022;93. doi:10.1016/j.sleep.2022.03.012
18. Effects of electroacupuncture at "Anmian (Extra)" acupoints on sleep activities in rats: the implication of the caud al nucleus tractus solitarius - PubMed. [cited 13 Nov 2023]. Available: https://pubmed.99885.net/15316132/
19. Sy S, Jy M, Sy K, Os K, Sk B, Kh C, et al. Acupuncture stimulation at HT7 as a non-pharmacological therapy for sleep disorder caused by caffeine administration in rats. Acupuncture in medicine : journal of the British Medical Acupuncture Society. 2021;39. doi:10.1177/09645284211011489
20. Efficacy and safety of acupuncture treatment on primary insomnia: a randomized controlled trial - PubMed. [cited 13 Nov 2023]. Available: https://pubmed.99885.net/28899535/
21. Mao LL, Jin YP. [Professor JIN Ya-pei's experience in the treatment of perimenopausal insomnia with navel acupuncture combined with body acupuncture]. Zhongguo Zhen Jiu. 2020;40(3):295-8
22. C F, N Z, Z L, Lh Y, C X, Wj Y, et al. Acupuncture Improves Peri-menopausal Insomnia: A Randomized Controlled Trial. Sleep. 2017;40. doi:10.1093/sleep/zsx153
23. Li O, Wang F. [Acupuncture at back-shu points of five zang, Geshu (BL 17) and Shenmen (HT 7) for the treatment of menopausal insomnia]. Zhongguo Zhen Jiu. 2018;38(5):4693-72
24. T N, M H, Sn G. Assessment of the Frequency and Variety of Persistent Symptoms Among Patients With COVID-19: A Systematic Review. JAMA network open. 2021;4. doi:10.1001/jamanetworkopen.2021.11417
25. A T, Sm GH, H T, A A, M I, A S, et al. COVID-19 and cardiac injury: clinical manifestations, biomarkers, mechanisms, diagnosis, treatment, and follow up. Expert review of anti-infective therapy. 2021;19. doi:10.1080/14787210.2020.1822737

附件 1 匹兹堡睡眠质量指数

指导语：下面一些问题是关于您最近 1 个月的睡眠情况，请选择回填写最符合您近 1 个月实际情况的答案。请回答下列问题！

1. 近 1 个月，晚上上床睡觉通常—— 点钟。

2. 近 1 个月，从上床到入睡通常需要——分钟。

3. 近 1 个月，通常早上——点起床

4. 近 1 个月，每夜通常实际睡眠——小时(不等于卧床时间)。　　对下列问题请选择 1 个最适合您的答案。

5. 近 1 个月 ，因下列情况影响睡眠而烦恼：　　a. 入睡困难(30 分钟内不能入睡)(1)无(2)〈1 次/周 (3)1-2 次/周 (4)≥ 3 次/周　　b. 夜间易醒或早醒 (1)无(2)〈1 次/周(3)1-2 次/周(4)≥ 3 次/周　　c. 夜间去厕所 (1)无(2)〈1 次/周(3)1-2 次/周(4)≥ 3 次/周　　d. 呼吸不畅 (1)无(2)〈1 次/周(3)1-2 次/周(4)≥ 3 次/周　　e. 咳嗽或鼾声高 (1)无(2)〈1 次/周(3)1-2 次/周(4)≥3 次/周　　f. 感觉冷 (1)无(2)〈1 次/周(3)1-2 次/周(4)≥3 次/周 g. 感觉热 (1)无(2)〈1 次/周(3)1-2 次/周(4)≥3 次/周　　h. 做恶梦 (1)无(2)〈1 次/周(3)1-2 次/周(4)≥ 3 次/周　　i. 疼痛不适 (1)无(2)〈1 次/周(3)1-2 次/周(4)≥ 3 次/周 j. 其它影响睡眠的事情 (1)无(2)〈1 次/周(3)1-2 次/周(4)≥ 3 次/周　　如有，请说明：

6. 近 1 个月，总的来说，您认为自己的睡眠质量(1)很好(2)较好(3)较差 (4)很差

7. 近 1 个月，您用药物催眠的情况(1)无(2)〈1 次/周(3)1-2 次/周 (4)≥ 3 次/周

8. 近 1 个月，您常感到困倦吗 (1)无(2)〈1 次/周(3)1-2 次/周 (4)≥ 3 次/周

9. 近 1 个月，您做事情的精力不足吗(1)没有(2)偶尔有(3)有时有(4)经常有

使用和统计方法

PSQI 用于评定被试最近 1 个月的睡眠质量．由 19 个自评和 5 个他评条目构成，其中第 19 个自评条目和 5 个他评条目不参与计分,在此仅介绍参与计分的 18 个自评条目(详见附问卷).18 个条目组成 7 个成份，每个成份按 o～3 等级计分，累积各成份得分为 PSQI 总分，总分范围为 0~21，得分越高，表示睡眠质量越差。被试者完成试问需要 5～10 分钟。　　各成份含意及计分方法如下：

A 睡眠质量：根据条目 6 的应答计分“较好”计 1 分，“较差”计 2 分，“很差”计 3 分。

B 入睡时间　　1. 条目 2 的计分为“≤15 分”计 0 分，“16～30 分”计 1 分，“31~60”计 2 分，“≥　　60 分”计 3 分。　　2. 条目 5a 的计分为“无”计 0 分，“<1 周/次”计 1 分，“1~2 周/次”计 2 分，“≥3 周/次”计 3 分。　　3. 累加条目 2 和 5a 的计分，若累加分为“0”计 0 分，“1~2”计 1 分，“3~4”计 2 分，“5~6”计 3 分

C 睡眠时间　　根据条目 4 的应答计分，“>7 小时”计 0 分，“6~7”计 1 分，“5~6”计 2 分，“<5 小时”计 3 分。

D 睡眠效率　　1. 床上时间 = 条目 3（起床时间）- 条目 1（上床时间）　　2. 睡眠效率 = 条目 4（睡眠时间）/ 床上时间 × 100%　　3. 成分 D 计分位，睡眠效率 > 85%计 0 分，75~84% 计 1 分，65~74%计 2 分，< 65% 计 3 分。

E 睡眠障碍　　根据条目 5b 至 5j 的计分为“无”计 0 分，“<1 周/次”计 1 分，“1~2 周/次”计 2 分，“≥3 周/次”计 3 分。累加条目 5b 至 5j 的计分，若累加分为“0”则成分 E 计 0 分，“1~9”计 1 分，“10~18”计 2 分，“19~27”计 3 分。

F 催眠药物　　根据条目 7 的应答计分，“无”计 0 分，“<1 周/次”计 1 分，“1~2 周/次”计 2 分，“≥3 周/次”计 3 分。

G 日间功能障碍：　　1. 根据条目 8 的应答计分，“无”计 0 分，“<1 周/次”计 1 分，“1~2 周/次”计 2 分，“≥3 周/次”计 3 分。。　　2. 根据条目 9 的应答计分，“没有”计 0 分，“偶尔有”计 1 分，“有时有”计 2 分，“经常有”计 3 分。　　3. 累加条目 8 和 9 的得分，若累加分为“0”则成分 G 计 0 分，“1~2”计 1 分，“3~4”计 2 分，“5~6”计 3 分

PSQI 总分 = 成分 A + 成分 B + 成分 C + 成分 D + 成分 E + 成分 F + 成分 G

附件 2

SF-36 生活质量表调查表

编号：　　姓名：　　　性别：　　年龄　　　　管理类别：

1、总体来讲，您的健康状况是：

①非常好　②很好　　③好　　④一般　　　⑤差

（权重或得分依次为 5、4、3、2、1）

2、跟 1 年以前比您觉得自己的健康状况是：

①比 1 年前好多了　　②比 1 年前好一些　　③跟 1 年前差不多　　④比 1 年前差一些 ⑤比 1 年前差多了（权重或得分依次为 5、4、3、2、1）

健康和日常活动

3、以下这些问题都和日常活动有关。请您想一想，您的健康状况是否限制了这些活动？如果有限制，程度如何？

（1）重体力活动。如跑步举重、参加剧烈运动等：

①限制很大　②有些限制　　③毫无限制

（权重或得分依次为 1，2，3；下同）

（2）适度的活动。如移动一张桌子、扫地、打太极拳、做简单体操等：

①限制很大　②有些限制　　③毫无限制

（3）手提日用品。如买菜、购物等：

①限制很大　②有些限制　　③毫无限制

（4）上几层楼梯：①限制很大　②有些限制　　③毫无限制

（5）上一层楼梯：①限制很大　②有些限制　　③毫无限制

（6）弯腰、屈膝、下蹲：①限制很大　②有些限制　　③毫无限制

（7）步行 1500 米以上的路程：①限制很大　②有些限制　③毫无限制

（8）步行 1000 米的路程：①限制很大　②有些限制　　③毫无限制

（9）步行 100 米的路程：①限制很大　②有些限制　　③毫无限制

（10）自己洗澡、穿衣：①限制很大　②有些限制　　③毫无限制

4、在过去 4 个星期里，您的工作和日常活动有无因为身体健康的原因而出现以下这些问题？

（1）减少了工作或其他活动时间：①是　②不是

（权重或得分依次为 1，2；下同）

（2）本来想要做的事情只能完成一部分： ①是 ②不是

（3）想要干的工作或活动种类受到限制： ①是 ②不是

（4）完成工作或其他活动困难增多（比如需要额外的努力)：①是②不是

5、在过去 4 个星期里，您的工作和日常活动有无因为情绪的原因（如压抑或忧虑）而出现以下这些问题？

（1）减少了工作或活动时间：①是 ②不是

（权重或得分依次为 1，2；下同）

（2）本来想要做的事情只能完成一部分： ①是 ②不是

（3）干事情不如平时仔细： ①是 ②不是

6、在过去 4 个星期里，您的健康或情绪不好在多大程度上影响了您与家人、朋友、邻居或集体的正常社会交往？

①完全没有影响 ②有一点影响 ③中等影响 ④影响很大 ⑤影响非常大

（权重或得分依次为 5，4，3，2，1）

7、在过去 4 个星期里，您有身体疼痛吗？

①完全没有疼痛 ②有一点疼痛 ③中等疼痛 ④严重疼痛 ⑤很严重疼痛

（权重或得分依次为 6，5.4，4.2，3.1，2.2，1）

8、在过去 4 个星期里，您的身体疼痛影响了您的工作和家务吗？

①完全没有影响 ②有一点影响 ③中等影响 ④影响很大 ⑤影响非常大

（如果 7 无 8 无，权重或得分依次为 6，4.75，3.5，2.25，1.0；如果为 7 有 8 无，则为 5，4，3，2，1）

您的感觉

9、以下这些问题是关于过去 1 个月里您自己的感觉，对每一条问题所说的事情，您的情况是什么样的？

（1）**您觉得生活充实：**

①所有的时间 ②大部分时间 ③比较多时间 ④一部分时间 ⑤小部分时间 ⑥没有这种感觉

（权重或得分依次为 6，5，4，3，2，1）

（2）您是一个敏感的人：

①所有的时间 ②大部分时间 ③比较多时间 ④一部分时间 ⑤小部分时间 ⑥没有这种感觉

（权重或得分依次为 1，2，3，4，5，6）

（3）您的情绪非常不好，什么事都不能使您高兴起来：

①所有的时间　②大部分时间　③比较多时间　④一部分时间　⑤小部分时间　⑥没有这种感觉　（权重或得分依次为 1，2，3，4，5，6）

（4）您的心理很平静：

①所有的时间　②大部分时间　③比较多时间　④一部分时间　⑤小部分时间　⑥没有这种感觉　（权重或得分依次为 6，5，4，3，2，1）

（5）您做事精力充沛：

①所有的时间　②大部分时间　③比较多时间　④一部分时间　⑤小部分时间　⑥没有这种感觉　（权重或得分依次为 6，5，4，3，2，1）

（6）您的情绪低落：

①所有的时间　②大部分时间　③比较多时间　④一部分时间　⑤小部分时间　⑥没有这种感觉　（权重或得分依次为 1，2，3，4，5，6）

（7）您觉得筋疲力尽：

①所有的时间　②大部分时间　③比较多时间　④一部分时间　⑤小部分时间　⑥没有这种感觉　（权重或得分依次为 1，2，3，4，5，6）

（8）您是个快乐的人：

①所有的时间　②大部分时间　③比较多时间　④一部分时间　⑤小部分时间　⑥没有这种感觉　（权重或得分依次为 6，5，4，3，2，1）

（9）您感觉厌烦：

①所有的时间　②大部分时间　③比较多时间　④一部分时间　⑤小部分时间　⑥没有这种感觉　（权重或得分依次为 1，2，3，4，5，6）

10、不健康影响了您的社会活动（如走亲访友）：

①所有的时间　②大部分时间　③比较多时间　④一部分时间　⑤小部分时间　⑥没有这种感觉　（权重或得分依次为 1，2，3，4，5）

总体健康情况

11、请看下列每一条问题，哪一种答案最符合您的情况？

（1）我好象比别人容易生病：

①绝对正确　②大部分正确　③不能肯定　④大部分错误　⑤绝对错误`

（权重或得分依次为 1，2，3，4，5）

（2）我跟周围人一样健康：

①绝对正确　②大部分正确　③不能肯定　④大部分错误　⑤绝对错误

（权重或得分依次为5，4，3，2，1）

（3）我认为我的健康状况在变坏：

①绝对正确　②大部分正确　③不能肯定　④大部分错误　⑤绝对错误

（权重或得分依次为1，2，3，4，5）

（4）我的健康状况非常好：

①绝对正确　②大部分正确　③不能肯定 ④大部分错误　⑤绝对错误

（权重或得分依次为5，4，3，2，1）

附表 3 ：汉密尔顿抑郁量表(HAMD)

汉密尔顿抑郁量表(Hamilton Depression Scale，HAMD)是由 Hamilton 编制，是临床上评定抑郁状态时应用得最为普遍的量表。量表是 24 项版本，方法简单，标准明确，便于掌握。适用于有抑郁症状的成人。总分能够较好的反映疾病严重程度，也能很好的衡量治疗效果，是经典和被公认的抑郁评定量表。

量表内容：

1、抑郁情绪　选择以下 1~4 分。

(1)只在问到时才诉述;

(2)在访谈中自发地表达;

(3)不用言语也可从表情、姿势、声音或欲哭中流露出这种情绪;

(4)患者的自发言语和非语言表达(表情、动作)几乎完全表现为这种情绪。

2、有罪感

(1)责备自己，感到自己已连累他人;

(2)认为自己犯了罪，或反复思考以往的过失和错误;

(3)认为目前的疾病，是对自己错误的惩罚，或有罪恶妄想;

(4)罪恶妄想伴有指责或威胁性幻觉。

3、自杀

(1)觉得活着没有意义;

(2)希望自己已经死去，或常想到与死有关的事;

(3)消极观念(自杀念头);

(4)有自杀行为。

4、入睡困难　初段失眠。

(1)主诉有入睡困难，上床半小时后仍不能入睡。(要注意患者平时入睡的时间);

(2)主诉每晚均有入睡困难。

5、睡眠不深　中段失眠。

(1)睡眠浅，多恶梦;

(2)半夜(晚 12 点钟以前)曾醒来(不包括上厕所)。

6、早醒　末段睡眠。

(1)有早醒，比平时早醒 1h，但能重新入睡(应排除平时的习惯);

(2)早醒后无法重新入睡。

7、工作和兴趣

(1)提问时才诉述;

(2)自发地直接或间接表达对活动、工作或学习失去兴趣，如感到没精打彩，犹豫不决，不能坚持或强迫自己去工作或活动;

(3)活动时间减少或成效下降，住院患者每天参加病房劳动或娱乐不满 3h;

(4)因目前的疾病而停止工作，住院者不参加任何活动或没有他人帮助便不能完成病室日常事物(注意不能凡住院就打 4 分)。

8、阻滞　指思想和言语缓慢，注意力难以集中，主动性减退。

(1)精神检查中发现轻度阻滞;

(2)精神检查发现明显阻滞;

(3)精神检查进行困难;

(4)完全不能回答问题(木僵)。

9、激越

(1)检查时有些心神不宁;

(2)明显心神不宁或小动作多;

(3)不能静坐检查中曾起立;

(4)搓手、咬手指、扯头发、咬嘴唇。

10、精神性焦虑

(1)问时诉述;

(2)自发地表达;

(3)表情和言语流露出明显忧虑;

(4)明显惊恐。

11、躯体性焦虑　指焦虑的生理症状，包括：口干、腹胀、腹泻、打嗝、腹绞痛、心悸、头痛、过度换气和叹气，以及尿频和出汗。

(1)轻度;

(2)中度，有肯定的上述症状;

(3)重度，上述症状严重，影响生活或需要处理;

(4)严重影响生活和活动。

12、胃肠道症状

(1)食欲减退，但不需他人鼓励便自行进食;

(2)进食需他人催促或请求和需要应用泻药或助消化药。

13、全身症状

(1)四肢、背部或颈部沉重感，背痛、头痛、肌肉疼痛，全身乏力或疲倦;

(2)症状明显。

14、性症状　指性欲减退，月经紊乱等。

(1)轻度;

(2)重度;

(3)不能肯定，或该项对被评者不适合(不计入总分)。

15、疑病

(1)对身体过分关注;

(2)反复考虑健康问题;

(3)有疑病妄想;

(4)伴有幻觉的疑病妄想。

16、体重减轻

(1)按病史评定：①患者诉述可能有体重减轻;②肯定体重减轻。

(2)按体重记录评定：①1 周内体重减轻超过 0.5kg;②1 周内体重减轻超过 1kg。

17、自知力

(1)知道自己有病，表现为忧郁;

(2)知道自己有病，但归咎伙食太差，环境问题，工作过忙，病毒感染或需要休息;

(3)完全否认有病。

评定方法：

HAMD 大部分项目采用 0~4 分的 5 级评分法。各级的标准为：(0)无;(1)轻度;(2)中度;(3)重度;(4)极重度。少数项目采用 0~2 分的 3 级评分法，其分级的标准为：(0)无;(1)轻~中度;(2)重度。

结果判定：

(1)总分超过 24 分为严重抑郁，超过 17 分为轻或中度抑郁，小于 7 分无抑郁症状。

(2)因子分：HAMD 可归纳为 7 类因子结构：①焦虑躯体化：由精神性焦虑、躯体性焦虑、胃肠道症状、疑病和自知力等 5 项组成(即 10、11、12、15、17 项);②体重：即体重减轻一项(第 16 项);③认知障碍：由自罪感、自杀、激越等组成(2、3、9 项);④阻滞：由抑郁情绪、工作和兴趣、阻滞和性症状等 4 项组成(1、7、8、14 项);⑤睡眠障碍：由入睡困难、睡眠不深和早醒等 3 项组成(4~6 项)。

通过因子分析，不仅可以具体反映患者的病情特点，也可反映靶症状群的临床结果。

注意事项：

(1)评定的时间范围为过去 1 周内。

(2)由两名医师采用交谈与观察的方式进行检查，检查结束后，两名评定者分别独立评分。

(3)如用以疗效评估，应在开始治疗前和在治疗后各评定 1 次，以便对比疗效。

(4)HAMD 中，第 8、9 及第 11 项依据对患者的观察进行评定;其余各项则根据患者自己的口头叙述评分;其中第 1 项需两者兼顾。此外，第 7 项尚需向患者家属或病房工作人员收集资料;第 16 项最好根据体重记录，也可依据患者主诉及其家属或病房工作人员所提供的资料评定。

附件 4　西雅图心绞痛量表

1.过去 4 周，由于胸痛、胸部紧榨感或心绞痛导致下列活动的受限程度	
1.1 自己穿衣	□$_1$严重受限　□$_2$ 中度受限　□$_3$ 轻度受限<br>□$_4$稍微受限　□$_5$ 不受限　□$_6$ 因其他原因受限
1.2 室内散步	□$_1$严重受限　□$_2$ 中度受限　□$_3$ 轻度受限<br>□$_4$稍微受限　□$_5$ 不受限　□$_6$ 因其他原因受限
1.3 淋浴	□$_1$严重受限　□$_2$ 中度受限　□$_3$ 轻度受限<br>□$_4$稍微受限　□$_5$ 不受限　□$_6$ 因其他原因受限
1.4 爬坡或爬楼梯（三层，不停）	□$_1$严重受限　□$_2$ 中度受限　□$_3$ 轻度受限<br>□$_4$稍微受限　□$_5$ 不受限　□$_6$ 因其他原因受限
1.5 户外运动或搬运杂物	□$_1$严重受限　□$_2$ 中度受限　□$_3$ 轻度受限<br>□$_4$稍微受限　□$_5$ 不受限　□$_6$ 因其他原因受限
1.6 快走（1 公里）	□$_1$严重受限　□$_2$ 中度受限　□$_3$ 轻度受限<br>□$_4$稍微受限　□$_5$ 不受限　□$_0$ 因其他原因受限
1.7 慢跑（1 公里）	□$_1$严重受限　□$_2$ 中度受限　□$_3$ 轻度受限<br>□$_4$稍微受限　□$_5$ 不受限　□$_6$ 因其他原因受限
1.8 搬运或移动重物	□$_1$严重受限　□$_2$ 中度受限　□$_3$ 轻度受限<br>□$_4$稍微受限　□$_5$ 不受限　□$_6$ 因其他原因受限
1.9 剧烈运动（例如游泳、网球）	□$_1$严重受限　□$_2$ 中度受限　□$_3$ 轻度受限<br>□$_4$稍微受限　□$_5$ 不受限　□$_6$ 因其他原因受限
2.与 4 周前相比，您做最大强度运动时感到胸痛、胸部紧榨感或心绞痛的频率<br>□$_1$明显增加　□$_2$轻微增加　□$_3$相同　□$_4$轻微减少　□$_5$明显减少	
3. 过去 4 周内，胸痛、胸部紧榨感和心绞痛的平均发作次数<br>□$_1$≥4次/天　□$_2$1-3次/天　□$_3$≥3次/周<br>□$_4$1-2次/周　□$_5$＜1次/周　□$_6$无发作	
4.过去 4 周，胸痛、胸部紧榨感和心绞痛是服用硝酸甘油的平均次数<br>□$_1$≥4次/天　□$_2$1-3次/天　□$_3$≥3次/周<br>□$_4$1-2次/周　□$_5$＜1次/周　□$_6$没使用	
5.因胸痛、胸部紧榨感和心绞痛遵守医嘱服药带来的烦恼<br>□$_1$严重　□$_2$中度　□$_3$轻微　□$_4$极少　□$_5$无　□$_6$医生未给药	
6.对治疗胸痛、胸部紧榨感和心绞痛的各种措施的满意程度<br>□$_1$不满意　□$_2$大部分不满意　□$_3$部分满意　□$_4$大部分满意　□$_5$非常满意	
7.对医生就胸痛、胸部紧榨感和心绞痛的解释满意程度<br>□$_1$不满意　□$_2$大部分不满意　□$_3$部分满意　□$_4$大部分满意　□$_5$非常满意	
8.总的来说，对目前胸痛、胸部紧榨感和心绞痛的治疗满意程度<br>□$_1$不满意　□$_2$大部分不满意　□$_3$部分满意　□$_4$大部分满意　□$_5$非常满意	
9.过去 4 周内，胸痛、胸部紧榨感和心绞痛影响生活乐趣的程度<br>□$_1$严重　□$_2$中度　□$_3$轻微　□$_4$极少　□$_5$无	
10.在您的未来生活中如果还有胸痛、胸部紧榨感和心绞痛，您会感觉怎样<br>□$_1$不满意　□$_2$大部分不满意　□$_3$部分满意　□$_4$大部分满意　□$_5$非常满意	

11. 对心脏病发作和突然死亡的担心程度
□1一直担心　□2经常担心　□3有时担心　□4很少担心　□5不担心

附件 5 广泛性焦虑量表(GAD-7)

一、简介

1、测评方式：自评

2、量表功能：广泛性焦虑量表 GAD-7 是广泛用于临床的量表，用于评估焦虑情绪，定期（1次/1-2 周）自评可以观察焦虑情绪变化趋势和治疗效果。

3、适用人群:各种人群。

4、测评时长:1-3 分钟

二、具体测试

量表介绍:

测评指导：根据过去的两周的状况，请您回答是否存在下列描述的状况及频率，请看清楚问题后选择符合您的选项。

题目				
感觉紧张，焦虑或急切	完全不会	好几天	超过一周	几乎每天
不能够停止或控制担忧	完全不会	好几天	超过一周	几乎每天
对各种各样的事情担忧过多	完全不会	好几天	超过一周	几乎每天
很难放松下来	完全不会	好几天	超过一周	几乎每天
由于不安而无法静坐	完全不会	好几天	超过一周	几乎每天
变得容易烦恼或急躁	完全不会	好几天	超过一周	几乎每天
感到似乎将有可怕的事情发生而害怕	完全不会	好几天	超过一周	几乎每天

三、使用指南

1、计分方法：广泛性焦虑量表 GAD-7 共有 7 个条目。每个条目分 4 级，分别为 3=几乎每天;2=超过一周;1=好几天;0=完全不会，总分就是将 7 个条目的分值相加，总分值范围 0-21 分。

焦虑程度分析表

总分	分析
0-4 分	正常水平
5-9 分	轻度焦虑

10-13 分	中度焦虑
14-18 分	中重度焦虑
19-21 分	重度焦虑

附录 6 穴位和非穴位的分配

穴位	地点	非穴位	地点
PC6（内官）	在前臂前部，掌长肌腱和桡侧屈腕肌腱之间，腕部掌侧远端横纹上方 2 厘米处（约 50 毫米）。	非尖点 1	在前臂前区，掌长肌腱尺侧端，腕部远侧掌侧横纹上方 2 厘米处（约 50 毫米）。避开手厥阴肝经、手太阴肺经和正中神经。
EX-HN3 （银塘）	在人体前额，两眉连线与前正中线的交点处。	非尖点 2	在身体前额，与 EX-HN3 位于同一水平线上，前正中线右侧 0.5 厘米（约 15 毫米）处，避开任脉和足太阳膀胱经，同时避开额动脉。
KI6 （昭海）	在人体踝部，内踝顶端下方 1 英寸（约 25 毫米）处，内踝下缘的边缘凹陷处。	非尖点 3	在人体踝关节内踝尖下 2 cun（约 50mm）处，避开足厥阴肝经和足少阴肾经，同时避开胫神经。
RN17（丹中）	它位于前正中线，胸骨体上，第 4 肋间隙水平，两乳头连线的中点。	非尖点 4	它位于人体前胸，与 RN17 位于同一水平线上，在前正中线右侧 0.5 厘米（约 15 毫米）处，避开任脉和肋间神经。
HT3（少海）	它位于前臂的前内侧，肘横纹与肱骨内上髁连线的中点。	非尖点 5	位于前臂前内侧，肘横纹与肱骨内上髁连线的中点，肘横纹上 2 厘米（约 50 毫米）处。避开手少阴心经和手太阴肺经，以及正中神经和尺神经。
SP6 （三元角）	在小腿内侧，内踝尖上方 3 厘米（约 75 毫米）处，胫骨内侧缘后方。	非尖点 6	小腿内侧，内踝尖上 3 厘米（约 75 毫米），胫骨内侧缘后方 1 英寸（约 25 毫米）处。避开足太阴脾经和足少阴肾经。避开胫神经和髂后静脉运行的区域。

附录 7 针灸和非穿刺器械两组示意图

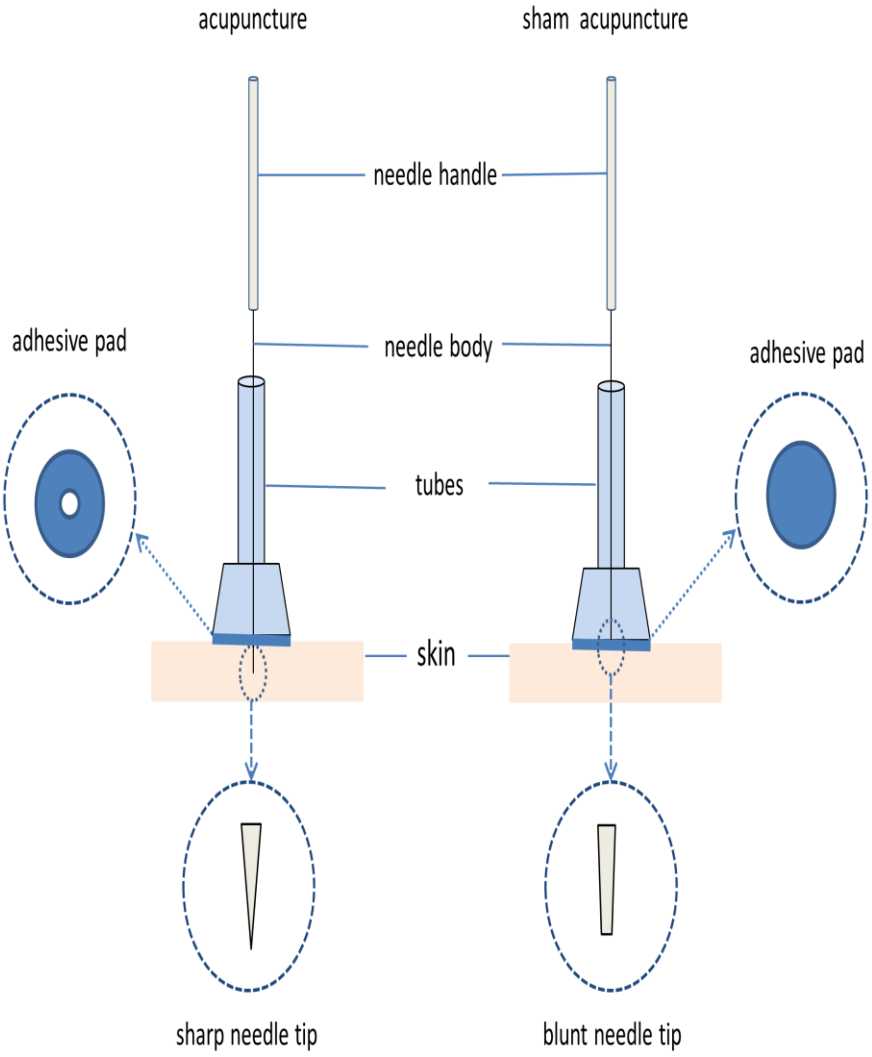

Supplement: S2 File — (PDF) [file pone.0301827.s004.pdf]
