# Supplementary material for: Exploring acupuncture as a treatment for insomnia in perimenopausal women with stable angina pectoris: A protocol for a randomized, double-blind, placebo-controlled clinical trial
Source: PLoS One. 2024 Apr 18;19(4):e0301827. doi: 10.1371/journal.pone.0301827 (PMC11025937; doi:10.1371/journal.pone.0301827)
Supplement: S1 Table — (PDF) [file pone.0301827.s005.pdf]

S1 Table Allocation of Acupoints and Non-acupoints.

Acupoint	Location	Non-acupoint	Location
PC6 (Neiguan)	In the anterior region of the forearm, between the tendon of the palmaris longus and the tendon of the radial flexor carpi radialis, 2 cun above the transverse stripe on the distal part of the palmar side of the wrist (about 50 mm).	Non-acupoint 1	In the anterior region of the forearm, at the ulnar end of the tendon of the palmaris longus, 2 cun above the transverse stripe on the distal palmar side of the wrist (about 50 mm). Avoid the hand syncopal meridian, hand taiyin lung meridian, and median nerve.
EX-HN3 (Yintang)	On the forehead of the human body, at the intersection of the line connecting the two eyebrows and the front center line.	Non-acupoint 2	On the forehead of the body, at the same level as the EX-HN3, 0.5 cun (about 15 mm) to the right of the anterior midline, avoiding the Ren and Foot Solar Bladder meridians, as well as avoiding the frontal artery.
KI6 (Zhaohai)	In the human ankle, 1 inch (about 25mm) below the tip of the inner ankle, in the marginal depression of the lower edge of the inner ankle.	Non-acupoint 3	In the human ankle, 2 cun (about 50mm) below the tip of the inner ankle, avoiding the foot syncopal liver meridian and foot shaoyin kidney meridian, as well as avoiding the tibial nerve.
RN17 (Danzhong)	It is located in the anterior midline, on the body of the sternum, at the level of the 4th intercostal space, at the midpoint of the line between the two nipples.	Non-acupoint 4	It is located in the anterior chest of the body, at the same level as RN17, 0.5 cun (about 15 mm) to the right of the anterior median line, avoiding the Ren Chakra, as well as the intercostal nerve.
HT3 (Shaohai)	It is located on the anterior medial side of the forearm, at the midpoint of the line between the transverse elbow stripe and the medial epicondyle of the humerus.	Non-acupoint 5	It is located on the anterior medial side of the forearm, at the midpoint of the line between the transverse elbow stripe and the medial epicondyle of the humerus, 2 cun (about 50 millimeters) above the transverse elbow stripe. Avoiding the Shaoyin Pericardium Meridian and the Taiyin Lung Meridian of the hand, as well as the median nerve and ulnar nerve.
SP6 (Sanyinjiao)	On the medial side of the lower leg, 3 cun (about 75 mm) above the tip of the inner ankle, behind the medial border of the tibia.	Non-acupoint 6	Medial side of the calf, 3 cun (about 75 mm) above the tip of the inner ankle and 1 inch (about 25 mm) behind the medial border of the tibia. Avoid the foot-taiyin spleen meridian and foot-shaoyin kidney meridian. Avoid the area where the tibial nerve and posterior iliac vein run.
